# Heterosis for Resistance to Insect Herbivores in a 3-Line Hybrid Rice System

**DOI:** 10.3390/insects15030164

**Published:** 2024-02-28

**Authors:** Finbarr G. Horgan, Carmencita C. Bernal, Angelee F. Ramal, Maria Liberty P. Almazan, Enrique A. Mundaca, Eduardo Crisol-Martínez

**Affiliations:** 1 EcoLaVerna Integral Restoration Ecology, Bridestown, Kildinan, T56 P499 Co. Cork, Ireland; ecrisol@coexphal.es; 2 Escuela de Agronomía, Facultad de Ciencias Agrarias y Forestales, Universidad Católica del Maule, Casilla 7-D, Curicó 3349001, Chile; emundaca@gmail.com; 3 Centre for Pesticide Suicide Prevention, University/BHF Centre for Cardiovascular Science, University of Edinburgh, Edinburgh EH16 4TJ, UK; 4 International Rice Research Institute, Makati, Manila 1226, Metro Manila, Philippines; 5 School of Environmental Science and Management, University of the Philippines, Los Baños 4030, Laguna, Philippines; 6 Association of Fruit and Vegetable Growers of Almeria (COEXPHAL), Carretera de Ronda 11, 04004 Almeria, Spain; 7 Department of Zoology, Ecology and Plant Science, University College Cork, Butler Building, Distillery Fields, North Mall, T23 N73K Cork, Ireland

**Keywords:** brown planthopper, herbivory tolerance, heterosis, host plant resistance, leaffolders, plant physiology, rice herbivores, stemborers, whitebacked planthopper, yields

## Abstract

**Simple Summary:**

Hybrid rice is produced by crossing a male sterile rice line (the A line) with a pollen doner that restores fertility (the restorer line). The consequential outbreeding can result in plant growth and yields that are above the average of the parental lines (a condition called heterosis). This gives rise to a normally higher productivity of hybrids compared to pure-line (inbred) rice varieties. However, hybrid rice has also been associated with higher herbivore damage compared to inbreds. In particular, susceptibility to the whitebacked planthopper (*Sogatella furcifera*) has been linked to the A line in early generation hybrids. We tested whether hybrids had heterosis for resistance to the whitebacked planthopper, brown planthopper (*Nilaparvata lugens*) and yellow stemborer (*Scirpophaga incertulas*) in a series of greenhouse and field experiments. In the field plots, heterosis resulted in relatively high hybrid yields. There were few cases of heterosis for resistance to herbivores in the greenhouse or field and only one of eight A lines was associated with susceptibility to planthoppers. Susceptibility to the yellow stemborer was largely due to plant physiology: larger, fast-growing and late-maturing varieties, including hybrids, were more vulnerable to stemborers. Therefore, susceptibility to stemborers was often a consequence of heterosis for plant biomass. We make a series of recommendations to improve screening and breeding for resistance to herbivores in hybrid rice.

**Abstract:**

Three-line hybrid rice is produced by crossing male sterile (A line) rice with a fertility-restorer (R line). Fertile lines (B lines) are also required to maintain A line seed for breeding programs. We used a range of hybrids and their parental lines to assess the frequency and nature of heterosis for resistance to the whitebacked planthopper (*Sogatella furcifera*), brown planthopper (*Nilaparvata lugens*) and yellow stemborer (*Scirpophaga incertulas*). Heterosis is defined as trait improvement above the average of the parental lines as a result of outbreeding. Based on the results from a greenhouse study that challenged hybrids and their parental lines with each herbivore species, we found that susceptibility to planthoppers was associated with one of the eight A lines tested, but resistance was improved by crossing with a relatively resistant restorer. Higher frequencies of heterosis for susceptibility in comparisons between hybrids and their B lines suggest that susceptibility was not related to the cytoplasmic genomes of the associated sterile A lines. Furthermore, because none of the parental lines possessed currently effective resistance genes, improved resistance against planthoppers was probably due to quantitative resistance. In a related field trial, hybrids had generally higher yields than their fertile parents and often produced larger grain; however, they were often more susceptible to stemborers, leaffolders (*Cnaphalocrocis medinalis*) and other caterpillars (*Rivula atimeta*). This was largely a consequence of hybrid heterosis for plant biomass and was strongly affected by crop duration. We make a series of recommendations to improve hybrid breeding to reduce the risks of herbivore damage.

## 1. Introduction

Rice is the main stable food for over a third of the world’s population [1,2]. One strategy to increase rice productivity has been to improve crop yields through hybrid rice breeding systems [3,4]. Hybrid rice varieties are distinct from pure-line (also referred to as inbred) varieties in that they result from crosses between a male-sterile plant (the female parent) and a pollen doner (the male parent or fertility restorer). Hybrid rice was first developed in China in the 1970s after the discovery of rice with male sterility (known as wild abortive cytoplasmic male sterility [WA-CMS]). Sterility allows rice breeders to better manage seeds and provides a mechanism to commercialize elite varieties. Hybrid rice varieties tend to have higher yields than inbred varieties (ca. 10% higher), and, where carefully selected, can have superior grain quality (e.g., lower chalk content: [3,4]). The higher yields are attributed to heterosis for plant growth (i.e., biomass accumulation)—sometimes referred to as ‘hybrid vigor’—and resource use efficiency. Heterosis is the result of outbreeding and is defined as a condition of traits where the hybrid’s performance exceeds that of the average performance of its parental lines. For some traits, the hybrid may surpass the performance of both parents; this is known as heterobeltiosis [5].

Since its initial development, hybrid rice has been widely adopted and is now extensively grown in some parts of Asia, and particularly in China [4,6,7]. During the first decades of largescale hybrid rice adoption, a number of problems related to rice pests and diseases became apparent. This included increased outbreaks of planthoppers (e.g., the whitebacked planthopper, *Sogatella furcifera* Horváth [WBPH]: [8,9], and the brown planthopper, *Nilaparvata lugens* (Stål) [BPH]: [10]) and stemborers (e.g., the striped stemborer, *Chilo suppressalis* (Walker) [SSB] and the yellow stemborer, *Scirpophaga incertulas* (Walker) [YSB]: [11,12,13]). A review by Horgan and Crisol (2013) [14] investigated these issues and identified a range of hypotheses and supporting studies to determine possible underlying mechanisms. The authors suggested that a limited availability and low genetic diversity of CMS lines and a lack of resistance to sap-sucking insects among parental lines was associated with relatively high planthopper densities in hybrid rice—this included CMS-associated susceptibility to WBPH [8,15]. 

To counter some of the pest-related problems, researchers have aimed to increase hybrid genetic diversity by selecting restorer lines that are increasingly genetically distant from the female parents. A greater genetic distance between parents also enhances heterosis [7,16,17,18]. Furthermore, greater diversity among rice varieties restricts the movement of pests and diseases between fields and provides a barrier to pest infestations [19,20]. However, not all pollen doners are capable of restoring fertility to the CMS lines [5,16]. Therefore, researchers have also aimed to incorporate resistance against planthoppers into high-yielding hybrid lines through marker-assisted breeding [21,22,23,24,25,26]. For stemborers and other chewing insects, researchers have mainly focused on incorporating transgenic resistance to parental lines, particular using *Bacillus thuringiensis* derived *Cry* genes [27,28,29,30]. These approaches are limited because only a few resistance genes are broadly effective against planthoppers [31] and because planthopper and stemborer populations can adapt to rice varieties with conventional and transgenic resistance [32,33,34]. In light of these constraints, hybrid breeders will need to further increase the genetic diversity of hybrid varieties, avoid herbivore-susceptible parental lines and, in particular, promote specific combinations of parental lines that achieve heterobeltiosis for herbivore resistance. Despite the need to improve hybrid resistance to herbivores, we know of no study that has examined whether heterosis for grain yields can be achieved while also maintaining or increasing varietal resistance to key rice pests.

In this study, we examine aspects of hybrid heterosis for resistance to planthoppers and stemborers using a range of hybrids from the three-line hybrid rice breeding program at the International Rice Research Institute (IRRI) in the Philippines. Our hypotheses were as follows: (1) hybrids are more susceptible to BPH and WBPH than their respective restorer lines because of a noted higher susceptibility of the CMS lines (possibly linked to cytoplasmic-associated susceptibility to WBPH [8,10,15,35]) and (2) hybrids are more susceptible to stemborers due to their faster growth rates and greater biomasses compared to parental lines (i.e., susceptibility to stemborers is a consequence of heterosis) [11,14,36]. We therefore conducted a range of greenhouse studies with up to 15 hybrid lines and their respective parental lines to compare BPH, WBPH and YSB responses to the lines and assess the frequency of heterosis and heterobeltiosis for antibiosis and antixenosis resistance. We further assessed the growth and yields of hybrids and their respective parental lines in a field trial and related plant anatomy to the occurrence of key planthopper and stemborer pests. Based on our results, we make recommendations for achieving durable resistance to herbivores through hybrid-breeding systems, including the proper selection of screening methods.

## 2. Materials and Methods

### 2.1. Plant Materials

We used fifteen hybrid rice varieties and their associated parental lines in our main greenhouse experiment. We then used a subset of eight varieties in our field trials. The lines were arbitrarily selected from the IRRI breeding program with no a priori considerations except that sufficient seed was available for experiments. The 3-line hybrid system requires three parental lines. These are the CMS line (also called the A line), the maintainer line (also called the B line) and the restorer line (the R line). Hybrid seed is produced by crossing the A line and restorer line. The B line is required to maintain A line seed. The B line has the same nuclear genome as the A line, but has a distinct cytoplasmic genome that permits the production of viable pollen [5,37]. The restorer lines are more genetically diverse than the A and B lines and are selected based on their ability to combine with the A line to restore fertility [5,16]. Restorer lines will also have generally desirable agronomic traits, including high yields and good grain quality, but can also have anti-herbivore or anti-disease traits [5]. Among the 15 hybrids that were used in the experiments, four (IR80156H, IR81954H, IR82385H, and IR82363H) had unique parental lines; among the remaining hybrids, 10 included duplicated female lines (IR70369A/B × 4; IR68897A/B × 2; IR80156A/B × 2), and seven had duplicated male lines (IR73013-95-1-3-2R × 4; IR 60819-34-2R × 3). Therefore, in total, we used 40 separate rice lines in the experiments. From the 15 hybrid groups, we focused on 8 in our oviposition and field experiments, 3 of which shared the restorer line IR60819-34-2R (i.e., a total of 30 lines). This reduced number of lines was selected to optimize the experimental design by including the greatest diversity of available parental lines for a smaller number of hybrid varieties. Only one restorer line, IR46, has a major gene (*Bph1*) for BPH resistance; however, BPH populations throughout Asia have adapted to feed on rice with this gene [31]. 

We assessed all hybrids and parental lines for resistance to BPH, WBPH and YSB. The rice seed was initially sowed to saturated, homogenized paddy soil in plastic basins (25 × 30 × 50 cm, H × W × L) in a shaded greenhouse (greenhouse and cage conditions, i.e., light, temperature, and humidity are detailed in a paper by Crisol et al. (2013) [38]). After 10 days, the seedlings were carefully pulled from the basins and transplanted to #3 pots (9.5 × 12.5 cm, H × D: for antixenosis experiments—see below) or #6 pots (15 × 15 cm, H × D: for oviposition experiments—see below) with saturated paddy soil. Each pot was planted with a single seedling. The pots were placed in flooded trays to avoid heat stress and prevent the entry of ants. The pots were watered daily and received no fertilizer or pesticide treatments. 

### 2.2. Insect Herbivores

We used BPH and WBPH in our greenhouse experiments. These two planthoppers are regarded as the most damaging hemipteran species of rice throughout Asia [39]. Winged adult planthoppers normally attack early-stage rice plants (tillering stage) where they feed and lay eggs. The nymphs pass through five instars with up to three generations per crop. Heavy infestations produce patches (known as hopperburn) of dead plants in rice fields [39]. Planthoppers of both species were from colonies initiated five years prior to conducting the experiments, each with >500 individuals collected using sweep nets in rice paddies from Laguna, Philippines. The colonies each had periodic introgressions of wild-caught planthoppers over the five years. The colonies were maintained in wire-mesh cages of 120 × 60 × 60 cm (H × W × L) in a shaded greenhouse. Planthoppers were continuously fed on a highly susceptible rice variety (TN1) for >30 days after sowing (DAS), with feeding plants changed every two weeks. The Laguna BPH population is highly virulent against rice varieties with the *Bph1*, *bph2*, *bph4*, *Bph18*, *BPH25*, and *BPH26* genes. The Laguna WBPH population is virulent against the *Wbph2*, *Wbph3*, *wbph4*, *Wbph6*, *WbphM1,* and *WbphM2* genes [31].

We also used YSB in our greenhouse experiments. This species is the main stemborer pest of rice in tropical Asia [40]. Stemborers attack rice at the seedling or tillering stages when adults deposit egg masses on the rice foliage. After emergence, the neonates migrate to the base of the rice plant and tunnel into the tillers where they feed and develop. There can be up to two generations of stemborer per crop [40]. YSB is difficult to maintain in laboratory colonies; therefore, we collected adults from rice fields in Laguna Province, 3–5 days before they were required for the experiments. The adult moths were collected before dusk using sweep nets. Adults were used directly in the oviposition experiments (see below). Where larvae were required for experiments, the recently-captured adults were placed in plastic cages (100 × 50 × 50 cm: H × W × L) with >30 DAS TN1 and allowed to mate and lay eggs. Egg masses were collected from the cages and placed in plastic Eppendorf tubes until the neonate larvae emerged. Neonates were used in experiments within 1 h of egg hatch. 

During the field experiment, we recorded Hemiptera sap-sucking insects (all planthoppers and leafhoppers), and Lepidoptera leaf-chewing and stem-boring insects. We grouped four species of leafhoppers together as vectors of tungro virus; these were *Nephotettix virescens* Distant, *Nephotettix nigropictus* (Stål), *Nephotettix malayanus* Ishihara and Kawaze, and *Recilia dorsalis* (Motchulsky). We also noted the occurrence and damage caused by rice leaffolders (mainly *Cnaphalocrocis medinalis* Guénee) and green hairy caterpillars (*Rivula atimeta* Swinhoe) and the occurrence of the pink stemborer (*Sesamia inferens* Walker [PSB]) We did not quantify other herbivores or natural enemies in the plots. 

### 2.3. Heterosis for Resistance in Greenhouse Experiments

#### 2.3.1. Planthopper Responses to Antibiosis of Hybrid and Parental Lines

At 25 DAS, plants in #6 pots were covered with an acetate cage (123 × 12 cm, H × D) with a nylon mesh window (23 × 15 cm: H × W) and nylon top. The pots were arranged as a replicated randomized block design with four replicated blocks. Each block was located in a separate greenhouse compartment (separated by screened partitions). Each block consisted of 80 pots (40 lines × 2 herbivore treatments [BPH and WBPH]) randomly positioned in each compartment. The entire experiment consisted of 320 pots. The plants were infested at 30 DAS with either two recently-emerged, gravid female BPH, or two recently-emerged, gravid female WBPH. The females were collected from the colonies using a suction pooter and inserted into the cages through a slit in the acetate. 

The planthoppers were allowed to feed and lay eggs, and the nymphs were allowed to develop. At 20 days after infestation, all planthoppers were removed from the plants using a vacuum sampler passed through the top of the cage (i.e., removing the top mesh). By 20 days, some of the cages already included a second generation of planthoppers. The collected insects were placed in test tubes and dried in a forced draught oven at 60 °C for one week. After drying, the planthoppers were weighed (total weight per plant) and planthoppers at each developmental stage were counted. We also noted the numbers of brachypterous adults and the proportion of adults that were female. 

#### 2.3.2. Planthopper Oviposition Responses to Hybrid and Parental Lines

Antixenosis resistance was assessed for eight of the hybrid groups (see supplementary information). At 15 DAS, the plants were placed in acetate cages (60 × 50 × 50: H × W × L). Each cage had four pots that included a hybrid line and its associated male and female parental lines—including the B lines. The plants were positioned in a square configuration without touching each other or the cage walls. 

Cages corresponding to each hybrid line were used in the experiment and were replicated on six greenhouse benches, each in a different greenhouse compartment. Each bench had 16 cages (one for each hybrid variety and planthopper species) in a random arrangement (total = 96 cages). When the plants reached 20 DAS, the cages were infested with either 4 gravid, female BPH or 4 gravid, female WBPH. To infest the cages, the planthoppers were collected from the colonies using a suction pooter and passed through a slit in the side of each acetate cage. The planthoppers were allowed to lay eggs for 7 days, after which the adults were collected and the rice plants were destructively sampled. 

The plants were sampled by carefully washing the soil from the roots and wrapping the plants in moistened tissue paper. The plants were then placed inside plastic pouches in a refrigerator (4 °C) until dissection. The plants were dissected under a stereo microscope (×10 magnification) to count the egg clusters and eggs. Egg counting was completed within 5 days of sampling. After the eggs had been counted, the plants were placed in individual paper bags and dried in a forced draught oven at 60 °C for one week. The dried plants were weighed using a digital balance.

#### 2.3.3. Stemborer Responses to Antibiosis of Hybrid and Parental Lines

At 25 DAS, plants were covered with an acetate cage (123 × 12 cm, H × D) with a nylon mesh window (23 × 15 cm: H × W) and nylon top. The pots were arranged as a replicated randomized block design with 6 replicated blocks. Each block was located in a separate greenhouse compartment. Each block consisted of 40 pots (i.e., 40 varieties) with the entire experiment consisting of 240 pots. 

To infest the plants, we placed six recently emerged neonate YSB directly onto the plant foliage using a fine paintbrush. The stemborers were allowed to develop until adult moths were noted in the cages. The adults were then removed daily until no new adults emerged. The time of adult emergence was recorded as the time to develop from neonate to adult. The sex of each adult was determined and the adults were individually weighed. 

#### 2.3.4. Stemborer Oviposition Responses to Hybrid and Parental Lines

Antixenosis resistance to YSB was assessed for eight of the hybrid groups (see supplementary information). At 15 DAS, the plants were placed in acetate cages (60 × 50 × 50: H × W × L) using the configuration explained in Section 2.3.2. The cages, corresponding to each of the eight hybrid lines were replicated on six greenhouse benches, each in a different greenhouse compartment (8 cages × 6 replicated compartments = 48 cages). The cages were arranged randomly on each bench. 

When plants reached 20 DAS, the cages were infested with 2 gravid female YSB. To infest the cages, the adult moths were passed through a slit in the side of each cage using a collecting tube. The moths were allowed to lay eggs for 7 days, after which they were removed from the cages and the egg masses were counted. The egg masses were collected in Eppendorf tubes and examined under a stereo microscope (×10 magnification) to count the numbers of eggs per mass. The rice plants were destructively sampled, placed in individual paper bags, and dried in a forced draught oven at 60 °C for one week before weighing.

### 2.4. Field Screening of Hybrid and Parental Lines

Field screening was carried out at the IRRI Experimental Station in Los Baños, Luzon Island, Philippines (14°11′ N, 121°15′ E). Luzon has a tropical maritime climate with a wet season from May to November (average monthly precipitation during the wet season = 120–400 mm, average monthly temperature = 26–29 °C). Field screening was conducted only during the wet season. The field site had high clay soils (clay content ca 60%) with ca. 3% organic carbon. Flooded rice had been grown at the site for several decades. Further details of the field experiment are presented by Horgan et al. (2016) [36].

Plants from each of 30 accessions (corresponding to eight hybrid varieties) were sowed to paddy soil in a greenhouse and transplanted at 21 DAS (21 DAS = 0 days after transplanting [DAT]) to field plots of 10 × 10 plants, with a plant spacing of 25 × 25 cm. Each line had five replicated plots (N = 5) laid out in a randomized block design. However, there were two sets of plots for IR60819-34-2R corresponding to two separate batches of seed from the breeding program that were used to generate the associated hybrids, and sufficient seed was available for only three replicated plots of IR68897B. Plants received N-P-K fertilizer (100-40-2) equivalent to 150 kg of ammonium sulphate per hectare. One third of the fertilizer was applied on the day prior to transplanting, one third during tillering, and one third at panicle initiation. Fields were flooded until before harvest. Plots received no pesticide treatments. 

#### 2.4.1. Biomass Accumulation and Yields of Hybrid and Inbred Lines in Field Plots

Each accession was monitored for biomass accumulation and yield by destructively sampling plants (two per line per block, cut at the base, above the roots) at two-week intervals and until final grain maturity (a total of 30 × 5 + 1 × 3 = 153 samples per time point). Plants were randomly selected from the plots using a random number generator and numbered hill positions. Prior to destructive sampling, we estimated relative chlorophyll content from the flag leaves of the same plants using a Minolta SPAD 502 chlorophyll meter (Konica, Tokyo, Japan). Plant samples were divided into vegetative (shoots) and reproductive parts (panicles and grain), were dried in a forced draught oven at 60 °C until a constant weight, and weighed on a digital balance. The number of tillers was also recorded. When ≥85% of the total panicles in each plot were mature and grain filling appeared complete, grain from an area of 1.25 m^2^ (=25 hills) was harvested from each plot to estimate final crop yields.

#### 2.4.2. Monitoring of Herbivores and Damage in Field Plots

Planthoppers and leafhoppers were sampled at 21, 56 and 84 DAT using a modified BlowVac suction sampler (Husqvarna, Stockholm, Sweden). Plants were randomly selected as described above (one per plot, a total of 153 samples per time point). Prior to sampling, a plastic cage was quickly placed over each plant. All insects on the plant—inside the cage—were then collected by thoroughly vacuuming the plant. Samples were stored in 90% ethanol, and leafhopper and planthopper species were separated, identified and counted. 

Ten rice plants in one central row per plot were carefully searched for stemborer egg masses at 56 DAT. Plants were destructively sampled for stemborers and other defoliators at 56 and 84 days after transplanting (DAT) and again at the time of harvest (date varied based on grain maturity—see below). The plants (one randomly selected plant per plot per sample period, a total of 153 samples per time point) were quickly pulled from the soil and placed in individual plastic sleeves. The sleeves were placed in a refrigerator at 4 °C until the plants were examined (within one week of collection). Plants were examined for insect damage and were dissected to collect any caterpillars. The plants were then dried at 60 °C and the total above-ground dry weight recorded together with the number of tillers. The following data were recorded for each tiller per collected plant: tiller dry weight, number and dry weight of leaves per tiller (i.e., leafiness), number and species of stemborers, number of leaffolders, number of green hairy caterpillars, proportion of tillers with deadheart, and proportion of tillers with whitehead. 

### 2.5. Data Analyses

This study mainly addresses the frequency of heterosis and heterobeltiosis for resistance to planthoppers and stemborers, as well as for plant growth and yields. To identify heterosis, hybrids were compared with their respective parental lines. This required a large number of separate analyses. To convey the main results from these analyses and avoid extensive reporting, we have included the full results as a series of supplementary tables and a supplementary figure. The main results are summarized in the body of the text with a focus on the different hybrid lines.

Fitness parameters (i.e., proportions of brachypterous planthopper adults, proportion of adults that were female, total number of individuals per plant, and total dry weight of insects at the end of the experiment) for BPH, WBPH and YSB in the greenhouse experiment were analyzed using univariate General Linear Models (GLM). Data on the numbers of YSB that were male or female and the relative development times and weights of male and female YSB were analyzed using univariate GLM with two main factors (accession and sex). Planthopper development (relative proportions at each development stage) was analyzed using multivariate GLM. Analyses were initially conducted with hybrids and their respective parental lines to identify cases of heterosis for resistance (i.e., where herbivore fitness parameters are significantly lower for hybrids compared to one parental line), heterobeltiosis for resistance (i.e., where herbivore fitness parameters are significantly lower for hybrids compared to both parental lines—including either the A line or B line), and heterobeltiosis for susceptibility (i.e., where herbivore fitness parameters are significantly higher for hybrids compared to both parental lines—including either the A line or B line). The same analyses (univariate or multivariate GLMs) were also conducted with the hybrids alone to determine whether heterosis was a necessary condition to achieve relatively high hybrid resistance. Proportional data was arcsine transformed and insect numbers were ranked before analyses. Replicated block (1–6) was initially included in the analyses but was removed because there was no significant effect. Pairwise comparisons were made using Tukey’s LSD tests. Oviposition data were ranked within replicated cages. The numbers of egg masses (YSB) or egg clusters (BPH and WBPH) and the numbers of eggs per plant were analyzed using univariate GLMs with tiller number and/or plant biomass as covariates. The analyses were repeated for the proportions (arcsine transformed) of the total numbers of eggs in each arena that were laid on each plant type. The biomass and tiller numbers of plants were also analyzed for the experiments using univariate GLMs. Pairwise comparisons were made using Tukey’s LSD tests.

Growth parameters (i.e., number of tillers, total plant dry weight, and SPAD values) for the eight hybrids used in the field experiment and their respective parental lines were analyzed using repeated measures GLM. Yield parameters for individual plants (i.e., number of reproductive tillers, number of panicles, weight of rachis, weight of reproductive tissues, number of filled grain, weight of filled grain, 1000-grain weight and the proportion of grain that was unfilled) and yield data from the 1.25 m^2^ (5 × 5 plant) crop cuts (per hectare yield, production of unfilled grain, 1000-grain weight) were analyzed using univariate GLM. The analyses were initially conducted for each hybrid and the respective parental lines and again for the hybrid lines alone. Furthermore, the analyses were repeated with the A lines included and excluded to draw attention to the unique parameters associated with male sterile plants (i.e., A lines included) and to draw meaningful comparisons between hybrids and their fertile parental lines to assess heterosis and heterobeltiosis (i.e., A lines excluded).

The occurrences of BPH, WBPH and other planthoppers and leafhoppers in the field plots were analyzed using repeated measures GLMs. The numbers of tillers per plant, plant dry weight, and SPAD values were initially included in the models as covariates but were removed where they had no significant effects. The numbers of leaf chewers and stemborers per plant were analyzed using multiple GLMs (separate analyses for chewers and stemborers). The covariates number of tillers, plant weight, and leafiness (i.e., the weight of leaves per tiller) were initially included in all models but were removed where they had no significant effects. Covariate values were directly associated with the sampled plants and were recorded at the time of plant dissection. The incidence of deadheart and whitehead and the number of YSB egg masses in the plots were analyzed using univariate GLMs; for egg masses, the covariates number of tillers, plant weight, and SPAD values were initially included in the model but removed where they had no significant effects. The numbers of leaf-chewers, stemborers, deadhearts, whiteheads and egg masses were ranked before analyses. Post hoc comparisons were conducted using Tukey’s LSD. All analyses of herbivores were conducted for hybrids and their respective parental lines to identify cases of heterosis and heterobeltiosis and again for hybrids only. We used multiple linear regression with backward elimination to determine the best predictors of BPH, WBPH, and YSB occurrence in the field plots. The analyses included all rice growth parameters recorded from the field plots at the time of maximum herbivore occurrence (i.e., 84 DAT and/or at harvest time), crop duration (i.e., the time from transplanting to grain harvest), and the occurrence of similar herbivores (e.g., for BPH, this included WBPH and other planthoppers; for YSB, this included other stemborers). Plant growth and other parameters were entered into the models irrespective of plant type and included all A lines. Residuals were plotted following all parametric analyses and were found to be normal and homogenous.

## 3. Results

### 3.1. Heterosis and Heterobeltiosis for Resistance in Greenhouse Experiments

#### 3.1.1. BPH Resistance

Three hybrid varieties (IR80228H, IR86167H, and IR82385H) were significantly more resistant to BPH than IR80814H (Tables S1 and S2). The most susceptible hybrid was bred using IR70369A as the female parent. This parental line was also associated with generally higher numbers of BPH and/or a greater final biomass of BPH in four other hybrids in the greenhouse experiments (IR81954H, IR81955H, IR81958H, and IR80228H: Table S2), suggesting that susceptibility was associated with this CMS line. However, crossing IR70369A with IR73885-1-4-3-2-1-6R produced one of the most resistant hybrid varieties (IR80228H) without significant heterosis or heterobeltosis, thereby indicating a strong association between resistance and the restorer line. In two cases (IR80814H and IR81954H), crossing resulted in heterobeltiosis for susceptibility to BPH (based on final numbers or weights of BPH); in a further case (IR81955H), crossing resulted in heterobeltiosis for susceptibility based on the proportions of early instars in the samples (i.e., a second generation of BPH: Table S1). Among the lines showing heterobeltiosis for resistance, only IR82363H had statistically significantly lower numbers of individuals than the parental lines; heterobeltiosis for resistance in IR82391H and IR84714H was based on significantly delayed BPH development; however, the final BPH numbers and biomass were also generally low in these hybrids compared to their respective parents (albeit not significantly so: Table S1).

**Figure 1:**
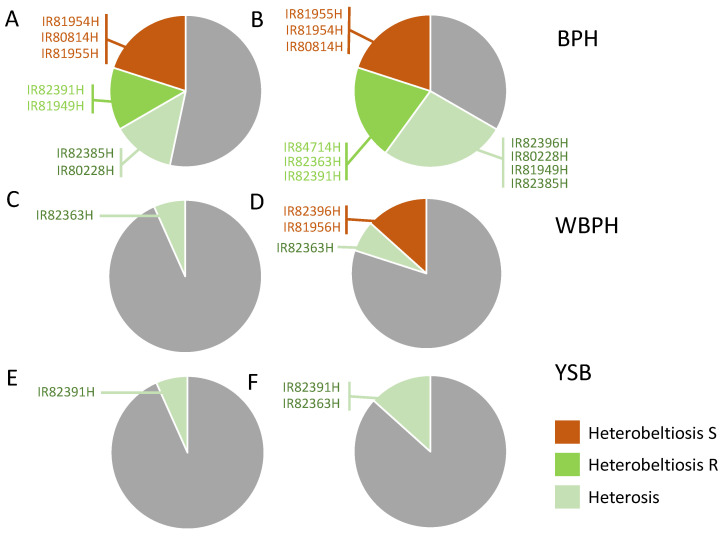
Frequency of heterosis and heterobeltiosis for resistance/susceptibility to (**A**,**B**) BPH, (**C**,**D**) WBPH and (**E**,**F**) YSB among 15 hybrid rice lines from the IRRI breeding program. Heterosis and heterobeltiosis are indicated based on statistical differences in any response (i.e., development time, number of individuals, herbivore weights, etc.) between hybrids and (**A**,**C**,**E**) the A lines or (**B**,**D**,**F**) B lines. Light green portions of each pie chart indicate heterosis, dark green indicates heterobeltiosis resulting in improved resistance, and orange indicates heterobeltiosis resulting in increased susceptibility. Grey portions indicate no significant differences between plant types. Hybrid lines are listed; for full details, including comparisons of herbivore responses on lines related to each hybrid, see Tables S1 and S2 (**A**,**B**), Tables S4 and S5 (**C**,**D**), and Tables S6 and S7 (**E**,**F**).

Overall, with respect to the A lines, 27% of cases demonstrated heterosis or heterobeltiosis for resistance, with 20% showing heterobeltiosis for susceptibility (Figure 1). Meanwhile, with respect to the B lines, 47% of cases demonstrated heterosis or heterobeltiosis for resistance and 20% heterobeltiosis for susceptibility (Figure 1).

BPH oviposition was unaffected by plant type in six of the eight three-line hybrid groups tested. For the IR82391H group, females laid more clusters and eggs in the hybrid and A line than in the R line (i.e., heterosis). For the IR84714H group, more eggs were laid in the A line than in the hybrid line; the covariate ‘plant biomass’ also affected the BPH cluster numbers in the IR84714H group (i.e., more clusters in larger plants) (Table S3 and Figure S1). Therefore, we consider that only 25% (two in eight) of the cases showed heterosis for antixenosis resistance to BPH, with susceptibility associated with the IR79156A line.

#### 3.1.2. WBPH Resistance

A second generation of nymphs on IR82396H plants by the end of the experiment (see proportions of 1st and 2nd instars in Table S4) contributed to a higher number of WBPH and a relatively high biomass of WBPH on this hybrid. This was the result of heterobeltiosis for susceptibility, as observed for development stages (Table S4). There was also an indication of heterobeltiosis for susceptibility in IR81956H (Table S4), but resistance in the hybrid was not significantly different from other hybrids (Table S5). There were no significant differences among WBPH responses to the 15 hybrid varieties (Table S5). Overall, there was only one case (7%) of heterosis (IR82363H) for resistance and two cases (13%) of herterobeltiosis for antibiosis susceptibility to WBPH (Figure 1) all of which were based on differences in planthopper development on the plants (i.e., proportions at different instars: Table S4). WBPH oviposition was not affected by plant type in seven of the eight 3-line hybrid groups; and there was no case of heterosis for antixenosis resistance among any of the hybrids (Table S3, Figure S1). 

#### 3.1.3. YSB Resistance

Total YSB numbers and/or biomass were higher in the hybrids than in the other plant types for nine of fifteen hybrid groups (Table S6). There were no differences in susceptibility across hybrids (Table S7) and only two cases of heterosis (IR82363H and IR82391H) and no case of heterobeltiosis, as indicated by statistically significant differences in stemborer responses to hybrids and one or both parental lines (Table S7, Figure 1). Plant type affected oviposition in two cases (IR82391H and IR84714H), but there was only one case of heterosis for oviposition (IR84714H) (Table S8, Figure S1).

### 3.2. Plant Development and Herbivore Densities in Field Plots 

#### 3.2.1. Plant Biomass and Yields

**Figure 2:**
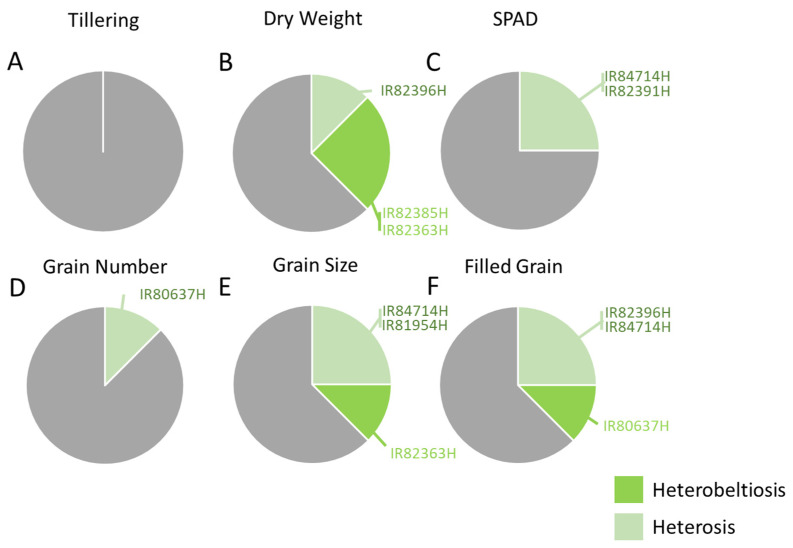
Frequency of heterosis and heterobeltiosis for (**A**) plant tillering, (**B**) dry weight, (**C**) SPAD values, (**D**) the number of filled grain produced, (**E**) the size of grain (i.e., 1000-grain weight), and (**F**) the proportions of grain that were filled among eight hybrid rice lines from the IRRI breeding program. Heterosis and heterobeltiosis are indicated based on statistical differences between hybrids and their relevant B and R lines. Light green portions of each pie chart indicate heterosis; dark green indicates heterobeltiosis resulting in improved plant growth or yields in field plots. Grey portions indicate no significant differences between plant types. Hybrid lines are listed; for full details see Tables S9–S12.

Full details of the plant growth parameters from the field experiment are presented in Table S9. We focused on three aspects of plant growth or anatomy as particularly relevant to herbivory; these are the tiller number, plant dry weight (i.e., total biomass) and SPAD values. There were no differences between hybrid varieties regarding tiller numbers during six sampling dates and no indication of heterosis for tillering among the hybrids (i.e., based on comparisons with relevant parental lines: Table S10, Figure 2). IR82396H showed heterosis for plant biomass; IR82385H and IR82363H were among the largest plants during the field trials (Table S10); both demonstrated heterobeltiosis for biomass (Table S9, Figure 2B). There were no differences between hybrids regarding the SPAD values. IR84714H and IR82391H showed heterosis for the SPAD values (Table S10, Figure 2C).

Details of per plant yields, including the biomass of reproductive structures and the numbers of grain and panicles are presented in Table S11. Across the eight hybrids, there were no significant differences in the numbers of reproductive tillers or panicles, rachis weight, or the number and percentage of grain that was unfilled (Table S12). IR82396H, IR81954H, IR80637H, and IR82363H had high per plant grain numbers (>2000 plant^−1^) and/or grain yields (>40 g plant^−1^) (Table S12). Higher yields were associated with heterosis for grain number in IR80637H, heterosis for grain size in IR81954H, and heterobeltiosis for grain size in IR82363H (Table S11, Figure 2D,E). Two of the higher-yielding plants also demonstrated heterosis (IR82396H) or heterobeltiosis (IR80637H) for the proportions of grain that were filled (Figure 2F).

Estimated hybrid yields based on crop cuts from each plot are presented in Table 1. There were no significant differences in the yields between hybrids and no differences in the production of unfilled grain across hybrids. IR81954H had larger grain than IR82396H and IR82391H (Table 1). The higher yields of hybrids compared to the B or R lines was attributed to heterosis in three hybrids (IR82396H, IR82391H and IR82385H) and heterobeltiosis in a further three hybrids (IR84714H, IR85471H and IR81954H) (Table 1 and Table S13).

#### 3.2.2. Abundance of Planthoppers and Leafhoppers in Field Plots

BPH and WBPH numbers were highest on rice sampled at 84 DAT (Table S14 and Table 2). The numbers of BPH and WBPH per sample differed significantly between the hybrid and parental lines in only one case (IR84714H), with consistently higher numbers of BPH on the IR80559A and B lines, and higher numbers of WBPH on the IR80559A line than on the restorer (IR60819-34-2R) (Table S14). This suggests that this A line in particular had a high field susceptibility to the planthoppers that was not transferred to the hybrid. Leafhopper vectors of rice viruses (i.e., *Nephotettix* spp. and *R. dorsalis*) were also more abundant during the 84 DAT sampling. There were two cases of significantly lower numbers of virus vectors on hybrids (IR82396H and IR81954H) compared to B lines (IR80156B and IR70369B, respectively) but not between the hybrids and the respective A lines (Table S14). There were no differences in the numbers of BPH, WBPH, virus vectors, or other Hemiptera across hybrids (Table 2).

The best predictors of BPH numbers in the samples were the occurrence of WBPH in the same sampled plots (slope = 0.618, t = 6.667, *p* < 0.001) and the average dry weights of the rice stems (slope = −1.988; t = −3.831, *p* < 0.001) (R^2^ = 0.691; F_2,31_ = 32.410, *p* < 0.001). The best predictors of WBPH numbers were the occurrence of BPH (slope = 0.840; t = 5.763, *p* < 0.001), the occurrence of other Hemiptera (slope = 5.998; t = 2.533, *p* = 0.017), and the average dry weights of the rice stems (slope = −2.196; t = 3.139, *p* = 0.004) (R^2^ = 0.682, F_3,31_ = 20.009, *p* < 0.001).

#### 3.2.3. Lepidopteran Herbivores in Field Plots

Leaffolder numbers were higher in IR85471H than IR82391H (Table 3), with significant positive effects of the covariates tiller number and plant height. There were six cases of significant plant-type effects on leaffolder numbers, but only three represented heterosis for resistance; for IR82396H and IR85471H, the hybrids had significantly more leaffolders than their respective B lines (but not different from the R lines), and for IR82391H, the hybrid had fewer leaffolders than the R line (Table S15). 

The hybrids had similar numbers of green hairy caterpillars, although the numbers differed between hybrids during the two sampling dates (Table 3). The covariates plant height and leafiness strongly affected the occurrence of these caterpillars. Comparisons of caterpillar numbers between lines indicated heterobeltiosis for susceptibility to the green hairy caterpillar in the hybrids IR81954H and IR82363H (Table S15 and Table 3).

There were no differences in the numbers of egg masses detected on hybrids at 56 DAT (F_7,31_ = 1.773, *p* > 0.05); however, the covariate SPAD value was associated with densities of egg masses (F_1,31_ = 6.835, *p* < 0.05: Table S15). There was no effect of hybrid line on the incidence of deadhearts (F_7,31_ = 1.053, *p* > 0.05: Table S15). Three stemborer species occurred in the field plots. The YSB was the most abundant species, with SSB and PSB occurring in relatively low numbers. The YSB densities significantly differed between hybrids at the time of harvest, with the highest numbers on IR82385H. Based on comparisons with the respective parental lines, this represented heterobeltiosis for susceptibility; however, the hybrids were sampled at least two weeks later than the parental lines based on grain maturity. The time of harvest was the best predictor of stemborer damage (R^2^ = 0.577, F_1,31_ = 40.936, *p* ≤ 0.001). Four other hybrids (IR82391H, IR84714H, IR85471H, and IR82363H) showed heterosis for YSB densities and/or whitehead damage (Table 3). 

## 4. Discussion

### 4.1. Resistance to Planthoppers and Leafhoppers

In our greenhouse experiment with 15 hybrid varieties and their parental lines, we noted that only one A line (IR70369A) was associated with susceptibility to BPH and WBPH in some resulting hybrids. Cohen et al. (2003) [37] found similarly low variability across BPH responses to hybrids and their parental lines using a different range of hybrids from the same breeding program. Susceptibility related to IR70369A was more consistent in our experiments with BPH than WBPH (i.e., other lines derived from IR70369A tended to also have high numbers or a high biomass of BPH, but not of WBPH: Tables S2 and S5). Cases of heterobeltiosis for susceptibility to both BPH and WBPH were more frequent when comparing planthopper fitness on hybrids and their related B lines, suggesting that increased susceptibility in these hybrids was not associated with the female cytoplasm. There is evidence that cytoplasm-linked susceptibility had been associated with high WBPH densities on hybrids from previous studies [8,9,15,35]; however, this potential problem with the WA-CMS lineage seems to have been resolved either because of the specific A lines used in our experiments or through the specific crosses that we examined. Indeed, there is evidence that crossing IR70369A with the restorer IR73885-1-4-3-2-1R produced a relatively resistant variety, despite a general susceptibility of IR70369A-derived hybrids when other restorers were used (Table S1). The availability of alternative A lines for three-line hybrid breeding has increased in recent decades [41,42,43], and, furthermore, two-line breeding systems based on inducing sterility through heat (i.e., thermosensitive genetic male sterile lines, TGMS) or photosensitivity (i.e., photosensitive genetic male sterile lines, PGMS) are now available [44,45,46], which can help avoid any susceptibilities that may be linked to specific female parents.

Cases of heterosis or heterobeltiosis for resistance were more frequent in our experiments with BPH than with WBPH (Figure 1A–D). Across hybrids, two of the most BPH resistant varieties (IR82396H and IR86167H) shared the parental line IR80156A, but IR86167H also shared the restorer line IR73013-95-1-3-2R with three other hybrids, of which none showed any particularly good resistance. This suggests that this female line—IR90156A—may be generally suitable in crosses to avoid BPH susceptibility; however, further possible hybrids that share this female parent should be examined for their resulting resistance to BPH to corroborate this finding. In any case, our greenhouse experiments indicated that heterobeltiosis for resistance in seedlings against BPH in particular, but WBPH also, can be achieved by carefully selecting both the female and male parents and that this resistance will likely be associated with nuclear genes (because of a low frequency of A line-derived susceptibility) and possibly quantitative resistance traits (because no currently effective resistance genes have been identified from any of the parental lines used in the experiments [47]).

We cannot explain which factors likely influenced observed resistance to BPH and WBPH in our greenhouse experiments. At the seedling stage, the hybrid varieties tended to be larger than the B lines and restorers (i.e., see Tables S3 and S8). Indeed, B line plants were often quite small even compared to their respective A lines at early seedling stages. Therefore, cases of heterobeltiosis for susceptibility could be due to a generally higher fitness of planthoppers on the larger and faster-growing hybrids [36]. This did not occur in three hybrids (IR80814H, IR81954H, and IR81955H) that showed heterobeltiosis for resistance, thereby further suggesting that quantitative resistance to planthoppers is achievable through the careful selection of parental lines. In our oviposition experiments, plant size affected one of two cases of heterosis for antixenosis resistance; suggesting that there was little effect of breeding on other factors, such as plant volatiles or silicon-based defenses [48,49,50,51,52,53,54] that are involved in antixenosis resistance. The experiments indicate the importance of separating physiologically-dependent susceptibility (e.g., related to size, growth rates, etc.) of rice lines from biochemical or other anatomical features, such as surface waxes or leaf hairs [48,55,56] that may be more closely associated with specific and heritable herbivore defenses. 

There were no apparent differences in the densities of BPH, WBPH, or other planthoppers in our field experiment. Furthermore, we detected only two cases of heterosis for resistance associated with the numbers of virus vectors on hybrid and parental lines. In both cases, the highest numbers of *N. virenscens* were associated with the A lines, including IR70369A—the line associated with susceptibility to BPH from the greenhouse study. This suggests that susceptibility to *N. virenscens* in some hybrids is associated with the female parents. In general, there was little relation between the greenhouse and field results (i.e., correlation between BPH numbers in the greenhouse and field: Spearman’s = −0.063, *p* = 0.932; correlation between WBPH in the greenhouse and field: Spearman’s = −0.046, *p* = 0.802). The lack of any correlations between the greenhouse and field results are probably due to the effects of density dependent predation and parasitism, which could obscure comparative fitness responses among planthoppers on different varieties [57,58,59]. Field experiments cannot therefore be recommended for detecting differences in quantitative resistance to planthoppers among hybrids and their parental lines because of the high resolution required to distinguish planthopper responses on the different plant types.

### 4.2. Resistance to Leaf Chewers and Stemborers

We compared responses to hybrids with responses to B lines and restorers (i.e., not the A lines) as a test of heterosis for resistance to stemborers because of the unusual physiology of the sterile lines. In general, stemborer fitness was greater on hybrids than on the other plant types (i.e., higher fitness on hybrids in nine of 15 cases in the greenhouse: Table S7); although the differences were seldom statistically significant (Figure 1E,F). In the oviposition experiment, egg laying was also often associated with larger hybrid plants (Figure S1). In contrast to planthoppers, the field experiment did reveal plant-type effects on YSB densities, with two cases of hybrid heterosis and one case of heterobeltiosis for susceptibility when compared against B lines and restorers. Furthermore, the cases of heterosis were consistent with the results from the greenhouse. However, these results were strongly affected by tillering, plant size and crop duration (see below). 

In the field, the A lines had profuse tillering and often attained a relatively large size compared to the fertile lines. Recent detailed studies of plant growth and stemborer occurrence in the Philippines has indicated that YSB—the predominant species occurring in our field plots—is associated with high tillering and relatively thick stems; and that much of the variability in stemborer damage to rice varieties can be explained by crop duration [40,60]. Rice varieties that are exposed for longer in the field tend to incur greater damage from stemborers, which is better regarded as crop ‘vulnerability’ and not ‘susceptibility’ [60]. This indicates that resistance to stemborers in hybrid rice is largely physiology-dependent and, therefore, heterosis for resistance is determined by heterosis for biomass accumulation and yields. This idea is supported by the results of our multiple regression analyses where the best predictor of stemborer occurrence and whitehead damage was crop duration, with SPAD values and stem dry weight also contributing to stemborer damage (R^2^ = 0.652). SPAD values were also associated with the occurrence of YSB egg masses across plots during early season sampling (Table S15). SPAD values indicate the relative greenness of plants and can be related to nutrient assimilation (particularly the assimilation of nitrogen) [61,62]. 

For leaffolders and the green hairy caterpillar, numbers were highest on hybrids compared to the other plant types in seven of eight cases. In these cases, unlike with stemborers, the leaf chewers were not more abundant on the A lines, indeed they often occurred at the lowest numbers on both the A lines and B lines (Table S15). The negative effect of tillering on leaffolders and the positive effect of leafiness (Table 3) on green hairy caterpillars suggest that the availability of leaf tissues strongly influenced these two species. Furthermore, our results indicate that in four of eight cases, hybrids had the greatest weights of leaf material compared to other plant types, but in only one case did any A line have more leaf material than it’s associated hybrid (Table S9). These results indicate that field resistance is more closely related to plant development and, therefore, physiologically-based susceptibility than to any biochemical or other direct defense traits. Rice varieties can have constitutive and induced defenses to leaf chewers and stemborers [60,63,64,65,66], but in general, these appear too weak to significantly reduce damage to below economic thresholds in poorly managed fields. 

### 4.3. Recommendations

Susceptibility can be avoided through the large-scale and systematic screening of rice lines for resistance during development, and the consequent elimination of highly susceptible lines [31]. However, breeders’ preferences for parental lines because of associated high yields or good grain quality can override decisions to eliminate susceptible parental lines from breeding programs. In such cases, as was shown with one restorer line in our study, the associated parental susceptibility can be overcome where crossing results in heterobeltiosis for resistance or where the restorer has good quantitative resistance. Breeders can also introduce resistance genes into breeding programs through suitable doners; however, we recommend that the final hybrids have further background quantitative resistance—as is the case with some successful rice megavarieties (i.e., IR64 [47,67,68]). The introgression of major resistance genes to plants with a good background, quantitative resistance, or tolerance is predicted to increase the durability of resistance and prevents yield losses after herbivores have adapted to specific resistance genes. 

Our results indicate that attaining quantitative resistance in hybrids against leaffolders, other leaf chewers, and stemborers is hardly practical. Indeed, susceptibility to Lepidoptera in hybrid rice is probably a consequence of heterosis for growth and biomass accumulation in most cases. This can be reduced by avoiding long-duration varieties, thereby reducing vulnerability [60]. However, even where varieties are selected to avoid specific stemborer-related susceptibilities, i.e., by reducing tillering to avoid YSB damage or reducing stem thickness to avoid SSB damage, other stemborers from the regional assemblage of species can become prevalent [40]. For this reason, calls have been made for greater attention to issues of rice tolerance to stemborers. Several authors have suggested that hybrid varieties are more tolerant than inbred varieties [36,69,70,71,72,73]. Tolerance is related to the plant’s capacity to respond to damage through increased assimilation of resources, increased tillering or the shifting of resources from damaged to non-damaged tissues [36,74,75]. 

## 5. Conclusions

Heterosis for biomass accumulation and yields can be achieved in hybrids without notable increases in hybrid susceptibility to planthoppers. Susceptibility to planthoppers is not greater for A lines than restorers, and the resulting hybrids are not generally susceptible to planthopper attack. Because of the fine resolution needed in comparisons between plant types and the impact of biotic factors on final planthopper communities in field trials, future hybrid rice breeding programs will need to conduct relatively detailed greenhouse bioassays to assess planthopper responses to hybrid rice breeding and to ensure that A line susceptibility to planthoppers is rectified by crossing with relatively resistant restorer lines. Heterosis for plant biomass can be associated with susceptibility to leaffolders and stemborers. Field trials are better suited than greenhouse experiments to evaluate hybrids and other plant types for their susceptibility, tolerance, and vulnerability to stemborers and defoliators. Long-duration varieties should be avoided for regions that are frequently affected by stemborers. Hybrids with equivalent high yields, but of relatively short duration are achievable. 

## Figures and Tables

**Table 1:** Estimated yields of eight hybrids based on harvested field plots. Cases of heterosis [ht] and heterobeltiosis [hb+] are indicated. See Table S13 for further details.

Accession	Harvest Time (DAT)	Yield (Tonnes Hectare^−1^) ^1^	Production of Unfilled Grain (Tonnes Hectare^−1^) ^1^	1000 Filled Grain Weight (mg) ^1^
IR82396H	98	6.43 (0.12) [ht]	0.67 (0.05)	21.12 (0.19) ^a^
IR82391H	93	5.73 (0.30) [ht]	0.85 (0.03)	20.90 (0.23) ^a^ [ht]
IR84714H	93	7.02 (0.10) [hb+]	0.55 (0.36)	22.67 (0.30) ^ab^
IR85471H	93	6.24 (0.18) [hb+]	0.70 (0.10)	22.25 (0.49) ^ab^
IR81954H	98	5.68 (0.20) [hb+]	0.83 (0.57)	25.25 (0.14) ^a^ [hb+]
IR80637H	98	5.20 (0.33)	0.71 (0.34)	22.47 (0.29) ^ab^
IR82385H	112	6.38 (0.10) [ht]	0.73 (0.43) [ht]	22.88 (0.78) ^ab^
IR82363H	93	6.30 (0.22)	0.70 (0.54)	23.50 (0.25) ^ab^ [ht]
F-Accession		1.414 ns	0.497 ns	2.520 *

^1^: Numbers are means (SEM) (N = 5); DF = 7,32; ns = *p* > 0.05, * = *p* ≤ 0.05; lowercase letters indicate homogenous hybrid groups (Tukey: *p* > 0.05); ht = heterosis, hb+ = heterosis for increased parameter values.

**Table 2:** The relative abundance of planthoppers and leafhoppers on eight hybrid rice varieties in field plots at three sampling times. See Table S14 for full details and comparisons between hybrid and parental lines.

Accession	Sampling Time (DAT)	BPH (Number Sample^−1^) ^1^	WBPH (Number Sample^−1^) ^1^	Virus Vectors (Number Sample^−1^) ^1^	Other Hemiptera (Number Sample^−1^) ^1^
IR82396H	21	0.20 (0.09)	2.80 (0.63)	3.40 (0.91)	0.00 (0.00)
	56	15.20 (2.38)	16.20 (2.99)	43.00 (8.57)	1.00 (0.45)
	84	0.80 (0.26)	3.20 (0.49)	9.60 (1.08) [ht]	0.40 (0.18)
IR82391H	21	0.40 (0.18)	3.60 (0.87)	2.80 (0.79)	0.00 (0.00)
	56	11.60 (2.19)	27.40 (4.53)	26.40 (4.57)	1.20 (0.38)
	84	2.40 (0.30)	4.00 (0.87)	13.80 (1.87)	0.60 (0.27)
IR84714H	21	1.00 (0.20)	5.60 (1.87)	2.80 (0.60)	0.00 (0.00)
	56	14.40 (2.55)	12.80 (2.51)	14.40 (2.55)	0.80 (0.27)
	84	0.80 (0.22)	8.40 (2.54)	7.00 (1.46)	0.40 (0.18)
IR85471H	21	1.00 (0.24)	4.20 (1.00)	2.40 (0.50)	0.20 (0.09)
	56	27.20 (7.65)	26.20 (6.37)	34.00 (9.92)	0.20 (6.09)
	84	0.80 (0.17)	7.00 (0.81)	8.20 (1.57)	0.40 (0.18)
IR81954H	21	2.40 (0.87)	7.80 (0.64)	2.40 (0.59)	0.00 (0.00)
	56	25.00 (3.97)	43.60 (7.79)	33.20 (6.35)	0.20 (0.09)
	84	1.20 (0.26)	7.00 (2.50)	3.80 (0.87) [ht]	0.00 (0.00)
IR80637H	21	0.40 (0.18)	5.40 (1.14)	2.60 (0.57)	0.00 (0.00)
	56	27.20 (3.70)	37.40 (5.80)	16.60 (3.97)	1.00 (0.38)
	84	0.60 (0.11)	1.60 (0.61)	2.80 (0.68)	0.40 (0.18)
IR82385H	21	0.20 (0.09)	6.20 (1.41)	3.20 (1.02)	0.20 (0.09)
	56	40.40 (5.70)	29.20 (3.73)	38.80 (8.01)	0.60 (0.27)
	84	1.40 (0.23)	6.60 (1.00)	7.80 (1.29)	1.00 (0.31)
IR82363H	21	1.00 (0.24)	6.00 (0.87)	6.60 (1.40)	0.00 (0.00)
	56	23.20 (3.37)	34.80 (5.01)	24.40 (4.47)	0.00 (0.00)
	84	1.40 (0.39)	3.20 (0.89)	9.20 (1.34)	0.20 (0.09)
F-Time		41.726 ***	35.708 ***	5.993 ***	27.674 ***
F Time × Accession		0.954 ns	0.907 ns	0.584 ns	0.672 ns
F-Accession		0.887 ns	0.810 ns	1.338 ns	0.748 ns

^1^: Numbers are means (SEM) (N = 5); DF = 2,64, 14,64 and 7,32 for Time, Time × Accession, and Accession, respectively; ns = *p* > 0.05, *** = *p* ≤ 0.005; ht = heterosis.

**Table 3:** The relative abundance of leaffolders (*C. medinalis*), green caterpillars (*R. atimeta*), and stemborers on eight hybrid rice varieties in field plots. The incidence of whiteheads in the plots is also presented. See Table S15 for full details and comparisons between hybrid and parental lines (excluding A lines).

Accession	Sampling Time (DAT)	*C. medinalis* (Number Plant^−1^) ^1,2,4^	*R. atimeta* (Number Plant^−1^) ^1,2,4^	PSB (Number Plant^−1^) ^1,3,4^	SSB (Number Plant^−1^) ^1,3,4^	YSB (Number Plant^−1^) ^1,3,4^	Whitehead (Proportion Plant^−1^) ^1,3,4^
IR82396H	84	0.89 (0.08)	0.67 (0.14)				
	98	0.55 (0.04) ^ab^ [ht]	0.22 (0.03)	0.60 (0.18)	0.40 (0.18)	1.60 (0.39) ^ab^	0.15 (0.02) ^ab^
IR82391H	84	0.50 (0.07)	0.52 (0.12)				
	93	0.30 (0.05) ^a^ [ht]	0.83 (0.12)	0.00 (0.00)	0.00 (0.00)	0.00 (0.00) ^a^ [ht]	0.01 (0.00) ^a^ [ht]
IR84714H	84	0.52 (0.04)	0.38 (0.06)				
	93	0.76 (0.10) ^ab^	0.84 (1.00)	0.00 (1.00)	0.00 (1.00)	0.00 (0.00) ^a^	0.00 (0.00) ^a^ [ht]
IR85471H	84	0.88 (0.08)	0.32 (0.06)				
	93	0.89 (0.04) ^b^ [ht]	0.69 (0.05)	0.20 (0.09)	0.00 (0.00)	0.40 (0.11) ^a^	0.04 (0.01) ^a^ [ht]
IR81954H	84	0.66 (0.11)	0.22 (1.06)				
	98	0.49 (0.14) ^ab^	0.45 (1.06) [hb−]	0.50 (0.14)	0.25(0.13)	3.50 (0.66) ^ab^	0.14 (0.02) ^ab^
IR80637H	84	0.61 (0.11)	0.23 (1.06)				
	98	0.62 (0.05) ^ab^	0.42 (0.08)	0.60 (0.18)	0.00 (0.00) ^5^	2.80 (0.52) ^ab^	0.26 (0.03) ^b^
IR82385H	84	0.63 (0.05)	0.55 (0.12)				
	112	0.39 (0.02) ^ab^	0.25 (0.05)	0.60 (0.11)	0.80 (0.17)	5.00 (0.73) ^b^ [hb−]	0.25 (0.02) ^b^ [hb−]
IR82363H	84	0.77 (0.06)	0.47 (0.00)				
	93	0.55 (0.05) ^ab^	0.70 (0.00) [hb−]	0.00 (0.00)	0.40 (0.18)	0.20 (0.09) ^a^ [ht]	0.03 (0.01) ^a^ [ht]
F-Time		0.105 ns	0.041 ns				
F-Time × Accession		0.630 ns	3.497 ***				
F-Accession		2.896 *	1.067 ns	1.370 ns	1.323 ns	4.539 ***	6.880 ***
F-Tillers		7.866 **	7.635 **				
F-Height		4.470 *	9.083 ***				
F-Leafiness		0.303 ns	15.369 ***				

^1^: Numbers are means (SEM) (N = 5); PSB = pink stemborer, SSB = striped stemborer, YSB = yellow stemborer; ^2^: DF = 1,59 for sampling time, 7,59 for accession and interaction effect, and 1,59 for covariates; ^3^: DF = 7,31; ^4^: ns = *p* > 0.05, * = *p* ≤ 0.05, ** = *p* ≤ 0.01, *** = *p* < 0.001; lowercase letters indicate homogenous hybrid groups (Tukey: *p* > 0.05); ht = heterosis, hb− = heterobeltiosis for susceptibility; ^5^: too few individuals were collected from hybrids and parental lines for analysis.

## Data Availability

The data presented in this study are available upon reasonable request from the corresponding author.

## Supplementary Materials

The following supporting information can be downloaded at https://www.mdpi.com/article/10.3390/insects15030164/s1, Table S1: Results from bioassays with BPH on 15 hybrid rice varieties and their female (A and B lines) and male (restorer/R lines) parents, Table S2: Responses by BPH to 15 hybrid rice varieties indicating potential sources of resistance/susceptibility based on heterosis or heterobeltiosis with parental lines, Table S3: Oviposition by BPH and WBPH during choice experiments with hybrids and associated parental lines, Figure S1: Proportion of eggs laid on hybrid rice and associated parental lines in a series of choice experiments, Table S4: Results from bioassays with WBPH on 15 hybrid rice varieties and their female (A and B lines) and male (restorer/R lines) parents, Table S5: Responses by WBPH to 15 hybrid rice varieties indicating potential sources of resistance/susceptibility based on heterosis or heterobeltiosis with parental lines, Table S6: Responses by YSB to 15 hybrid rice varieties and their female (A and B lines) and male (restorer lines) parents, Table S7: Responses by YSB to 15 hybrid rice varieties indicating potential sources of resistance/susceptibility based on heterosis or heterobeltiosis with parental lines, Table S8: Oviposition by YSB in choice experiments with hybrids and associated parental lines, Table S9: Plant growth parameters in field plots at IRRI, Table S10: Hybrid growth parameters in field plots at IRRI, Table S11: Plant reproductive parameters in field plots at IRRI, Table S12: Hybrid reproductive parameters in field plots at IRRI, Table S13: Yields of filled and unfilled grain from crop cuts in field plots at IRRI, Table S14: Results from BlowVac sampling of rice field plots at IRRI, Table S15: Results from egg mass sampling and dissections of rice plants sampled from rice field plots at IRRI indicating the occurrence of Lepidopteran defoliators and stemborers.
