# Supplementary material for: Heterosis for Resistance to Insect Herbivores in a 3-Line Hybrid Rice System
Source: Insects. 2024 Feb 28;15(3):164. doi: 10.3390/insects15030164 (PMC10970888; doi:10.3390/insects15030164)
Supplement: Supplementary file 1 [file insects-15-00164-s001.zip › insects-2839492-supplementary.pdf]

Supplementary Information

Table S1: Results from bioassays with BPH on 15 hybrid rice varieties and their female (A and B lines) and male (restorer/R lines) parents. Summarized information from this table is presented in Table S2 and Figure 1.

Accession	Development Stages[1]						Brachypterous (Proportion) [1]	Females (Proportion) [1]	Total Number of Individuals[1]	Total Dry Weight[1,2]
	1st instars	2nd instars	3rd instars	4th instars	5th instars	adults				
IR 82396 H	0.28 (0.06)b	0.22 (0.07)	0.21 (0.02)	0.20 (0.080	0.06 (0.03)	0.04 (0.03)	0.67 (0.33)	0.44 (0.29)	449.00 (161.78)ab	28.35 (6.21)
IR 80156 A	0.41 (0.05)b	0.17 (0.03)	0.17 (0.02)	0.12 (0.03)	0.09 (0.01)	0.03 (0.01)	0.90 (0.10)	0.08 (0.04)	333.25 (41.54)ab	72.28 (31.19)
IR 80156 B	0.06 (0.03)a	0.10 (0.05)	0.28 (0.10)	0.27 (0.05)	0.13 (0.06)	0.16 (0.15)	1.00 (0.00)	0.33 (0.33)	60.50 (35.89)a	7.32 (2.90)
IR 46 R	0.24 (0.03)ab	0.23 (0.02)	0.26 (0.01)	0.19 (0.04)	0.07 (0.02)	0.01 (0.01)	0.90 (0.10)	0.19 (0.12)	812.75 (192.85)b	57.81 (5.63)
F-Accession	10.800***	1.639ns	0.911ns	1.344ns	0.719ns	0.852ns	0.647ns (3,9)	0.523ns (9)	5.860*	3.239ns
IR 82391 H	0.36 (0.07)b	0.23 (0.04)	0.16 (0.03)	0.12 (0.03)	0.10 (0.02)	0.03 (0.01)	0.69 (0.20)	0.16 (0.06)	254.00 (47.22)	30.66 (6.28)
IR 79156 A	0.22 (0.03)ab	0.25 (0.06)	0.28 (0.02)	0.16 (0.04)	0.08 (0.03)	0.02 (0.01)	0.98 (0.02)	0.22 (0.15)	696.50 (167.12)	58.50 (16.11)
IR 79156 B	0.19 (0.08)ab	0.18 (0.05)	0.16 (0.02)	0.14 (0.02)	0.17 (0.05)	0.15 (0.14)	0.85 (0.15)	0.13 (0.10)	353.25 (189.30)	35.68 (5.28)
IR 60819-34-2	0.09 (0.04)a	0.15 (0.06)	0.32 (0.15)	0.15 (0.04)	0.14 (0.04)	0.15 (0.13)	1.00 (0.00)	0.24 (0.09)	698.00 (267.68)	58.58 (4.27)
F-Accession	3.472*	0.687ns	0.987ns	0.267ns	1.198ns	0.606ns	1.159ns	0.265ns	1.549ns	2.534ns
IR 84714 H	0.34 (0.08)b	0.23 (0.01)bc	0.17 (0.02)	0.14 (0.04)a	0.10 (0.04)a	0.02 (0.01)	1.00 (0.00)	0.19 (0.11)	330.25 (77.29)ab	33.47 (6.89)
IR 80559 A	0.06 (0.00)a	0.08 (0.02)a	0.22 (0.03)	0.28 (0.00)b	0.32 (0.06)b	0.04 (0.01)	1.00 (0.00)	0.08 (0.03)	306.00 (72.20)a	45.00 (5.13)
IR 80559 B	0.23 (0.06)ab	0.25 (0.02)c	0.31 (0.02)	0.14 (0.04)a	0.08 (0.01)a	0.00 (0.00)			755.50 (36.19)b	43.21 (5.46)
IR60819-34-2 R	0.16 (0.05)ab	0.17 (0.02)b	0.22 (0.07)	0.20 (0.03)ab	0.21 (0.05)ab	0.04 (0.02)	0.86 (0.07)	0.57 (0.21)	344.50 (184.87)ab	43.47 (11.94)
F-Accession	4.511*	21.935***	1.729ns	4.476*	6.201**	2.809ns	4.891* (2,7)	4.346ns (2,7)	3.957*	0.451ns
IR 85471 H	0.11 (0.05)	0.23 (0.05)	0.25 (0.06)	0.26 (0.03)	0.12 (0.02)	0.03 (0.01)	0.85 (0.10)	0.17 (0.10)ab	458.00 (170.76)	52.52 (8.08)
IR 80564 A	0.23 (0.06)	0.17 (0.01)	0.24 (0.00)	0.20 (0.04)	0.15 (0.04)	0.00 (0.00)	0.00 (0.00)	0.00 (0.00)a	317.50 (41.48)	24.23 (5.81)
IR 80564 B	0.15 (0.06)	0.24 (0.04)	0.22 (0.04)	0.23 (0.05)	0.14 (0.03)	0.01 (0.00)	0.75 (0.25)	0.00 (0.00)a	265.75 (54.44)	24.73 (7.99)
IR60819-34-2 R	0.16 (0.05)	0.17 (0.02)	0.22 (0.07)	0.20 (0.03)	0.21 (0.05)	0.04 (0.02)	0.86 (0.07)	0.57 (0.21)b	344.50 (184.87)	43.47 (11.94)
F-Accession	0.841ns	1.437ns	0.114ns	0.640ns	1.474ns	1.442ns	3.628ns (3,8)	5.363* (3,8)	0.388ns	2.593ns
IR 81954 H	0.17 (0.05)	0.21 (0.03)	0.38 (0.08)	0.15 (0.03)	0.08 (0.03)	0.00 (0.00)a			671.75 (66.56)b	43.49 (0.99)
IR 70369 A	0.27 (0.12)	0.12 (1.01)	0.28 (0.07)	0.21 (0.04)	0.12 (0.03)	0.02 (1.00)a	1.00 (0.00)	0.00 (0.00)	298.50 (36.75)a	36.51 (6.44)
IR 70369 B	0.19 (0.05)	0.22 (0.04)	0.28 (0.01)	0.15 (0.04)	0.15 (0.04)	0.01 (0.00)a	0.94 (0.06)	0.33 (0.17)	528.00 (141.92)ab	44.15 (11.13)
IR72889-46-3-2-1 R	0.22 (0.03)	0.21 (0.02)	0.18 (0.03)	0.17 (0.02)	0.17 (0.06)	0.04 (0.01)b	1.00 (0.00)	0.19 (0.11)	231.00 (73.68)a	24.20 (5.99)
F-Accession	0.354ns	2.703ns	2.020ns	0.601ns	0.934ns	13.449***			5.313*	1.697ns

IR 80637 H	0.20 (0.04)	0.25 (0.05)	0.30 (0.07)	0.16 (0.05)	0.08 (0.04)	0.01 (0.00)	0.91 (0.05)	0.25 (0.25)	466.75 (161.55)	47.97 (7.17)
IR 73328 A	0.21 (0.04)	0.26 (0.01)	0.28 (0.04)	0.17 (0.02)	0.07 (0.01)	0.02 (0.01)	0.86 (0.10)	0.28 (0.09)	410.00 (37.31)	43.57 (3.53)
IR 73328 B	0.38 (0.15)	0.12 (1.04)	0.19 (0.05)	0.17 (0.07)	0.12 (0.03)	0.02 (1.01)	1.00 (0.00)	0.18 (0.07)	385.00 (62.96)	38.86 (1.96)
IR73013-95-1-3-2 R	0.21 (0.09)	0.18 (0.06)	0.25 (0.02)	0.20 (0.07)	0.15 (0.10)	0.02 (0.01)	0.76 (0.12)	0.14 (0.07)	282.25 (94.35)	29.68 (5.30)
F-Accession	0.839na	2.077ns	0.900ns	0.110ns	0.507ns	0.066ns	1.915ns (3,10)	0.154ns (3,10)	0.590ns	2.559ns
IR 82385 H	0.09 (0.04)	0.12 (0.02)ab	0.31 (0.07)	0.22 (0.03)	0.17 (0.06)	0.08 (0.02)	1.00 (0.00)b	0.14 (0.08)	188.00 (76.46)	23.79 (7.07)
IR 79125 A	0.29 (0.18)	0.06 (1.03)a	0.11 (0.05)	0.12 (0.03)	0.37 (0.17)	0.05 (1.03)	1.00 (0.00)b	0.24 (0.13)	288.00 (99.58)	25.88 (5.72)
IR 79125 B	0.17 (0.05)	0.19 (0.04)b	0.17 (0.02)	0.20 (0.04)	0.19 (0.04)	0.08 (0.02)	0.78 (0.04)a	0.30 (0.06)	180.75 (49.73)	29.73 (3.72)
IR73717-46-1-3-3 R	0.26 (0.06)	0.22 (0.02)b	0.25 (0.05)	0.12 (0.02)	0.12 (0.04)	0.02 (0.01)	0.89 (0.11)ab	0.22 (0.22)	279.75 (79.12)	33.83 (3.94)
F-Accession	0.806ns	5.517*	3.180ns	2.671ns	1.146ns	1.581ns	4.653* (3,10)	0.366ns (3,10)	0.542ns	0.699ns
IR 82363 H	0.15 (0.05)	0.21 (0.06)	0.30 (0.07)	0.17 (0.04)	0.17 (0.05)	0.02 (0.01)	0.80 (0.20)	0.41 (0.21)	264.75 (81.22)a	45.89 (8.86)ab
IR 68897 A	0.19 (0.04)	0.28 (0.09)	0.22 (0.02)	0.15 (0.05)	0.14 (0.03)	0.00 (0.00)	1.00 (0.00)	0.33 (0.17)	255.50 (49.67)a	28.93 (5.60)a
IR 68897 B	0.28 (0.06)	0.29 (0.04)	0.21 (0.04)	0.13 (0.05)	0.06 (0.01)	0.02 (0.01)	0.93 (0.07)	0.08 (0.05)	816.50 (183.90)b	60.64 (7.62)b
SRT 3 R	0.23 (0.10)	0.17 (1.04)	0.12 (0.05)	0.16 (0.04)	0.27 (0.16)	0.05 (1.01)	0.90 (0.08)	0.48 (0.28)	323.00 (190.98)ab	28.99 (9.22)a
F-Accession	0.711ns	0.991ns	2.245ns	0.105ns	1.030ns	3.429ns	0.676ns (9)	0.892ns (9)	3.658*	3.686*
IR 80814 H	0.31 (0.03)	0.23 (0.03)	0.21 (0.02)	0.18 (0.03)	0.06 (0.02)	0.01 (0.00)	1.00 (0.00)	0.00 (0.00)a	810.00 (217.84)b	69.05 (11.43)b
IR 70369 A	0.27 (0.12)	0.12 (1.01)	0.28 (0.07)	0.21 (0.04)	0.12 (0.03)	0.02 (1.00)	1.00 (0.00)	0.00 (0.00)a	298.50 (36.75)ab	36.51 (6.44)ab
IR 70369 B	0.19 (0.05)	0.22 (0.04)	0.28 (0.01)	0.15 (0.04)	0.15 (0.04)	0.01 (0.00)	0.94 (0.06)	0.33 (0.17)b	528.00 (141.92)ab	44.15 (11.13)ab
IR69712-154-2-3-1-3 R	0.19 (0.07)	0.21 (0.04)	0.23 (0.07)	0.17 (0.04)	0.17 (0.05)	0.04 (0.02)	1.00 (0.00)	0.00 (0.00)a	165.25 (60.84)a	25.46 (7.40)a
F-Accession	0.635ns	2.175ns	0.458ns	0.402ns	1.508ns	2.515ns	1.310ns (3,10)	5.238* (3,10)	4.400*	3.906*
IR 81955 H	0.25 (0.06)	0.26 (0.01)b	0.21 (0.03)	0.10 (0.03)	0.15 (0.05)	0.03 (0.03)	0.75 (0.14)	0.25 (0.14)	680.50 (218.52)	50.33 (14.16)
IR 70369 A	0.27 (0.12)	0.12 (1.01)b	0.28 (0.07)	0.21 (0.04)	0.12 (0.03)	0.02 (1.00)	1.00 (0.00)	0.00 (0.00)	298.50 (36.75)	36.51 (6.44)
IR 70369 B	0.19 (0.05)	0.22 (0.04)a	0.28 (0.01)	0.15 (0.04)	0.15 (0.04)	0.01 (0.00)	0.94 (0.06)	0.33 (0.17)	528.00 (141.92)	44.15 (11.13)
IR72998-93-3-3-2 R	0.16 (0.07)	0.14 (0.05)a	0.18 (0.07)	0.15 (0.05)	0.28 (0.13)	0.09 (0.03)	0.96 (0.04)	0.04 (0.04)	247.50 (195.58)	23.46 (9.37)
F-Accession	0.405ns	3.692*	0.925ns	1.100ns	0.962ns	2.602ns	1.855ns (3,11)	2.314ns (3,11)	1.521ns	1.181ns
IR 81956 H	0.20 (0.08)	0.16 (0.06)	0.26 (0.03)	0.25 (0.10)	0.12 (0.06)	0.01 (0.00)	0.92 (0.08)	0.08 (0.08)ab	597.50 (89.06)	55.34 (3.26)
IR 70369 A	0.27 (0.12)	0.12 (1.01)	0.28 (0.07)	0.21 (0.04)	0.12 (0.03)	0.02 (1.00)	1.00 (0.00)	0.00 (0.00)a	298.50 (36.75)	36.51 (6.44)
IR 70369 B	0.19 (0.05)	0.22 (0.04)	0.28 (0.01)	0.15 (0.04)	0.15 (0.04)	0.01 (0.00)	0.94 (0.06)	0.33 (0.17)b	528.00 (141.92)	44.15 (11.13)
IR73003-73-2-3-2 R	0.25 (0.08)	0.18 (0.05)	0.26 (0.03)	0.13 (0.05)	0.11 (0.05)	0.07 (0.05)	1.00 (0.00)	0.00 (0.00)a	379.00 (160.30)	37.13 (6.61)
F-Accession	0.183ns	0.876ns	0.091ns	0.827ns	0.130ns	1.316ns	0.733ns (3,11)	3.582* (3,11)	1.351ns	1.395ns
IR 81958 H	0.18 (0.09)	0.25 (0.07)	0.22 (0.05)	0.17 (0.07)	0.14 (0.05)	0.03 (0.01)	0.91 (0.07)	0.09 (0.07)	548.50 (192.25)	47.65 (12.39)

IR 70369 A	0.27 (0.12)	0.12 (1.01)	0.28 (0.07)	0.21 (0.04)	0.12 (0.03)	0.02 (1.00)	1.00 (0.00)	0.00 (0.00)	298.50 (36.75)	36.51 (6.44)
IR 70369 B	0.19 (0.05)	0.22 (0.04)	0.28 (0.01)	0.15 (0.04)	0.15 (0.04)	0.01 (0.00)	0.94 (0.06)	0.33 (0.17)	528.00 (141.92)	44.15 (11.13)
IR73013-95-1-3-2 R	0.21 (0.09)	0.18 (0.06)	0.25 (0.02)	0.20 (0.07)	0.15 (0.10)	0.02 (0.01)	0.76 (0.12)	0.14 (0.07)	282.25 (94.35)	29.68 (5.30)
F-Accession	0.194ns	1.236ns	0.418ns	0.211ns	0.070ns	1.244ns	2.325ns (3,9)	2.648ns (3,9)	1.223ns	0.742ns
IR 80228 H	0.08 (0.03)	0.15 (0.05)	0.33 (0.02)b	0.24 (0.06)	0.14 (0.04)	0.06 (0.02)	0.84 (0.08)	0.16 (0.08)	189.75 (93.02)	21.54 (6.33)
IR 70369 A	0.27 (0.12)	0.12 (1.01)	0.28 (0.07)ab	0.21 (0.04)	0.12 (0.03)	0.02 (1.00)	1.00 (0.00)	0.00 (0.00)	298.50 (36.75)	36.51 (6.44)
IR 70369 B	0.19 (0.05)	0.22 (0.04)	0.28 (0.01)ab	0.15 (0.04)	0.15 (0.04)	0.01 (0.00)	0.94 (0.06)	0.33 (0.17)	528.00 (141.92)	44.15 (11.13)
IR73885-1-4-3-2-1-6R	0.23 (0.08)	0.18 (0.08)	0.12 (0.05)a	0.21 (0.0)	0.22 (0.10)	0.04 (0.02)	0.94 (0.04)	0.06 (0.04)	291.25 (151.45)	30.41 (12.97)
F-Accession	1.072ns	0.689ns	3.901*	0.457ns	0.563ns	2.181ns	1.818ns (3,10)	3.164ns (3,10)	1.541ns	0.980ns
IR 81949 H	0.21 (0.07)	0.19 (0.05)	0.31 (0.06)	0.23 (0.05)	0.06 (0.01)	0.01 (0.00)	0.95 (0.05)	0.05 (0.05)	391.25 (35.26)ab	32.49 (4.80)a
IR 68897 A	0.19 (0.04)	0.28 (0.09)	0.22 (0.02)	0.15 (0.05)	0.14 (0.03)	0.00 (0.00)	1.00 (0.00)	0.33 (0.17)	255.50 (49.67)a	28.93 (5.60)a
IR 68897 B	0.28 (0.06)	0.29 (0.04)	0.21 (0.04)	0.13 (0.05)	0.06 (0.01)	0.02 (0.01)	0.93 (0.07)	0.08 (0.05)	816.50 (183.90)b	60.64 (7.62)b
IR73013-95-1-3-2 R	0.21 (0.09)	0.18 (0.06)	0.25 (0.02)	0.20 (0.07)	0.15 (0.10)	0.02 (0.01)	0.76 (0.12)	0.14 (0.07)	282.25 (94.35)a	29.68 (5.30)a
F-Accession	0.353ns	1.019ns	1.120ns	0.618ns	0.975ns	0.748ns	1.802ns (3,10)	2.119ns (3,10)	5.830*	6.587**
IR 86167 H	0.24 (0.10)ab	0.20 (1.06)	0.18 (0.06)	0.09 (0.04)	0.20 (0.14)	0.10 (1.07)	0.91 (0.09)	0.09 (0.09)	121.50 (39.22)ab	21.94 (4.33)
IR80156A	0.41 (0.05)b	0.17 (0.03)	0.17 (0.02)	0.12 (0.03)	0.09 (0.01)	0.03 (0.01)	0.90 (0.10)	0.08 (0.04)	333.25 (41.54)b	72.28 (31.19)
IR80156B	0.06 (0.03)a	0.10 (0.05)	0.28 (0.10)	0.27 (0.05)	0.13 (0.06)	0.16 (0.05)	1.00 (0.00)	0.33 (0.33)	60.50 (35.89)a	7.32 (2.90)
IR73013-95-1-3-2 R	0.21 (0.09)ab	0.18 (0.06)	0.25 (0.02)	0.20 (0.07)	0.15 (0.10)	0.02 (0.01)	0.76 (0.12)	0.14 (0.07)	282.25 (94.35)ab	29.68 (5.30)
F-Accession	4.169*	0.650ns	0.749ns	2.715ns	0.264ns	0.725ns	1.242ns (3,8)	0.448ns (3,8)	4.959*	3.029ns

1: Numbers are means (SEM)(N=4); DF = 4,15 unless indicated in parentheses, ns = P > 0.05, * = P < 0.05, ** = P < 0.01, *** = P < 0.005; lowercase letters indicate homogenous line groups (Tukey: P > 0.05). 2: Note that some of the results for BPH biomass have been summarized by plant-type and presented in a related paper by Horgan et al (2016); the data and information by accession has not been previously published.

Table S2: Responses by BPH to 15 hybrid rice varieties indicating potential sources of resistance/susceptibility based on heterosis or heterbeltiosis with parental lines. Information related to the parental lines and statistical results used to determine heterosis/heterobeltiosis are presented in Table S1. Data is summarized in Figure 1 or the main text.

Accession	Development Stages[1]						Brachypterous (Proportion)[1]	Female (Proportion)[1]	Total Number of Individuals[1,2]	Totla Dry Weight1[,2]
	1st instars[2]	2nd instars[2]	3rd instars[2]	4th instars[2]	5th instars[2]	adults				
IR82396H	0.28 (0.06)	0.22 (0.07)	0.21 (0.02)	0.20 (0.080	0.06 (0.03)	0.04 (0.03)	0.67 (0.33)	0.44 (0.29)	449.00 (161.78)ab	28.35 (6.21)a
IR 86167 H	0.24 (0.10)	0.20 (1.06)	0.18 (0.06)	0.09 (0.04)	0.20 (0.14)	0.10 (1.07)	0.91 (0.09)	0.09 (0.09)	121.50 (39.22)a	21.94 (4.33)a
IR 81954 H	0.17 (0.05)	0.21 (0.03)	0.38 (0.08)	0.15 (0.03)	0.08 (0.03)	0.00 (0.00)			671.75 (66.56)ab	43.49 (0.99)ab
IR 80814 H	0.31 (0.03)	0.23 (0.03)	0.21 (0.02)	0.18 (0.03)	0.06 (0.02)	0.01 (0.00)	1.00 (0.00)	0.00 (0.00)a	810.00 (217.84)b	69.05 (11.43)b
IR 81955 H	0.25 (0.06)	0.26 (0.01)	0.21 (0.03)	0.10 (0.03)	0.15 (0.05)	0.03 (0.03)	0.75 (0.14)	0.25 (0.14)	680.50 (218.52)ab	50.33 (14.16)ab
IR 81956 H	0.20 (0.08)	0.16 (0.06)	0.26 (0.03)	0.25 (0.10)	0.12 (0.06)	0.01 (0.00)	0.92 (0.08)	0.08 (0.08)	597.50 (89.06)ab	55.34 (3.26)ab
IR 81958 H	0.18 (0.09)	0.25 (0.07)	0.22 (0.05)	0.17 (0.07)	0.14 (0.05)	0.03 (0.01)	0.91 (0.07)	0.09 (0.07)	548.50 (192.25)ab	47.65 (12.39)ab
IR 80228 H	0.08 (0.03)	0.15 (0.05)	0.33 (0.02)	0.24 (0.06)	0.14 (0.04)	0.06 (0.02)	0.84 (0.08)	0.16 (0.08)	189.75 (93.02)ab	21.54 (6.33)a
IR 80637 H	0.20 (0.04)	0.25 (0.05)	0.30 (0.07)	0.16 (0.05)	0.08 (0.04)	0.01 (0.00)	0.91 (0.05)	0.25 (0.25)	466.75 (161.55)ab	47.97 (7.17)ab
IR 81949 H	0.21 (0.07)	0.19 (0.05)	0.31 (0.06)	0.23 (0.05)	0.06 (0.01)	0.01 (0.00)	0.95 (0.05)	0.05 (0.05)	391.25 (35.26)ab	32.49 (4.80)ab cd
IR 82363 H	0.15 (0.05)	0.21 (0.06)	0.30 (0.07)	0.17 (0.04)	0.17 (0.05)	0.02 (0.01)	0.80 (0.20)	0.41 (0.21)	264.75 (81.22)ab	45.89 (8.86)ab*
IR 82391 H	0.36 (0.07)	0.23 (0.04)	0.16 (0.03)	0.12 (0.03)	0.10 (0.02)	0.03 (0.01)	0.69 (0.20)	0.16 (0.06)	254.00 (47.22)ab	30.66 (6.28)ab
IR 84714 H	0.34 (0.08)	0.23 (0.01)	0.17 (0.02)	0.14 (0.04)	0.10 (0.04)	0.02 (0.01)	1.00 (0.00)	0.19 (0.11)	330.25 (77.29)ab	33.47 (6.89)ab
IR 85471 H	0.11 (0.05)	0.23 (0.05)	0.25 (0.06)	0.26 (0.03)	0.12 (0.02)	0.03 (0.01)	0.85 (0.10)	0.17 (0.10)*	458.00 (170.76)ab	52.52 (8.08)ab
IR 82385 H	0.09 (0.04)	0.12 (0.02)*	0.31 (0.07)	0.22 (0.03)	0.17 (0.06)	0.08 (0.02)	1.00 (0.00)b	0.14 (0.08)	188.00 (76.46)ab	23.79 (7.07)a
F-Accession	1.797ns	0.693ns	1.520ns	1.208ns	0.683ns	1.575ns	0.818ns [13,34]	1.546ns [13,34]	2.455* [14,45]	3.118*** [14,45]

1: Numbers are means (SEM)(N=4); DF = 14,84 unless indicated in square brackets; Wilk's lambda for development stages = 1.167 and is not significant; ns = $P > 0.05$, * = $P < 0.05$, *** = $P < 0.005$; lowercase letters indicate homogenous hybrid groups (Tukey: $P > 0.05$). 2: Blue = heterosis; green = heterobeltiosis for resistance; red = heterobeltiosis for susceptibility; Wilk's lambda for development stages = 1.167 and is not significant.

Table S3: Oviposition by BPH and WBPH during choice experiments with hybrids and associated parental lines.

Variety	BPH			WBPH		
	Clusters per Plant[1]	Eggs per Plant[1,2]	Plant Biomass (dry mg)[2]	Clusters per Plant[1]	Eggs per Plant[1]	Plant Biomass (dry mg)[1]
IR82396 H	17.50 (10.25)	85.67 (44.28)	0.47 (0.03)	16.17 (3.89)	110.50 (27.95)	0.33 (0.05)
IR80156 A	9.83 (3.75)	70.50 (28.92)	0.41 (0.03)	17.17 (5.95)	115.67 (40.65)	0.31 (0.04)
IR80156 B	25.33 (10.99)	142.00 (56.21)	0.54 (0.05)	5.00 (2.25)	39.67 (17.12)	0.26 (0.05)
IR46R	21.67 (12.28)	123.17 (63.05)	0.52 (0.06)	14.33 (4.16)	130.50 (35.45)	0.31 (0.03)
F-line	0.460ns	0.483ns	1.704ns	0.369ns	1.346ns	0.361ns
F-biomass	0.202ns	0.288ns		0.879ns	0.765ns	
IR82391 H	28.67 (10.05)c	160.17 (51.49)b[ht}	0.48 (0.05)	11.83 (4.91)	126.00 (31.88)	0.33 (0.02)
IR79156 A	18.17 (7.27)bc	112.67 (42.98)ab	0.45 (0.05)	7.83 (3.23)	59.67 (23.81)	0.26 (0.04)
IR79156 B	5.33 (2.80)ab	31.33 (17.80)a	0.38 (0.06)	5.67 (2.64)	48.00 (24.19)	0.21 (0.03)
IR60819-34-2 R	8.00 (3.84)a	43.50 (23.85)a	0.47 (0.04)	12.50 (2.18)	119.83 (22.48)	0.32 (0.03)
F-line	7.333***	6.227***	0.901ns	0.476ns	1.309ns	2.476ns
F-biomass	1.480ns	1.926ns		1.102ns	0.482ns	
IR84714 H	6.50 (3.45)	45.67 (24.03)a[ht]′	0.48 (0.07)	6.17 (2.18)ab	50.83 (19.75)ab	0.41 (0.04)
IR80559 A	27.00 (13.66)	171.00 (72.83)b	0.54 (0.02)	21.33 (6.99)b	173.67 (50.53)b	0.41 (0.04)
IR80559 B	10.00 (3.47)	59.83 (21.02)ab	0.44 (0.02)	13.67 (4.00)b	97.50 (27.58)ab	0.30 (0.05)
IR60819-34-2 R	15.50 (8.44)	90.50 (49.66)ab	0.48 (0.04)	2.33 (0.84)a	19.50 (8.68)a	0.31 (0.03)
F-line	2.113ns	3.403*	0.962ns	5.460**	5.192**	2.047ns
F-biomass	0.176ns	0.873ns		4.641*	3.884ns	
IR85471 H	5.83 (3.76)	28.83 (18.16)	0.44 (0.04)	5.50 (2.17)	40.33 (16.13)	0.37 (0.04)
IR80564 A	13.83 (6.93)	90.67 (44.65)	0.45 (0.05)	13.50 (3.65)	105.17 (29.29)	0.29 (0.03)
IR80564 B	11.50 (6.35)	62.67 (31.38)	0.34 (0.03)	16.67 (5.97)	121.33 (42.60)	0.28 (0.02)
IR60819-34-2 R	15.33 (6.30)	96.67 (40.35)	0.47 (0.02)	6.00 (1.63)	53.83 (13.35)	0.32 (0.05)
F-line	0.863ns	1.004ns	2.445ns	1.227ns	0.921ns	0.995ns
F-biomass	0.212ns	0.429ns		0.250ns	0.151ns	
IR81954 H	32.17 (12.78)	153.00 (71.58)	0.58 (0.02)b	14.00 (5.60)	130.33 (45.61)	0.41 (0.03)
IR70369 A	14.17 (2.79)	105.17 (27.26)	0.52 (0.03)b	14.33 (4.65)	116.67 (35.77)	0.32 (0.06)
IR70369 B	8.33 (3.11)	53.17 (25.58)	0.32 (0.09)a	2.50 (1.17)	23.17 (13.04)	0.22 (0.09)
IR72889-46-3-2-1 R	25.17 (10.42)	146.67 (48.47)	0.44 (0.03)ab	13.50 (5.65)	117.83 (51.93)	0.24 (0.06)
F-line	0.160ns	0.806ns	5.311**	0.559ns	0.612ns	1.826ns
F-biomass	4.061ns	1.477ns		0.414ns	0.857ns	
IR80637 H	18.33 (7.17)	98.17 (34.83)	0.47 (0.04)	18.00 (6.33)	147.83 (53.81)	0.30 (0.06)

IR73328 A	19.67 (12.51)	99.17 (64.27)	0.48 (0.05)	7.00 (3.01)	52.33 (24.51)	0.26 (0.06)
IR73328 B	20.00 (9.58)	144.67 (70.83)	0.44 (0.05)	13.67 (3.84)	98.83 (31.62)	0.24 (0.06)
IR73013-95-1-3-2 R	18.50 (6.40)	109.83 (48.69)	0.36 (0.02)	5.83 (2.41)	56.67 (24.44)	0.28 (0.03)
F-line	0.328ns	0.328ns	1.577ns	1.544ns	1.544ns	0.262ns
F-biomass	2.275ns	2.275ns		0.001ns	0.001ns	
IR82385 H	9.50 (3.65)	59.50 (24.48)	0.46 (0.05)	9.17 (3.13)	86.17 (37.24)	0.32 (0.04)
IR79125 A	30.50 (18.11)	137.50 (81.20)	0.44 (0.06)	10.16 (2.89)	90.67 (24.53)	0.25 (0.03)
IR79125 B	16.83 (7.88)	90.00 (33.97)	0.33 (0.04)	4.00 (1.21)	37.17 (15.41)	0.27 (0.03)
IR73717-46-1-3-3 R	25.83 (15.51)	142.33 (87.20)	0.35 (0.03)	14.83 (5.48)	106.33 (38.06)	0.28 (0.04)
F-line	0.626ns	0.421ns	1.831ns	1.718ns	2.937ns	0.585ns
F-biomass	1.459ns	0.949ns		0.363ns	0.229ns	
IR 82363 H	10.50 (8.79)	58.33 (51.04)	0.46 (0.03)	13.33 (2.80)	106.50 (26.81)	0.31 (0.07)
IR68897 A	14.33 (5.87)	92.67 (36.52)	0.39 (0.03)	11.50 (3.79)	94.83 (31.59)	0.37 (0.03)
IR68897 B	18.33 (13.06)	77.50 (50.24)	0.38 (0.03)	7.50 (4.27)	47.00 (27.58)	0.31 (0.05)
SRT 3 R	32.17 (14.66)	184.33 (68.34)	0.46 (0.04)	12.67 (4.27)	97.83 (40.83)	0.37 (0.03)
F-line	1.951ns	1.677ns	1.513ns	1.455ns	2.168ns	0.537ns
F-biomass	0.041ns	0.022ns		0.008ns	0.004ns	

1: Numbers are means (SEM) (N = 6); DF = 3,19 for accessions, 1,19 for covariate 'biomass'; ns = P > 0.05, * = P < 0.05, ** = P < 0.01, *** = P < 0.005; lowercase letters indicate homogenous variety groups (Tukey P > 0.05); 2: ht = heterosis for resistance; ht' = heterosis for resistance associated with plant size

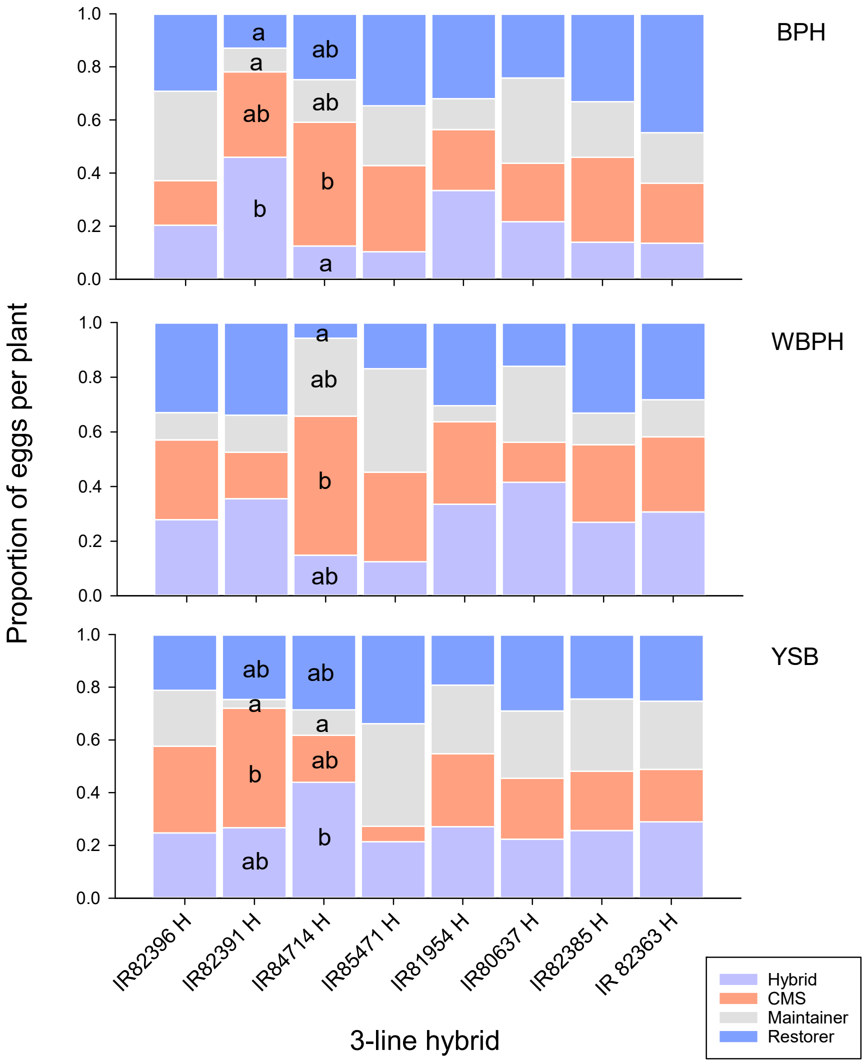

Figure S1: Proportion of eggs laid on hybrid rice and associated parental lines in a series of choice experiments (N=6). Significant plant-type effects on the proportions of eggs laid are indicated by lowercase letters as relevant to homogenous plant groups. Note that IR82391H shows heterobeltiosis for susceptibility to BPH (relative to the maintainer line [B-line]) or heterosis (relative to the CMS-line [A-line]); IR84714H shows heterosis for resistance to BPH (relative to the CMS-line [A-line]). There are no other cases of heterosis for resistance. BPH = brown planthopper, WBPH = whitebacked planthopper, YSB = yellow stemborer.

Table S4: Results from bioassays with WBPH on 15 hybrid rice varieties and their female (A and B lines) and male (restorer/R lines) parents. Summarized information from this table is presented in Table S5 and Figure 1.

Accession	Development Stages[1]						**Brachypterous (Proportion)[1]**	**Female (Proportion)[1]**	**Total Number of Individuals[1]**	**Total Dry Weight[1,2]**
	**1st instars**	**2nd instars**	**3rd instars**	**4th instars**	**5th instars**	**Adults**				
IR82396H	0.17 (0.06)	0.25 (0.04)	0.25 (0.05)b	0.09 (0.00)	0.14 (0.05)	0.05 (0.01)a	0.87 (0.10)	0.48 (0.26)	207.67 (77.41)b	21.22 (9.22)
IR80156A	0.02 (0.01)	0.12 (0.08)	0.13 (0.08)ab	0.11 (0.04)	0.28 (0.09)	0.33 (0.16)ab	0.52 (0.04)	0.57 (0.05)	72.00 (20.42)ab	11.03 (0.81)
IR80156B	0.00 (0.00)	0.00 (0.00)	0.03 (0.02)a	0.09 (0.03)	0.29 (0.09)	0.59 (0.13)ab	0.63 (0.08)	0.67 (0.09)	55.25 (10.66)a	16.65 (3.70)
IR46R	0.00 (0.00)	0.00 (0.00)	0.00 (0.00)a	0.03 (0.07)	0.33 (0.12)	0.64 (0.14)b	0.81 (0.11)	0.67 (0.12)	23.00 (8.26)a	6.07 (2.16)
F-Accession			7.115*** (3,11)	1.374ns (3,11)	0.649ns (3,11)	3.726* (3,11)	3.229ns (3,11)	0.419ns (3,11)	5.487* (3,11)	2.298ns (3,11)
IR 82391 H	0.00 (0.00)	0.06 (0.06)	0.04 (0.04)	0.13 (0.10)	0.20 (0.12)	0.56 (0.26)	0.64 (0.13)	0.69 (0.11)	21.50 (10.14)	3.36 (1.05)
IR 79156 A	0.00 (0.00)	0.00 (0.00)	0.00 (0.00)	0.09 (0.05)	0.44 (0.17)	0.47 (0.21)	0.35 (0.14)	0.57 (0.17)	19.75 (8.93)	4.61 (1.87)
IR 79156 B	0.00 (0.00)	0.02 (0.02)	0.19 (0.11)	0.13 (0.05)	0.23 (0.11)	0.43 (0.20)	0.57 (0.19)	0.57 (0.09)	62.50 (33.94)	11.08 (4.50)
IR60819-34-2	0.00 (0.00)	0.03 (0.03)	0.08 (0.08)	0.14 (0.05)	0.29 (0.04)	0.45 (0.13)	0.54 (0.19)	0.57 (0.08)	46.50 (16.24)	6.89 (2.26)
F-Accession				0.128ns	0.840ns	0.080ns	1.112ns	0.260ns	1.065ns	1.544ns
IR 84714 H	0.00 (0.00)	0.00 (0.09)	0.09 (0.06)	0.08 (0.07)	0.24 (0.06)	0.59 (0.15)	0.37 (0.14)	0.43 (0.17)	41.00 (21.08)	5.81 (3.57)
IR 80559 A	0.00 (0.00)	0.04 (0.02)	0.15 (0.09)	0.10 (0.07)	0.28 (0.07)	0.44 (0.17)	0.37 (0.17)	0.38 (0.08)	109.25 (36.18)	20.81 (5.29)
IR 80559 B	0.00 (0.00)	0.04 (0.03)	0.18 (0.09)	0.14 (0.06)	0.21 (0.09)	0.42 (0.20)	0.57 (0.16)	0.59 (0.07)	103.25 (42.83)	19.06 (5.81)
IR60819-34-2 R	0.00 (0.00)	0.00 (0.00)	0.03 (0.03)	0.18 (0.09)	0.32 (0.11)	0.48 (0.18)	0.31 (0.15)	0.62 (0.09)	68.75 (25.21)	16.73 (7.23)
F-Accession			0.885ns	0.333ns	0.273ns	0.193ns	1.029ns	1.164ns	0.961ns	1.432ns
IR 85471 H	0.04 (0.04)	0.20 (0.02)	0.30 (0.09)	0.10 (0.04)	0.25 (0.08)	0.11 (0.03)	0.37 (0.19)	0.24 (0.06)a	195.25 (100.51)	41.51 (17.42)
IR 80564 A	0.07 (0.02)	0.17 (0.06)	0.16 (0.06)	0.15 (0.05)	0.17 (0.07)	0.28 (0.24)	0.28 (0.16)	0.42 (0.08)ab	80.25 (26.24)	11.56 (3.32)
IR 80564 B	0.00 (0.00)	0.10 (0.10)	0.12 (0.12)	0.34 (0.07)	0.25 (0.07)	0.20 (0.04)	0.52 (0.18)	0.57 (0.13)ab	43.33 (21.37)	5.89 (2.76)
IR60819-34-2 R	0.00 (0.00)	0.00 (0.00)	0.03 (0.03)	0.18 (0.09)	0.32 (0.11)	0.48 (0.18)	0.31 (0.15)	0.62 (0.09)b	68.75 (25.21)	16.73 (7.23)
F-Accession			2.580ns (3,11)	2.002ns (3,11)	0.529ns (3,11)	1.004ns (3,11)	0.397ns (3,11)	4.072* (3,11)	1.347ns (3,11)	2.288ns (3,11)
IR 81954 H	0.00 (0.00)	0.03 (0.02)	0.10 (0.05)	0.22 (0.04)	0.38 (0.07)	0.27 (0.08)	0.27 (0.11)a	0.33 (0.05)	68.00 (28.69)	13.97 (5.31)
IR 70369 A	0.00 (0.00)	0.10 (0.08)	0.16 (0.10)	0.10 (0.06)	0.11 (0.07)	0.52 (0.28)	0.75 (0.13)b	0.47 (0.21)	126.00 (81.45)	26.76 (12.82)
IR 70369 B	0.00 (0.00)	0.00 (0.00)	0.07 (0.07)	0.03 (0.02)	0.51 (0.07)	0.39 (0.05)	0.47 (0.09)ab	0.61 (0.21)	66.67 (20.50)	15.34 (6.64)
IR72889-46-3-2-1 R	0.00 (0.00)	0.02 (0.02)	0.08 (0.08)	0.18 (0.07)	0.26 (0.12)	0.46 (0.22)	0.38 (0.03)a	0.38 (0.03)	71.00 (31.12)	13.27 (5.03)
F-Accession				1.970ns (3,10)	3.576ns (3,10)	0.248ns (3,10)	6.927** (3,9)	0.727ns (3,9)	0.317ns (3,10)	0.572ns (3,10)
IR 80637 H	0.00 (0.00)	0.03 (0.02)	0.11 (0.07)	0.10 (0.06)	0.22 (0.06)	0.54 (0.19)	0.49 (0.09)	0.54 (0.07)	79.75 (40.53)	18.75 (9.89)
IR 73328 A	0.00 (0.00)	0.00 (0.00)	0.00 (0.00)	0.03 (0.03)	0.10 (0.05)	0.87 (0.06)	0.58 (0.06)	0.58 (0.06)	26.25 (10.96)	7.35 (3.82)

IR 73328 B	0.00 (0.00)	0.00 (0.00)	0.02 (0.02)	0.06 (0.04)	0.19 (0.07)	0.73 (0.11)	0.47 (0.11)	0.55 (0.12)	49.75 (10.85)	19.10 (4.41)
IR73013-95-1-3-2 R	0.00 (0.00)	0.07 (0.07)	0.13 (0.13)	0.05 (0.05)	0.18 (0.11)	0.56 (0.28)	0.25 (0.18)	0.25 (0.18)	79.33 (71.35)	8.71 (6.74)
F-Accession				0.427ns (3,11)	0.612ns (3,11)	0.941ns (3,11)	1.456ns (3,11)	1.838ns (3,11)	0.522ns (3,11)	0.898ns (3,11)
IR 82385 H	0.00 (0.00)	0.03 (0.03)	0.06 (0.06)	0.07 (0.06)	0.32 (0.09)	0.52 (0.18)	0.45 (0.11)	0.49 (0.08)	51.50 (21.56)	14.82 (6.20)
IR 79125 A	0.00 (0.00)	0.00 (0.00)	0.00 (0.00)	0.00 (0.00)	0.45 (0.22)	0.55 (0.22)	0.64 (0.22)	0.64 (0.22)	8.33 (2.19)	2.19 (0.77)
IR 79125 B	0.08 (0.08)	0.14 (0.05)	0.20 (0.08)	0.33 (0.17)	0.23 (0.12)	0.02 (0.01)	1.00 (0.00)	1.00 (0.00)	45.25 (17.38)	6.68 (2.20)
IR73717-46-1-3-3 R	0.00 (0.00)	0.00 (0.00)	0.00 (0.00)	0.22 (0.01)	0.40 (0.15)	0.38 (0.16)	0.68 (0.04)	0.78 (0.06)	43.00 (18.25)	9.68 (3.24)
F-Accession					0.462ns (3,10)	2.663ns (3,10)	2.449ns (3,8)	2.664ns (3,8)	1.083ns (3,10)	1.686ns (3,10)
IR 82363 H	0.00 (0.00)	0.00 (0.00)	0.14 (0.10)	0.20 (0.10)	0.41 (0.07)b	0.25 (0.10)	0.28 (0.15)	0.50 (0.00)	67.67 (59.18)	26.13 (22.66)
IR 68897 A	0.00 (0.00)	0.00 (0.00)	0.00 (0.00)	0.07 (0.04)	0.48 (0.11)b	0.45 (0.09)	0.60 (0.10)	0.67 (0.17)	8.33 (2.03)	1.89 (0.48)
IR 68897 B	0.00 (0.00)	0.00 (0.00)	0.17 (0.17)	0.00 (0.00)	0.32 (0.04)ab	0.51 (0.13)	0.36 (0.22)	0.36 (0.22)	23.67 (12.20)	13.84 (9.44)
SRT 3 R	0.02 (0.02)	0.06 (0.06)	0.11 (0.09)	0.06 (0.04)	0.14 (0.07)a	0.61 (0.22)	0.72 (0.05)	0.72 (0.05)	77.50 (51.37)	12.65 (2.97)
F-Accession			0.448ns (3,9)	2.118ns (3,9)	3.925* (3,9)	0.863ns (3,9)	2.455ns (3,9)	1.561ns (3,9)	0.622ns (3,9)	0.714ns (3,9)
IR 80814 H	0.00 (0.00)	0.15 (0.10)	0.27 (0.05)	0.18 (0.06)	0.23 (0.09)ab	0.17 (0.06)	0.29 (0.10)a	0.71 (0.10)	99.25 (27.07)	13.37 (3.90)
IR 70369 A	0.00 (0.00)	0.10 (0.08)	0.16 (0.10)	0.10 (0.06)	0.11 (0.07)a	0.52 (0.28)	0.75 (0.13)b	0.47 (0.21)	126.00 (81.45)	26.76 (12.82)
IR 70369 B	0.00 (0.00)	0.00 (0.00)	0.07 (0.07)	0.03 (0.02)	0.51 (0.07)b	0.39 (0.05)	0.47 (0.09)ab	0.61 (0.21)	66.67 (20.50)	15.34 (6.64)
IR69712-154-2-3-1-3 R	0.00 (0.00)	0.04 (0.04)	0.06 (0.06)	0.17 (0.08)	0.25 (0.09)ab	0.48 (0.20)	0.68 (0.06)ab	0.32 (0.06)	67.25 (34.75)	8.20 (0.54)
F-Accession			1.761ns (3,11)	1.258ns (3,11)	3.599* (3,11)	0.772ns (3,11)	4.934* (3,10)	1.599ns (3,10)	0.320ns (3,11)	1.112ns (3,11)
IR 81955 H	0.00 (0.00)	0.08 (0.05)	0.21 (0.08)	0.21 (0.01)	0.36 (0.07)ab	0.14 (0.07)	0.47 (0.21)	0.53 (0.21)	45.25 (15.94)	5.49 (1.45)
IR 70369 A	0.00 (0.00)	0.10 (0.08)	0.16 (0.10)	0.10 (0.06)	0.11 (0.07)a	0.52 (0.28)	0.75 (0.13)	0.47 (0.21)	126.00 (81.45)	26.76 (12.82)
IR 70369 B	0.00 (0.00)	0.00 (0.00)	0.07 (0.07)	0.03 (0.02)	0.51 (0.07)b	0.39 (0.05)	0.47 (0.09)	0.61 (0.21)	66.67 (20.50)	15.34 (6.64)
IR72998-93-3-3-2 R	0.00 (0.00)	0.04 (0.04)	0.03 (0.03)	0.18 (0.11)	0.38 (0.04)ab	0.37 (0.14)	0.52 (0.14)	0.48 (0.14)	34.25 (12.22)	9.63 (1.29)
F-Accession			1.014ns (3,10)	1.764ns (3,10)	6.319* (3,10)	0.896ns (3,10)	0.650ns (3,9)	0.099ns (3,9)	0.847ns (3,10)	1.465ns (3,10)
IR 81956 H	0.07 (0.07)	0.17 (0.12)	0.25 (0.15)	0.09 (0.06)	0.12 (0.03)a	0.30 (0.26)	0.51 (0.29)	0.49 (0.29)	121.25 (70.18)	14.97 (4.01)
IR 70369 A	0.00 (0.00)	0.10 (0.08)	0.16 (0.10)	0.10 (0.06)	0.11 (0.07)a	0.52 (0.28)	0.75 (0.13)	0.47 (0.21)	126.00 (81.45)	26.76 (12.82)
IR 70369 B	0.00 (0.00)	0.00 (0.00)	0.07 (0.07)	0.03 (0.02)	0.51 (0.07)b	0.39 (0.05)	0.47 (0.09)	0.61 (0.21)	66.67 (20.50)	15.34 (6.64)
IR73003-73-2-3-2 R	0.00 (0.00)	0.00 (0.00)	0.00 (0.00)	0.07 (0.07)	0.18 (0.13)ab	0.75 (0.20)	0.52 (0.11)	0.48 (0.11)	25.75 (19.23)	8.37 (4.98)
F-Accession			1.135ns (3,9)	0.271ns (3,9)	5.299* (3,9)	0.672ns (3,9)	0.528ns (3,8)	0.094ns (3,8)	0.680ns (3,9)	0.755ns (3,9)
IR 81958 H	0.00 (0.00)	0.00 (0.00)	0.00 (0.00)	0.09 (0.07)	0.33 (0.16)	0.59 (0.21)	0.32 (0.03)	0.68 (0.03)	28.50 (16.78)	12.29 (6.36)
IR 70369 A	0.00 (0.00)	0.10 (0.08)	0.16 (0.10)	0.10 (0.06)	0.11 (0.17)	0.52 (0.28)	0.75 (0.13)	0.47 (0.21)	126.00 (81.45)	26.76 (12.82)
IR 70369 B	0.00 (0.00)	0.00 (0.00)	0.07 (0.07)	0.03 (0.02)	0.51 (0.17)	0.39 (0.05)	0.47 (0.09)	0.61 (0.21)	66.67 (20.50)	15.34 (6.64)
IR73013-95-1-3-2 R	0.00 (0.00)	0.07 (0.07)	0.13 (0.13)	0.05 (0.05)	0.18 (0.11)	0.56 (0.28)	0.25 (0.18)	0.25 (0.18)	79.33 (71.35)	8.71 (6.74)

F-Accession			0.613ns (3,9)	0.806ns (3,9)	2.921ns (3,9)	0.135ns (3,9)	3.373ns (3,8)	1.202ns (3,8)	0.555ns (3,8)	0.685ns (3,8)
IR 80228 H	0.00 (0.00)	0.02 (0.02)	0.16 (0.13)	0.07 (0.04)	0.27 (0.09)	0.48 (0.18)	0.59 (0.14)	0.41 (0.14)	95.00 (58.81)	12.43 (4.04)
IR 70369 A	0.00 (0.00)	0.10 (0.08)	0.16 (0.10)	0.10 (0.06)	0.11 (0.07)	0.52 (0.28)	0.75 (0.13)	0.47 (0.21)	126.00 (81.45)	26.76 (12.82)
IR 70369 B	0.00 (0.00)	0.00 (0.00)	0.07 (0.07)	0.03 (0.02)	0.51 (0.07)	0.39 (0.05)	0.47 (0.09)	0.61 (0.21)	66.67 (20.50)	15.34 (6.64)
IR73885-1-4-3-2-1-6R	0.00 (0.00)	0.00 (0.00)	0.01 (0.01)	0.09 (0.04)	0.24 (0.15)	0.66 (0.19)	0.54 (0.06)	0.46 (0.06)	39.50 (13.38)	12.00 (3.45)
F-Accession			0.650ns (3,11)	0.379ns (3,11)	2.269ns (3,11)	0.296ns (3,11)	0.971ns (3,10)	0.266ns (3,10)	0.476ns (3,11)	0.814ns (3,11)
IR 81949 H	0.00 (0.00)	0.01 (0.00)	0.10 (0.06)	0.08 (0.04)	0.29 (0.06)	0.51 (0.14)	0.44 (0.03)	0.56 (0.03)	70.25 (10.16)	13.70 (1.98)
IR 68897 A	0.00 (0.00)	0.00 (0.00)	0.00 (0.00)	0.07 (0.04)	0.48 (0.11)	0.45 (0.09)	0.60 (0.10)	0.67 (0.07)	8.33 (2.03)	1.89 (0.48)
IR 68897 B	0.00 (0.00)	0.00 (0.00)	0.17 (0.17)	0.00 (0.00)	0.32 (0.04)	0.51 (0.13)	0.36 (0.22)	0.36 (0.02)	23.67 (12.20)	13.84 (9.44)
IR73013-95-1-3-2 R	0.00 (0.00)	0.07 (0.07)	0.13 (0.13)	0.05 (0.05)	0.18 (0.11)	0.56 (0.28)	0.25 (0.18)	0.25 (0.18)	79.33 (71.35)	8.71 (6.74)
F-Accession			0.444ns (3,9)	0.806ns (3,9)	2.123ns (3,9)	0.064ns (3,9)	1.048ns (3,9)	1.466ns (3,9)	1.047ns (3,9)	1.055ns (3,9)
IR 86167 H	0.00 (0.00)	0.01 (0.01)	0.10 (0.05)	0.15 (0.04)	0.31 (0.09)	0.43 (0.15)	0.39 (0.11)	0.61 (0.11)	48.25 (16.84)	12.25 (1.09)
IR80156A	0.02 (0.01)	0.12 (0.08)	0.13 (0.08)	0.11 (0.04)	0.28 (0.09)	0.33 (0.16)	0.52 (0.04)	0.57 (0.05)	72.00 (20.42)	11.03 (0.81)
IR80156B	0.00 (0.00)	0.00 (0.00)	0.03 (0.02)	0.09 (0.03)	0.29 (0.09)	0.59 (0.13)	0.63 (0.08)	0.67 (0.09)	55.25 (10.66)	16.65 (3.70)
IR73013-95-1-3-2 R	0.00 (0.00)	0.07 (0.07)	0.13 (0.13)	0.05 (0.05)	0.18 (0.11)	0.56 (0.28)	0.25 (0.18)	0.25 (0.18)	79.33 (71.35)	8.71 (6.74)
F-Accession			0.466ns [3,10]	0.885ns [3,10]	0.310ns [3,10]	0.506ns [3,10]	2.522ns [3,10]	2.913ns [3,10]	0.203ns [3,10]	0.891ns [3,10]

1: Numbers are means (SEM)(N=4); DF = 4,15 unless indicated in square brackets, ns = P > 0.05, * = P < 0.05; lowercase letters indicate homogenous line groups (Tukey: P > 0.05). 2: Note that some of the results for WBPH biomass have been summarized by plant-type and presented in a related paper by Horgan et al (2016); the data and information by accession has not been previously published.

Table S5: Responses by WBPH to 15 hybrid rice varieties indicating potential sources of resistance/susceptibility based on heterosis or heterbeltiosis with parental lines. Information related to the parental lines and statistical results used to determine heterosis/heterobeltiosis are presented in Table S4. Data is summarized in Figure 1 of the main text.

Accession	Development Stages[1]						Brachypterous (Proportion)[1]	Female (Proportion)[1]	Total Number of Individuals[1]	Total Dry Weight (mg)[1]
	1st instars[1]	2nd instars[1]	3rd instars[1,2]	4th instars[1]	5th instars[1,2]	Adults[1,2]				
IR82396H	0.17 (0.06)b	0.25 (0.04)b	0.25 (0.05)	0.09 (0.00)	0.14 (0.05)	0.05 (0.01)	0.87 (0.10)	0.48 (0.26)	207.67 (77.41)	21.22 (9.22)
IR 86167 H	0.00 (0.00)a	0.01 (0.01)ab	0.10 (0.05)	0.15 (0.04)	0.31 (0.09)	0.43 (0.15)	0.39 (0.11)	0.61 (0.11)	48.25 (16.84)	12.25 (1.09)
IR 81954 H	0.00 (0.00)a	0.03 (0.02)ab	0.10 (0.05)	0.22 (0.04)	0.38 (0.07)	0.27 (0.08)	0.27 (0.11)a	0.33 (0.05)	68.00 (28.69)	13.97 (5.31)
IR 80814 H	0.00 (0.00)a	0.15 (0.10)ab	0.27 (0.05)	0.18 (0.06)	0.23 (0.09)	0.17 (0.06)	0.29 (0.10)a	0.71 (0.10)	99.25 (27.07)	13.37 (3.90)
IR 81955 H	0.00 (0.00)a	0.08 (0.05)ab	0.21 (0.08)	0.21 (0.01)	0.36 (0.07)	0.14 (0.07)	0.47 (0.21)	0.53 (0.21)	45.25 (15.94)	5.49 (1.45)
IR 81956 H	0.07 (0.07)ab	0.17 (0.12)ab	0.25 (0.15)	0.09 (0.06)	0.12 (0.03)	0.30 (0.26)	0.51 (0.29)	0.49 (0.29)	121.25 (70.18)	14.97 (4.01)
IR 81958 H	0.00 (0.00)a	0.00 (0.00)ab	0.00 (0.00)	0.09 (0.07)	0.33 (0.16)	0.59 (0.21)	0.32 (0.03)	0.68 (0.03)	28.50 (16.78)	12.29 (6.36)
IR 80228 H	0.00 (0.00)a	0.02 (0.02)ab	0.16 (0.13)	0.07 (0.04)	0.27 (0.09)	0.48 (0.18)	0.59 (0.14)	0.41 (0.14)	95.00 (58.81)	12.43 (4.04)
IR 80637 H	0.00 (0.00)a	0.03 (0.02)ab	0.11 (0.07)	0.10 (0.06)	0.22 (0.06)	0.54 (0.19)	0.49 (0.09)	0.54 (0.07)	79.75 (40.53)	18.75 (9.89)
IR 81949 H	0.00 (0.00)a	0.01 (0.00)ab	0.10 (0.06)	0.08 (0.04)	0.29 (0.06)	0.51 (0.14)	0.44 (0.03)	0.56 (0.03)	70.25 (10.16)	13.70 (1.98)
IR 82363 H	0.00 (0.00)a	0.00 (0.00)a	0.14 (0.10)	0.20 (0.10)	0.41 (0.07)	0.25 (0.10)	0.28 (0.15)	0.50 (0.00)	67.67 (59.18)	26.13 (22.66)
IR 82391 H	0.00 (0.00)a	0.06 (0.06)ab	0.04 (0.04)	0.13 (0.10)	0.20 (0.12)	0.56 (0.26)	0.64 (0.13)	0.69 (0.11)	21.50 (10.14)	3.36 (1.05)
IR 84714 H	0.00 (0.00)a	0.00 (0.09)a	0.09 (0.06)	0.08 (0.07)	0.24 (0.06)	0.59 (0.15)	0.37 (0.14)	0.43 (0.17)	41.00 (21.08)	5.81 (3.57)
IR 85471 H	0.04 (0.04)ab	0.20 (0.02)ab	0.30 (0.09)	0.10 (0.04)	0.25 (0.08)	0.11 (0.03)	0.37 (0.19)	0.24 (0.06)a	195.25 (100.51)	41.51 (17.42)
IR 82385 H	0.00 (0.00)a	0.03 (0.03)ab	0.06 (0.06)	0.07 (0.06)	0.32 (0.09)	0.52 (0.18)	0.45 (0.11)	0.49 (0.08)	51.50 (21.56)	14.82 (6.20)
	3.784***	2.594**	1.430ns	0.838ns	0.849ns	1.504ns	1.184ns [14,39]	0.967ns [14,39]	1.385ns [14,42]	1.291ns [14,38]

1: Numbers are means (SEM)(N=4); DF = 14,84 unless indicated in square brackets; Wilk's lambda for development stages = 1.148 and is not significant; ns = P > 0.05, ** = P < 0.01, *** = P < 0.005; lowercase letters indicate homogenous hybrid groups (Tukey: P > 0.05). 2: Blue = heterosis; red = heterobeltiosis for susceptibility

Table S6: Responses by YSB to 15 hybrid rice varieties and their female (A and B lines) and male (restorer lines) parents. The summarized information is presented in Table S7 and Figure 1.

Accession	Number of Individuals per Plant[1,2]		Development Time (Days)[1,2]		Weight of Adult (mg)[1,2]		Total Number of Individuals[1,3]	Total Dry Weight (mg)[1,3,4]	Females (Proportion)[1,3]
Accession	Female	Male	Female	Male	Female	Male			
IR82396H	0.67 (0.33)	0.67 (0.33)	37.17 (1.96)	38.33 (3.48)	9.55 (1.28)	5.42 (0.49)	1.33 (0.49)	9.72 (3.68)	0.46 (0.21)
IR80156A	0.50 (0.34)	0.17 (0.17)	35.25 (5.25)	30.00	7.38 (0.83)	6.10 (0.00)	0.67 (0.42)	4.57 (2.89)	0.75 (0.25)
IR80156B	0.50 (0.22)	0.67 (0.33)	41.00 (2.08)	47.33 (6.36)	9.50 (0.72)	3.50 (1.30)	1.17 (0.40)	6.72 (2.92)	0.47 (0.23)
IR46R	0.33 (0.21)	0.50 (0.22)	40.50 (6.50)	35.00 (3.46)	11.60 (4.30)	3.90 (0.35)	0.83 (0.31)	5.82 (2.56)	0.38 (0.24)
F-Accession (A)	0.5959ns		1.982ns [3,12]		0.316ns [3,11]		0.546ns	0.523ns	0.300ns [3,11]
F-Sex (S)	0.001ns		0.060ns		18.523***				
F-AxS	0.357ns		0.810ns		1.300ns				
IR 82391 H	1.33 (0.42)b	2.00 (0.68)	35.40 (0.60)	34.98 (2.02)	9.38 (0.86)	4.12 (0.30)	3.33 (0.80)b	20.73 (4.98)b	0.39 (0.11)
IR 79156 A	0.33 (0.21)a	0.50 (0.34)	34.50 (0.50)	31.50 (2.50)	7.75 (1.95)	5.20 (0.20)	0.83 (0.48)a	5.15 (3.12)a	0.44 (0.29)
IR 79156 B	0.33 (0.21)a	0.17 (0.17)	33.50 (2.50)	39.00	4.90 (0.30)	4.20 (0.00)	0.50 (0.34)a	2.33 (1.60)a	0.75 (0.25)
IR60819-34-2	0.67 (0.21)ab	0.83 (0.48)	41.75 (1.60)	39.67 (5.78)	10.25 (2.25)	3.91 (0.49)	1.50 (0.34)ab	10.13 (1.70)ab	0.58 (0.20)
Accession	5.564***		2.774ns [3,17]		0.846ns [3,17]		5.787***	6.539***	0.495ns [3,13]
Sex	0.604ns		0.001ns		12.488***				
AxS	0.411ns		0.504ns		1.351ns				
IR 84714 H	1.17 (0.48)	1.17 (0.48)	37.79 (3.33)	38.54 (1.51)	8.18 (1.14)	4.60 (0.76)	2.33 (0.67)	14.37 (4.44)	0.48 (0.16)
IR 80559 A	0.33 (0.21)	1.00 (0.37)	36.50 (2.50)	35.13 (2.44)	6.25 (3.65)	4.74 (0.52)	1.33 (0.33)	6.58 (2.28)	0.30 (0.20)
IR 80559 B	0.33 (0.21)	0.83 (0.31)	33.00 (3.00)	37.50 (2.33)	10.65 (1.75)	3.35 (0.50)	1.17 (0.31)	6.45 (2.13)	0.30 (0.20)
IR60819-34-2 R	0.50 (0.34)	1.00 (0.52)	41.25 (6.75)	35.83 (1.59)	8.73 (0.33)	5.07 (0.32)	1.50 (0.56)	9.35 (3.47)	0.33 (0.24)
Accession	0.929ns		0.603ns [3,17]		0.685ns [3,17]		1.111ns	1.332ns	0.199ns [3,15]
Sex	2.404ns		0.033ns		24.715***				
AxS	0.288ns		0.860ns		2.123ns				
IR 85471 H	0.83 (0.31)	0.50 (0.22)	40.38 (3.51)	35.33 (1.67)	7.905 (0.45)	4.43 (0.70)	1.33 (0.49)	9.17 (3.93)	0.67 (0.12)
IR 80564 A	0.17 (0.17)	0.50 (0.34)	41.00	32.00 (0.00)	13.10 (0.00)	4.18 (0.63)	0.67 (0.33)	4.17 (2.17)	0.33 (0.33)
IR 80564 B	0.50 (0.34)	2.00 (0.52)	37.25 (1.25)	35.60 (1.77)	9.33 (0.52)	4.57 (0.47)	2.50 (0.72)	12.83 (4.02)	0.12 (0.08)
IR60819-34-2 R	0.50 (0.34)	1.00 (0.52)	41.25 (6.75)	35.83 (1.59)	8.73 (0.33)	5.07 (0.32)	1.50 (0.56)	9.35 (3.47)	0.33 (0.24)
Accession	2.175ns		0.211ns [3,15]		2.820ns [3,15]		1.931ns	1.098ns	1.835ns [3,13]
Sex	3.789ns		5.074*		85.837***				
AxS	2.175ns		0.368ns		3.715*				

IR 81954 H	0.83 (0.40)	1.17 (0.60)	40.50 (2.02)	37.11 (1.06)	8.65 (1.16)	4.23 (0.11)	2.00 (0.52)	11.80 (2.67)	0.53 (0.23)
IR 70369 A	0.17 (0.17)	1.17 (0.40)	41.00	37.63 (4.38)	20.10 (0.00)	4.28 (0.63)	1.33 (0.49)	8.37 (4.87)	0.08 (0.08)
IR 70369 B	0.17 (0.17)	0.17 (0.17)	37.00	31.00	8.80 (0.00)	3.60 (0.00)	0.33 (0.21)	2.07 (1.47)	0.50 (0.50)
IR72889-46-3-2-1 R	0.67 (0.21)	0.50 (0.22)	36.50 (0.65)	34.67 (5.17)	8.18 (1.71)	4.60 (1.01)	1.17 (0.40)	7.75 (2.99)	0.63 (0.13)
Accession	2.183ns		0.637ns [3,12]		6.623** [3,12]		2.623ns	1.468ns	1.424ns [3,11]
Sex	1.581ns		1.329ns		41.724***				
AxS	1.237ns		0.067ns		6.748**				
IR 80637 H	0.83 (0.31)	0.67 (0.42)	37.63 (2.97)	36.75 (0.75)	10.25 (1.60)	3.78 (1.13)	1.50 (0.56)	10.90 (4.03)	0.70 (0.20)
IR 73328 A	0.33 (0.21)	0.50 (0.22)	38.50 (4.50)	37.00 (0.58)	9.50 (1.70)	2.67 (0.37)	0.83 (0.40)	4.50 (2.54)	0.33 (0.17)
IR 73328 B	0.17 (0.17)	1.67 (0.49)	42.00	34.23 (1.63)	8.20 (0.00)	4.66 (0.58)	1.83 (0.48)	8.63 (2.28)	0.10 (0.10)
IR73013-95-1-3-2 R	0.33 (0.21)	0.33 (0.21)	39.00 (6.00)	33.00 (0.00)	9.20 (4.40)	3.80 (1.00)	0.67 (0.33)	4.33 (2.26)	0.50 (0.29)
Accession	1.667ns		0.158ns [3,13]		0.114ns [3,13]		1.485ns	1.150ns	2.263ns [3,12]
Sex	3.092ns		3.263ns		19.459***				
AxS	3.193ns		0.559ns		0.311ns				
IR 82385 H	0.17 (0.17)	1.50 (0.56)	40.00ab	36.30 (2.06)	7.80 (0.00)	3.34 (0.56)	1.67 (0.61)	6.77 (3.08)	0.07 (0.07)
IR 79125 A	0.67 (0.33)	0.50 (0.34)	34.00 (2.00)a	29.75 (1.25)	9.27 (0.79)	3.33 (0.98)	1.17 (0.31)	7.93 (3.02)	0.60 (0.24)
IR 79125 B	0.33 (0.21)	0.17 (0.17)	43.50 (6.50)b	39.00	8.40 (3.00)	3.30 (0.00)	0.50 (0.22)	3.35 (1.85)	0.67 (0.33)
IR73717-46-1-3-3 R	0.50 (0.22)	0.67 (0.21)	37.67 (2.73)ab	41.25 (1.70)	9.47 (3.11)	4.55 (1.41)	1.17 (0.31)	7.77 (3.55)	0.40 (0.19)
Accession	1.241ns		3.408* [3,13]		0.220ns [3,13]		1.486ns	0.586ns	1.701ns [3,14]
Sex	1.842ns		0.990ns		11.442***				
AxS	2.744ns		0.987ns		0.046ns				
IR 82363 H	0.83 (0.31)	1.17 (0.48)	39.38 (1.14)b	34.92 (2.02)	10.43 (1.74)	3.49 (0.49)	2.00 (0.52)b	12.65 (3.73)ab	0.50 (0.18)
IR 68897 A	0.33 (0.21)	1.50 (0.34)	32.50 (1.50)ab	35.60 (0.78)	7.25 (3.25)	3.93 (0.66)	1.83 (0.31)ab	7.92 (0.72)ab	0.22 (0.16)
IR 68897 B	0.00 (0.00)	0.50 (0.22)	31.00a	31.67 (1.20)	10.50 (0.00)	4.30 (0.55)	0.50 (0.22)a	2.15 (0.99)a	0.00 (0.00)
SRT 3 R	1.17 (0.48)	1.00 (0.26)	37.17 (1.42)ab	34.00 (1.52)	11.39 (2.02)	5.03 (0.94)	2.17 (0.31)b	18.49 (4.62)b	0.44 (0.16)
Accession	2.808ns		3.496* [3,20]		1.171ns [3,20]		4.597*	5.443**	1.427ns [3,17]
Sex	4.060ns		0.623ns		25.387***				
AxS	1.465ns		2.377ns		0.602ns				
IR 80814 H	0.67 (0.49)	0.83 (0.48)	37.17 (2.17)	37.67 (1.20)	10.55 (2.55)	3.88 (0.84)	1.50 (0.67)	9.43 (4.82)	0.31 (0.24)
IR 70369 A	0.17 (0.17)	1.17 (0.40)	41.00	37.63 (4.38)	20.10 (0.00)	4.28 (0.63)	1.33 (0.49)	8.37 (4.87)	0.08 (0.08)
IR 70369 B	0.17 (0.17)	0.17 (0.17)	37.00	31.00	8.80 (0.00)	3.60 (0.00)	0.33 (0.21)	2.07 (1.47)	0.50 (0.50)

IR69712-154-2-3-1-3 R	0.67 (0.33)	0.50 (0.34)	33.00 (0.58)	35.25 (3.25)	9.23 (1.19)	4.18 (0.48)	1.17 (0.65)	8.35 (4.52)	0.67 (0.17)
Accession	1.137ns		0.754ns [3,9]		7.228** [3,9]		0.921ns	0.634ns	1.342ns [
Sex	1.059ns		0.295ns		63.892***				
AxS	1.137ns		0.352ns		6.360*				
IR 81955 H	0.33 (0.21)	0.33 (0.21)	37.50 (1.50)	41.50 (6.50)	12.15 (0.25)	1.95 (0.05)	0.67 (0.21)	4.70 (2.38)	0.50 (0.29)
IR 70369 A	0.17 (0.17)	1.17 (0.40)	41.00	37.63 (4.38)	20.10 (0.00)	4.28 (0.63)	1.33 (0.49)	8.37 (4.87)	0.08 (0.08)
IR 70369 B	0.17 (0.17)	0.17 (0.17)	37.00	31.00 ()	8.80 (0.00)	3.60 (0.00)	0.33 (0.21)	2.07 (1.47)	0.50 (0.50)
IR72998-93-3-3-2 R	0.67 (0.33)	1.00 (0.52)	36.00 (2.08)	37.11 (1.46)	12.40 (0.84)	4.58 (1.32)	1.67 (0.56)	13.15 (5.44)	0.45 (0.23)
Accession	2.083ns		0.471ns [3,9]		9.677ns [3,9]		2.299ns	1.484ns	0.644ns [3,11]
Sex	2.500ns		0.093ns		143.471***				
AxS	1.250ns		0.376ns		7.010**				
IR 81956 H	0.83 (0.31)	0.17 (0.17)	40.63 (1.82)	44.00 ()	9.41 (1.50)	3.60 (0.00)	1.00 (0.26)	8.22 (2.47)	0.80 (0.20)
IR 70369 A	0.17 (0.17)	1.17 (0.40)	41.00	37.63 (4.38)	20.10 (0.00)	4.28 (0.63)	1.33 (0.49)	8.37 (4.87)	0.08 (0.08)
IR 70369 B	0.17 (0.17)	0.17 (0.17)	37.00	31.00	8.80 (0.00)	3.60 (0.00)	0.33 (0.21)	2.07 (1.47)	0.50 (0.50)
IR73003-73-2-3-2 R	0.17 (0.17)	0.67 (0.33)	34.00	32.33 (0.33)	7.20 (0.00)	2.48 (0.68)	0.83 (0.31)	3.08 (1.42)	0.25 (0.25)
Accession	1.374ns		1.627ns [3,8]		7.649** [3,8]		1.543ns	1.286ns	2.241ns [3,11]
Sex	1.374ns		0.297ns		39.789***				
AxS	4.011*		0.315ns		5.010*				
IR 81958 H	0.33 (0.21)	0.67 (0.33)	37.00 (4.00)	33.00 (0.58)	8.00 (3.20)	4.13 (1.04)	1.00 (0.52)	5.65 (3.52)	0.28 (0.15)
IR 70369 A	0.17 (0.17)	1.17 (0.40)	41.00	37.63 (4.38)	20.10 (0.00)	4.28 (0.63)	1.33 (0.49)	8.37 (4.87)	0.08 (0.08)
IR 70369 B	0.17 (0.17)	0.17 (0.17)	37.00	31.00	8.80 (0.00)	3.60 (0.00)	0.33 (0.21)	2.07 (1.47)	0.50 (0.50)
IR73013-95-1-3-2 R	0.33 (0.21)	0.33 (0.21)	39.00 (6.00)	33.00 (0.00)	9.20 (4.40)	3.80 (1.00)	0.67 (0.33)	4.33 (2.26)	0.50 (0.29)
Accession	1.515ns		0.378ns [3,8]		3.240ns [3,8]		1.111ns	0.626ns	0.855ns [3,8]
Sex	3.636ns		1.755ns		19.961***				
AxS	1.818ns		0.036ns		2.870ns				
IR 80228 H	0.17 (0.17)	1.00 (0.45)	46.00	35.75 (1.75)	5.60 (0.00)	4.59 (1.12)	1.17 (0.40)	5.35 (1.75)	0.20 (0.20)
IR 70369 A	0.17 (0.17)	1.17 (0.40)	41.00	37.63 (4.38)	20.10 (0.00)	4.28 (0.63)	1.33 (0.49)	8.37 (4.87)	0.08 (0.08)
IR 70369 B	0.17 (0.17)	0.17 (0.17)	37.00	31.00	8.80 (0.00)	3.60 (0.00)	0.33 (0.21)	2.07 (1.47)	0.50 (0.50)
IR73885-1-4-3-2-1-6 R	0.33 (0.21)	0.17 (0.17)	36.50 (2.50)	33.00	7.05 (2.15)	4.20 (0.00)	0.50 (0.22)	3.05 (1.53)	0.67 (0.33)
Accession	1.769ns		0.760ns [3,7]		8.248* [3,7]		1.926ns	1.006ns	1.171ns [3,10]
Sex	5.102*		2.229ns		25.000***				
AxS	2.517ns		0.201ns		8.339**				

IR 81949 H	0.33 (0.21)	1.17 (0.75)	41.50 (6.50)	35.25 (0.75)	10.25 (3.05)	4.80 (0.00)	1.50 (0.85)	9.02 (5.25)	0.40 (0.31)
IR 68897 A	0.33 (0.21)	1.50 (0.34)	32.50 (1.50)	35.60 (0.78)	7.25 (3.25)	3.93 (0.66)	1.83 (0.31)	7.92 (0.72)	0.22 (0.16)
IR 68897 B	0.00 (0.00)	0.50 (0.22)	31.00	31.67 (1.20)	10.50 (0.00)	4.30 (0.55)	0.50 (0.22)	2.15 (0.99)	0.00 (0.00)
IR73013-95-1-3-2 R	0.33 (0.21)	0.33 (0.21)	39.00 (6.00)	33.00 (0.00)	9.20 (4.40)	3.80 (1.00)	0.67 (0.33)	4.33 (2.26)	0.50 (0.29)
Accession	1.830ns		1.903ns [3,11]		0.446ns [3,11]		1.705ns	1.209ns	0.864ns [3,11]
Sex	6.02*		1.073ns		12.426***				
AxS	1.094ns		1.529ns		0.222ns				
IR 86167 H	0.83 (0.54)	0.67 (0.33)	21.17 (14.84)	36.33 (1.20)	7.20 (0.70)	5.03 (1.13)	1.50 (0.67)	9.27 (4.24)	0.38 (0.24)
IR80156A	0.50 (0.34)	0.17 (0.17)	35.25 (5.25)	30.00	7.38 (0.83)	6.10 (0.00)	0.67 (0.42)	4.57 (2.89)	0.75 (0.25)
IR80156B	0.50 (0.22)	0.67 (0.33)	41.00 (2.08)	47.33 (6.36)	9.50 (0.72)	3.50 (1.30)	1.17 (0.40)	6.72 (2.92)	0.47 (0.23)
IR73013-95-1-3-2 R	0.33 (0.21)	0.33 (0.21)	39.00 (6.00)	33.00 (0.00)	9.20 (4.40)	3.80 (1.00)	0.67 (0.33)	4.33 (2.26)	0.50 (0.29)
Accession	0.833ns		2.689ns [3,10]		0.041ns [3,9]		0.273ns	0.512ns	0.273ns
Sex	0.139ns		0.311ns		8.345*				
AxS	0.231ns		1.242ns		0.820ns				

1: Numbers are means (SEM)(N=6); ns = P > 0.05, * = P < 001; ** = P < 0.01, *** = P < 0.005; lowercase letters indicate homogenous line groups (Tukey: P > 0.05). 2: DF = 3,40 for accession and interaction between accession and sex, 1,40 for sex, unless indicated in square brackets; 3: DF = 3,20 for accession unless indicated in square brackets; 4: Note that some of the results for YSB biomass have been summarized by plant-type and presented in a related paper by Horgan et al (2016); the data and information by accession has not been previously published.

Table S7: Responses by YSB to 15 hybrid rice varieties indicating potential sources of resistance/susceptibility based on heterosis or heterobeltiosis with parental lines. Information related to parental lines is presented in Table S6.

Accession	Number of Individuals per Plant[1,2,4]		Development Time (Days) [1,2,4]		Adult Weight (mg) [1,2]		Total number of Individuals[1,3]	Total Dry Weight (mg) [1,3,4]	Females (Proportion) [1,3]
	Female	Male	Female	Male	Female	Male			
IR82396H	0.67 (0.33)	0.67 (0.33)	37.17 (1.96)	38.33 (3.48)	9.55 (1.28)	5.42 (0.49)	1.33 (0.49)	9.72 (3.68)	0.46 (0.21)
IR 86167 H	0.83 (0.54)	0.67 (0.33)	21.17 (14.84)	36.33 (1.20)	7.20 (0.70)	5.03 (1.13)	1.50 (0.67)	9.27 (4.24)	0.38 (0.24)
IR 81954 H	0.83 (0.40)	1.17 (0.60)	40.50 (2.02)	37.11 (1.06)	8.65 (1.16)	4.23 (0.11)	2.00 (0.52)	11.80 (2.67)	0.53 (0.23)
IR 80814 H	0.67 (0.49)	0.83 (0.48)	37.17 (2.17)	37.67 (1.20)	10.55 (2.55)	3.88 (0.84)	1.50 (0.67)	9.43 (4.82)	0.31 (0.24)
IR 81955 H	0.33 (0.21)	0.33 (0.21)	37.50 (1.50)	41.50 (6.50)	12.15 (0.25)	1.95 (0.05)	0.67 (0.21)	4.70 (2.38)	0.50 (0.29)
IR 81956 H	0.83 (0.31)	0.17 (0.17)	40.63 (1.82)	44.00 ()	9.41 (1.50)	3.60 (0.00)	1.00 (0.26)	8.22 (2.47)	0.80 (0.20)
IR 81958 H	0.33 (0.21)	0.67 (0.33)	37.00 (4.00)	33.00 (0.58)	8.00 (3.20)	4.13 (1.04)	1.00 (0.52)	5.65 (3.52)	0.28 (0.15)
IR 80228 H	0.17 (0.17)	1.00 (0.45)	46.00	35.75 (1.75)	5.60 (0.00)	4.59 (1.12)	1.17 (0.40)	5.35 (1.75)	0.20 (0.20)
IR 80637 H	0.83 (0.31)	0.67 (0.42)	37.63 (2.97)	36.75 (0.75)	10.25 (1.60)	3.78 (1.13)	1.50 (0.56)	10.90 (4.03)	0.70 (0.20)
IR 81949 H	0.33 (0.21)	1.17 (0.75)	41.50 (6.50)	35.25 (0.75)	10.25 (3.05)	4.80 (0.00)	1.50 (0.85)	9.02 (5.25)	0.40 (0.31)
IR 82363 H	0.83 (0.31)	1.17 (0.48)	39.38 (1.14)	34.92 (2.02)	10.43 (1.74)	3.49 (0.49)	2.00 (0.52)	12.65 (3.73)	0.50 (0.18)
IR 82391 H	1.33 (0.42)	2.00 (0.68)	35.40 (0.60)	34.98 (2.02)	9.38 (0.86)	4.12 (0.30)	3.33 (0.80)	20.73 (4.98)	0.39 (0.11)
IR 84714 H	1.17 (0.48)	1.17 (0.48)	37.79 (3.33)	38.54 (1.51)	8.18 (1.14)	4.60 (0.76)	2.33 (0.67)	14.37 (4.44)	0.48 (0.16)
IR 85471 H	0.83 (0.31)	0.50 (0.22)	40.38 (3.51)	35.33 (1.67)	7.905 (0.45)	4.43 (0.70)	1.33 (0.49)	9.17 (3.93)	0.67 (0.12)
IR 82385 H	0.17 (0.17)	1.50 (0.56)	40.00	36.30 (2.06)	7.80 (0.00)	3.34 (0.56)	1.67 (0.61)	6.77 (3.08)	0.07 (0.07)
F-Accession (A)	1.247ns		1.630ns [14,81]		0.456ns [14,61]		1.258ns	1.120ns	1.015ns [14,53]
F-Sex (S)	2.447ns		0.565ns [1,81]		103.170*** [1,61]				
F-AxS	0.806ns		1.491ns [14,81]		1.244ns [14,61]				

1: Numbers are means (SEM)(N=6); ns = P > 0.05, *** = P < 0.005; 2: DF = 14,150 for accession and interaction between accession and sex, 1,150 for sex, unless indicated in square brackets; 3: DF = 14,75 for accession unless indicated in square brackets; 4: Blue = heterosis

Table S8: Oviposition by YSB in choice experiments with hybrids and associated parental lines.

Variety	Egg masses (per plant)[1]	Eggs (per plant)[1]	Plant biomass (dry mg)[2]	Tiller number (per plant)[2]
IR82396 H	1.66 (0.21)	120.00 (21.61)	0.42 (0.02)	1.50 (0.34)
IR80156 A	3.00 (0.86)	160.66 (54.46)	0.57 (0.17)	1.83 (0.48)
IR80156 B	2.16 (0.40)	102.50 (29.44)	0.41 (0.05)	1.33 (0.21)
IR46R	2.00 (0.52)	102.83 (33.39)	0.31 (0.02)	1.00 (0.00)
F-line	0.426ns	0.080ns	1.246ns	1.238ns
F-biomass	0.669ns	1.108ns		
F-tillers	0.188ns	0.360ns		
IR82391 H	1.50 (0.34)	65.00 (10.90)ab	0.33 (0.02)ab	1.00 (0.00)
IR79156 A	1.66 (0.33)	109.66 (25.93)b	0.30 (0.02)ab	1.00 (0.00)
IR79156 B	0.33 (0.21)	7.66 (5.75)a	0.28 (0.02)a	1.16 (0.16)
IR60819-34-2 R	1.83 (0.70)	59.16 (21.01)ab	0.38 (0.03)b	1.16 (0.16)
F-line	1.090ns	3.614*	4.220*	0.625ns
F-biomass	0.001ns	1.237ns		
F-tillers	1.315ns	2.693ns		
IR84714 H	4.00 (0.63)b	209.83 (49.93)b	0.61 (0.14)	2.16 (0.40)
IR80559 A	1.83 (0.75)ab	84.66 (45.36)ab	0.60 (0.11)	2.33 (0.21)
IR80559 B	1.50 (0.62)a	45.66 (26.54)a	0.59 (0.16)	2.16 (0.40)
IR60819-34-2 R	2.33 (0.56)ab	135.66 (32.71)ab	0.46 (0.03)	1.33 (0.21)
F-line	4.586**	3.874*	0.373ns	1.982ns
F-biomass	1.668ns	0.476ns		
F-tillers	4.164*	2.228ns		
IR85471 H	1.00 (0.36)	55.66 (23.05)	0.35 (0.02)	1.16 (0.16)
IR80564 A	0.50 (0.34)	15.83 (10.15)	0.30 (0.01)	1.16 (0.16)
IR80564 B	2.33 (1.02)	100.83 (53.88)	0.44 (0.14)	1.33 (0.33)
IR60819-34-2 R	1.50 (0.43)	87.83 (33.98)	0.41 (0.03)	1.50 (0.22)
F-line	0.815ns	0.459ns	0.649ns	0.470ns
F-biomass	11.703***	9.998***		
F-tillers	1.461ns	0.747ns		
IR81954 H	1.00 (0.26)	144.50 (70.08)	1.59 (0.09)	3.33 (0.21)
IR70369 A	2.33 (0.92)	147.83 (61.35)	1.48 (0.11)	3.00 (0.00)
IR70369 B	2.50 (0.67)	137.66 (43.81)	1.48 (0.08)	2.66 (0.21)

IR72889-46-3-2-1 R	1.83 (0.31)	102.50 (22.81)	1.25 (0.07)	3.33 (0.33)
F-line	1.358ns	0.596ns	2.622ns	2.037ns
F-biomass	0.584ns	1.263ns		
F-tillers	0.786ns	0.264ns		
IR80637 H	3.00 (0.63)	117.50 (34.91)	1.34 (0.06)	3.66 (0.33)
IR73328 A	2.50 (0.92)	120.66 (38.05)	1.30 (0.06)	3.16 (0.31)
IR73328 B	2.66 (0.56)	133.50 (38.21)	1.32 (0.05)	3.50 (0.22)
IR73013-95-1-3-2 R	2.66 (0.56)	152.00 (46.55)	1.24 (0.07)	4.16 (0.16)
F-line	0.045ns	0.043ns	0.472ns	2.451ns
F-biomass	0.196ns	0.371ns		
F-tillers	0.193ns	0.119ns		
IR82385 H	2.00 (0.58)	105.83 (33.77)	1.08 (0.18)	3.00 (0.25)
IR79125 A	2.00 (0.58)	92.33 (27.86)	1.48 (0.10)	3.33 (0.21)
IR79125 B	2.66 (0.67)	112.33 (30.51)	1.38 (0.12)	3.50 (0.22)
IR73717-46-1-3-3 R	2.83 (0.94)	100.50 (29.07)	1.03 (0.11)	2.66 (0.21)
F-line	0.884ns	0.667ns	2.785ns	2.658ns
F-biomass	1.442ns	0.116ns		
F-tillers	0.032ns	0.523ns		
IR 82363 H	2.33 (0.61)	95.66 (33.80)	1.12 (0.05)	3.00 (0.26)
IR68897 A	1.50 (0.85)	66.50 (33.53)	1.06 (0.08)	2.66 (0.21)
IR68897 B	1.66 (0.56)	85.66 (27.96)	1.10 (0.05)	2.66 (0.21)
SRT 3 R	1.66 (0.49)	83.83 (31.83)	1.09 (0.05)	2.50 (0.22)
F-line	0.723ns	0.402ns	0.258ns	0.856ns
F-biomass	0.242ns	0.026ns		
F-tillers	0.048ns	0.019ns		

1: Numbers are means (SEM) (N = 6); ns = P > 0.05, * = P < 0.05, ** = P < 0.01, *** = P < 0.001; lowercase numbers indicate homogenous variety groups (Tukey P > 0.05); 2: Degrees of freedom for masses and eggs = 3,18 for accessions/lines, 1,18 for covariates; lowercase numbers indicate homogenous variety groups (Tukey P > 0.05); 3: Degrees of freedom for plant biomass and tiller number = 3,20;

Table S9: Plant growth parameters in field plots at IRRI. Results are summarized in Table S10 and Figure 2.

Accession	Sampling Time (DAT)	Plant Height (cm)[1]	Total Tiller Number[1,2]	Leaf Dry Weigh (g) [1]	Stem Dry Weight (g) [1]	Total Plant Dry Weight (g) [1,2]	SPAD-value[1]
IR82396H	28	49.40 (1.24)	16.00 (0.73)	1.24 (0.09)	2.01 (0.14)	3.25 (0.22)	39.04 (0.47)
IR80156A	28	44.30 (0.98)	11.80 (0.94)	0.66 (0.05)	1.25 (0.14)	1.90 (0.19)	36.46 (0.39)
IR80156B	28	46.50 (0.72)	11.20 (0.83)	0.59 (0.05)	0.95 (0.10)	1.54 (0.14)	37.22 (0.26)
IR46R	28	46.60 (0.81)	15.80 (1.18)	0.18 (0.08)	1.73 (0.13)	2.91 (0.21)	40.28 (0.48)
IR82396H	42	72.80 (1.63)	46.00 (2.85)	1.06 (0.42)	6.95 (0.39)	14.02 (0.80)	38.94 (0.42)
IR80156A	42	67.30 (1.40)	43.40 (1.78)	1.85 (0.25)	6.77 (0.30)	12.62 (0.52)	36.76 (0.40)
IR80156B	42	64.60 (1.48)	41.20 (1.61)	1.85 (0.31)	5.12 (0.29)	9.97 (0.60)	36.92 (0.35)
IR46R	42	62.80 (1.32)	41.20 (2.17)	1.57 (0.32)	6.64 (0.50)	12.21 (0.77)	40.20 (0.39)
IR82396H	56	88.90 (1.18)	28.40 (1.54)	1.02 (0.73)	17.86 (0.86)	30.89 (1.58)	36.44 (0.62)
IR80156A	56	77.12 (1.49)	27.40 (1.51)	1.87 (0.44)	13.15 (0.72)	22.02 (1.13)	33.80 (0.34)
IR80156B	56	77.18 (1.52)	33.60 (1.87)	1.01 (0.66)	14.43 (1.14)	24.45 (1.78)	34.94 (0.21)
IR46R	56	83.58 (1.02)	30.60 (2.16)	1.46 (0.58)	16.62 (0.67)	30.08 (1.21)	37.78 (0.33)
IR82396H	70	102.20 (1.35)	44.40 (1.74)	1.04 (0.30)	33.80 (0.74)	52.84 (1.00)	42.12 (0.31)
IR80156A	70	97.50 (2.55)	48.00 (2.15)	2.43 (0.91)	31.91 (1.63)	46.33 (2.45)	40.50 (0.34)
IR80156B	70	96.40 (1.45)	40.20 (2.06)	1.41 (0.59)	24.37 (1.35)	36.78 (1.90)	39.60 (0.20)
IR46R	70	96.75 (1.84)	47.00 (0.96)	1.09 (1.26)	34.22 (1.12)	58.31 (2.35)	40.26 (0.54)
IR82396H	84	114.16 (1.12)	26.20 (1.37)	1.88 (0.42)	22.90 (0.64)	36.78 (0.91)	42.15 (0.55)
IR80156A	84	97.92 (1.91)	35.20 (2.77)	1.58 (0.51)	29.01 (0.88)	43.58 (1.18)	41.22 (0.21)
IR80156B	84	100.30 (2.28)	25.00 (1.39)	2.84 (0.58)	17.57 (0.81)	27.41 (1.34)	40.46 (0.45)
IR46R	84	113.50 (0.85)	27.60 (1.85)	0.16 (0.99)	24.68 (0.94)	40.83 (1.86)	42.74 (0.18)
IR82396H	98	114.22 (0.98)	31.80 (1.80)	0.67 (1.55)	27.18 (2.37)	42.85 (3.90)B	38.22 (0.56)
IR80156A	112	92.68 (0.64)	64.10 (1.10)	0.24 (0.54)	36.55 (0.72)	53.78 (1.20)AB	38.82 (0.16)
IR80156B	93	103.69 (1.49)	28.80 (1.80)	1.18 (0.63)	18.27 (0.91)	28.44 (1.54)A	37.90 (0.50)
IR46R	112	113.75 (0.93)	29.50 (1.50)	0.12 (1.03)	36.49 (2.10)	55.61 (3.12)AB	38.28 (0.80)
Time			45.323***			175.215***	2.844*
Time x Accession			4.479***			1.444ns	1.070ns
Within contrasts			161.804***Q			7.6357***	
Accession			2.715ns			4.880*	1.417ns
IR 82391 H	28	46.60 (1.32)	15.60 (1.35)	1.02 (0.10)	1.78 (0.21)	2.80 (0.31)	38.24 (0.38)
IR 79156 A	28	44.40 (0.88)	22.00 (1.63)	0.02 (0.09)	2.17 (0.18)	3.19 (0.27)	38.60 (0.21)

IR 79156 B	28	43.50 (1.05)	14.00 (0.73)	1.68 (0.08)	1.13 (0.10)	1.82 (0.18)	39.02 (0.25)
IR60819-34-2	28	46.60 (0.86)	13.00 (1.22)	0.79 (0.05)	1.50 (0.14)	2.30 (0.18)	41.34 (0.52)
IR 82391 H	42	72.20 (0.72)	44.60 (1.80)	0.52 (0.27)	7.68 (0.46)	14.20 (0.68)	39.62 (0.48)
IR 79156 A	42	62.00 (2.11)	54.40 (1.82)	2.05 (0.29)	6.25 (0.46)	12.30 (0.75)	38.28 (0.62)
IR 79156 B	42	64.70 (0.93)	40.40 (1.65)	0.76 (0.23)	5.34 (0.30)	10.10 (0.49)	36.88 (0.47)
IR60819-34-2	42	72.50 (1.07)	35.40 (0.77)	1.53 (0.23)	6.73 (0.50)	12.26 (0.72)	41.26 (0.56)
IR 82391 H	56	90.44 (0.78)	31.00 (1.76)	0.09 (0.58)	16.20 (0.62)	27.29 (1.05)	36.92 (0.85)
IR 79156 A	56	85.05 (1.45)	38.60 (1.39)	1.07 (0.36)	15.15 (0.68)	27.22 (0.99)	36.30 (0.51)
IR 79156 B	56	80.80 (1.23)	29.80 (2.89)	1.55 (0.73)	12.67 (0.90)	23.22 (1.62)	35.92 (0.56)
IR60819-34-2	56	94.17 (1.23)	33.20 (2.16)	1.82 (0.62)	15.82 (0.57)	28.64 (1.14)	39.62 (0.33)
IR 82391 H	70	111.75 (2.49)	41.60 (1.53)	2.58 (0.88)	34.06 (1.35)	50.63 (2.21)	42.88 (0.27)
IR 79156 A	70	107.20 (1.57)	47.20 (1.66)	1.98 (0.34)	31.77 (1.21)	48.75 (1.48)	39.38 (0.35)
IR 79156 B	70	110.25 (2.39)	49.00 (1.98)	2.74 (0.61)	30.18 (1.07)	47.91 (1.63)	38.22 (0.41)
IR60819-34-2	70	98.80 (1.77)	38.80 (1.12)	1.37 (0.91)	32.93 (1.28)	51.30 (2.10)	42.88 (0.36)
IR 82391 H	84	107.86 (2.11)	24.80 (0.91)	2.57 (0.61)	19.03 (0.50)	30.60 (1.03)	40.92 (0.36)
IR 79156 A	84	110.34 (1.80)	25.20 (1.52)	1.00 (0.73)	24.13 (1.34)	36.13 (2.01)	40.20 (0.55)
IR 79156 B	84	115.26 (0.81)	21.80 (0.74)	0.67 (0.43)	20.16 (0.77)	32.84 (1.11)	39.10 (0.44)
IR60819-34-2	84	102.76 (0.51)	20.40 (1.51)	0.32 (0.48)	19.33 (0.42)	31.65 (0.80)	46.22 (0.70)
IR 82391 H	93	112.98 (2.02)	25.20 (1.80)AB	2.57 (0.71)	19.78 (1.30)	30.36 (1.98)	39.98 (0.59)B
IR 79156 A	98	111.99 (1.23)	33.80 (1.05)B	1.66 (0.49)	25.68 (0.89)	39.34 (1.29)	37.46 (0.31)AB
IR 79156 B	98	113.38 (0.82)	33.70 (2.01)AB	0.34 (0.57)	26.60 (0.87)	41.94 (1.42)	38.36 (0.57)A
IR60819-34-2	93	107.34 (0.25)	24.60 (0.93)A	0.89 (0.53)	24.79 (0.78)	39.67 (1.15)	42.46 (0.54)C
Time			44.339***			144.167***	7.485***
Time x Accession			1.070ns			1.761ns	0.879ns
Within contrasts			72.730***Q			649.022***L	12.873***C
Accession			4.858*			1.905ns	27.266***
IR 84714 H	28	47.70 (0.99)	17.80 (1.19)	0.04 (0.08)	1.97 (0.14)	3.00 (0.21)	40.56 (0.28)
IR 80559 A	28	46.60 (0.90)	14.40 (1.37)	0.85 (0.10)	1.46 (0.21)	2.30 (0.31)	36.44 (0.27)
IR 80559 B	28	45.90 (0.94)	22.20 (1.28)	0.08 (0.08)	1.96 (0.26)	3.03 (0.34)	37.12 (0.42)
IR60819-34-2 R	28	38.20 (0.57)	8.00 (0.55)	0.36 (0.03)	0.64 (0.06)	1.00 (0.09)	38.68 (0.29)
IR 84714 H	42	74.10 (0.91)	42.00 (2.55)	0.44 (0.26)	7.62 (0.32)	14.06 (0.56)	41.78 (0.51)
IR 80559 A	42	70.80 (0.76)	46.60 (1.27)	0.56 (0.17)	7.55 (0.22)	14.11 (0.39)	38.62 (0.27)
IR 80559 B	42	65.90 (1.03)	51.00 (3.21)	1.47 (0.50)	7.41 (0.62)	13.87 (1.07)	38.78 (0.60)
IR60819-34-2 R	42	64.20 (0.91)	31.00 (0.81)	0.69 (0.13)	4.47 (0.33)	8.17 (0.45)	41.20 (0.37)

IR 84714 H	56	85.00 (0.75)	28.20 (2.13)	0.58 (0.44)	16.39 (0.79)	26.97 (1.20)	38.12 (0.51)
IR 80559 A	56	83.71 (0.96)	31.00 (1.05)	0.66 (0.39)	14.46 (0.32)	26.12 (0.69)	33.92 (0.29)
IR 80559 B	56	79.75 (0.59)	30.80 (1.49)	0.81 (0.78)	12.79 (0.96)	23.60 (1.64)	35.78 (0.62)
IR60819-34-2 R	56	86.88 (1.13)	28.60 (1.25)	1.56 (0.66)	13.61 (0.79)	25.18 (1.44)	36.82 (0.38)
IR 84714 H	70	102.00 (0.51)	33.60 (1.45)	0.73 (0.52)	29.44 (0.91)	42.17 (1.41)	44.84 (0.26)
IR 80559 A	70	104.20 (0.84)	43.00 (1.19)	0.69 (0.26)	29.04 (1.04)	44.73 (1.23)	41.04 (0.34)
IR 80559 B	70	98.75 (3.53)	44.80 (1.14)	3.44 (0.67)	28.22 (0.76)	43.66 (1.36)	41.38 (0.41)
IR60819-34-2 R	70	102.00 (0.69)	45.00 (1.61)	0.16 (0.82)	34.52 (1.14)	55.69 (1.93)	44.32 (0.34)
IR 84714 H	84	100.96 (1.76)	20.00 (1.03)	1.40 (0.40)	16.21 (0.52)	25.62 (0.90)	43.80 (0.38)
IR 80559 A	84	102.24 (1.39)	28.60 (1.39)	1.86 (0.41)	27.97 (1.31)	41.83 (1.70)	40.40 (0.14)
IR 80559 B	84	106.68 (1.30)	25.60 (0.76)	1.72 (0.71)	18.60 (0.71)	30.32 (1.39)	42.58 (1.14)
IR60819-34-2 R	84	105.42 (1.15)	21.60 (1.65)	1.60 (0.11)	20.76 (0.35)	33.36 (0.36)	46.60 (0.32)
IR 84714 H	93	104.70 (1.01)	26.60 (0.76)	1.55 (0.19)	21.76 (0.59)	32.31 (0.75)	42.20 (0.30)B
IR 80559 A	112	99.62 (0.29)	57.70 (2.50)	0.13 (0.30)	45.75 (1.87)	63.88 (2.01)	37.80 (0.25)AB
IR 80559 B	93	106.22 (0.66)	32.00 (1.23)	0.69 (0.54)	23.07 (1.05)	35.76 (1.34)	40.40 (0.31)A
IR60819-34-2 R	98	107.42 (0.66)	22.90 (0.96)	0.63 (0.61)	25.69 (0.77)	39.32 (1.32)	42.32 (0.53)AB
Time			40.496***			214.455***	6.076***
Time x Accession			6.606***			0.835ns	1.447ns
Within contrasts			57.611***C			1368.818***L	10.980***L
Accession			2.676ns			1.973ns	4.338*
IR 85471 H	28	54.10 (0.85)	17.60 (0.98)	0.47 (0.11)	2.23 (0.20)	3.70 (0.30)	38.32 (0.63)
IR 80564 A	28	46.00 (1.10)	13.00 (0.98)	1.78 (0.08)	1.30 (0.13)	2.08 (0.20)	39.44 (0.50)
IR 80564 B	28	45.90 (0.97)	15.00 (1.32)	0.94 (0.10)	1.55 (0.19)	2.48 (0.29)	40.34 (0.63)
IR60819-34-2 R	28	38.20 (0.57)	8.00 (0.55)	0.36 (0.03)	0.64 (0.06)	1.00 (0.09)	38.68 (0.29)
IR 85471 H	42	75.00 (1.21)	34.60 (1.90)	1.49 (0.42)	7.29 (0.46)	13.79 (0.85)	38.78 (0.37)
IR 80564 A	42	64.20 (0.67)	39.20 (1.18)	0.17 (0.28)	6.31 (0.46)	11.48 (0.73)	40.40 (0.43)
IR 80564 B	42	63.60 (1.18)	32.20 (0.68)	1.69 (0.23)	5.67 (0.33)	10.36 (0.53)	40.70 (0.17)
IR60819-34-2 R	42	64.20 (0.91)	31.00 (0.81)	0.69 (0.13)	4.47 (0.33)	8.17 (0.45)	41.20 (0.37)
IR 85471 H	56	91.82 (0.32)	28.20 (1.05)	0.80 (0.31)	18.00 (0.71)	30.80 (0.67)	37.42 (0.39)
IR 80564 A	56	79.03 (1.28)	30.00 (2.87)	1.46 (0.61)	13.83 (0.70)	24.29 (1.27)	38.50 (0.19)
IR 80564 B	56	79.03 (0.78)	28.20 (1.34)	0.53 (0.44)	15.17 (0.45)	26.69 (0.87)	37.94 (0.49)
IR60819-34-2 R	56	86.88 (1.13)	28.60 (1.25)	1.56 (0.66)	13.61 (0.79)	25.18 (1.44)	36.82 (0.38)
IR 85471 H	70	110.25 (0.43)	41.40 (1.20)	0.25 (0.82)	39.12 (1.37)	57.37 (2.18)	41.36 (0.30)
IR 80564 A	70	97.00 (1.67)	33.60 (0.90)	1.41 (0.41)	27.98 (0.79)	41.39 (1.12)	44.40 (0.27)

IR 80564 B	70	100.20 (1.10)	39.00 (1.17)	1.01 (0.63)	31.08 (1.63)	46.09 (2.23)	43.62 (0.31)
IR60819-34-2 R	70	102.00 (0.69)	45.00 (1.61)	0.16 (0.82)	34.52 (1.14)	55.69 (1.93)	44.32 (0.34)
IR 85471 H	84	102.70 (2.03)	20.60 (0.82)	2.20 (0.31)	17.40 (0.34)	28.60 (0.45)	42.04 (0.51)
IR 80564 A	84	94.64 (1.52)	26.00 (1.20)	1.06 (0.46)	27.61 (1.12)	39.67 (1.56)	44.38 (0.44)
IR 80564 B	84	102.22 (1.69)	24.80 (1.22)	1.96 (0.50)	19.35 (0.55)	31.31 (0.99)	45.68 (0.20)
IR60819-34-2 R	84	105.42 (1.15)	21.60 (1.65)	1.60 (0.11)	20.76 (0.35)	33.36 (0.36)	46.60 (0.32)
IR 85471 H	93	108.57 (0.68)	24.20 (1.02)	0.47 (0.64)	19.95 (0.58)	31.43 (1.16)B	40.00 (0.31)A
IR 80564 A	112	95.46 (0.76)	65.70 (3.67)	0.85 (0.58)	51.44 (1.34)	70.29 (1.87)A	41.00 (0.58)B
IR 80564 B	93	105.27 (1.19)	23.40 (1.14)	1.30 (0.60)	19.65 (0.70)	30.95 (1.27)AB	43.10 (0.35)AB
IR60819-34-2 R	98	107.42 (0.66)	22.90 (0.96)	0.63 (0.61)	25.69 (0.77)	39.32 (1.32)AB	42.32 (0.53)AB
Time			38.975***			230.799***	34.615***
Time x Accession			10.180***			17.40ns	1.729ns
Within contrasts			45.473***C			753.489***L	63.466***L
Accession			2.525ns			3.596*	2.313*
IR 81954 H	28	57.50 (0.45)	18.80 (0.86)	1.97 (0.12)	3.09 (0.21)	5.05 (0.34)	37.64 (0.48)
IR 70369 A	28	52.80 (0.95)	12.60 (0.86)	0.02 (0.12)	1.69 (0.21)	2.71 (0.34)	39.28 (0.48)
IR 70369 B	28	55.00 (0.61)	13.20 (0.70)	0.27 (0.08)	2.08 (0.15)	3.35 (0.23)	38.00 (0.32)
IR72889-46-3-2-1 R	28	47.60 (1.54)	12.00 (0.88)	1.76 (0.13)	1.08 (0.25)	1.85 (0.38)	35.44 (0.54)
IR 81954 H	42	89.20 (0.76)	36.00 (0.68)	0.45 (0.08)	9.67 (0.10)	18.12 (0.18)	38.80 (0.14)
IR 70369 A	42	75.60 (1.45)	23.60 (1.48)	1.13 (0.34)	6.54 (0.36)	11.67 (0.69)	38.34 (0.43)
IR 70369 B	42	74.10 (0.61)	23.60 (1.38)	0.85 (0.32)	7.55 (0.45)	13.40 (0.74)	38.00 (0.35)
IR72889-46-3-2-1 R	42	71.10 (0.83)	46.60 (1.10)	0.94 (0.30)	6.33 (0.37)	12.26 (0.60)	38.28 (0.25)
IR 81954 H	56	98.11 (1.09)	28.20 (2.18)	1.57 (0.41)	22.71 (0.51)	39.28 (0.91)	33.62 (0.27)
IR 70369 A	56	92.23 (1.87)	15.80 (1.87)	1.01 (0.94)	14.61 (1.07)	24.62 (2.01)	35.60 (0.66)
IR 70369 B	56	91.69 (0.62)	22.80 (0.74)	0.20 (0.48)	15.77 (0.67)	25.96 (1.11)	38.28 (0.76)
IR72889-46-3-2-1 R	56	94.22 (0.45)	34.00 (1.28)	0.67 (0.77)	15.54 (1.04)	29.21 (1.80)	35.18 (0.15)
IR 81954 H	70	122.50 (0.52)	40.80 (2.70)	0.76 (0.70)	44.71 (0.82)	68.47 (1.52)	39.62 (0.61)
IR 70369 A	70	107.25 (1.76)	32.40 (1.67)	1.25 (0.83)	38.80 (1.40)	54.05 (2.22)	41.88 (0.71)
IR 70369 B	70	111.50 (1.90)	29.40 (1.59)	1.28 (1.15)	37.55 (2.15)	51.82 (3.18)	40.92 (0.24)
IR72889-46-3-2-1 R	70	108.40 (1.74)	52.20 (1.45)	1.51 (0.43)	40.54 (0.70)	69.05 (1.01)	35.72 (0.36)
IR 81954 H	84	131.28 (1.19)	17.00 (0.47)	1.90 (1.73)	23.25 (2.07)	37.15 (3.72)	42.68 (0.62)
IR 70369 A	84	105.00 (2.73)	25.00 (0.75)	2.49 (0.38)	28.39 (1.04)	39.88 (1.38)	42.20 (0.48)
IR 70369 B	84	107.10 (3.33)	15.40 (0.86)	3.08 (0.66)	20.54 (1.74)	30.62 (2.38)	43.75 (0.25)
IR72889-46-3-2-1 R	84	121.82 (2.17)	20.80 (1.11)	2.51 (0.42)	24.79 (1.28)	41.30 (1.68)	38.05 (0.77)

IR 81954 H	98	128.70 (3.02)	19.00 (0.76)AB	3.76 (0.68)	27.53 (0.85)	42.28 (1.43)	38.50 (0.77)AB
IR 70369 A	98	109.62 (1.53)	44.30 (4.19)A	1.86 (0.22)	46.54 (0.89)	62.40 (0.97)	42.16 (0.30)B
IR 70369 B	91	110.27 (1.64)	22.20 (0.99)A	1.10 (1.27)	30.25 (3.59)	45.35 (4.76)	42.40 (0.45)B
IR72889-46-3-2-1 R	112	127.28 (0.52)	33.00 (2.53)B	0.60 (2.27)	45.32 (3.93)	69.91 (6.15)	37.62 (0.56)A
Time			23.581***			113.531***	15.977*** [5,65]
Time x Accession			4.650***			72.950ns	2.168* [15,65]
Within contrasts			48.040***C			376.432***L	33.065***L [1,13]
Accession			6.477***			2.072ns	8.139*** [3,13]
IR 80637 H	28	54.70 (1.60)	14.60 (1.43)	1.47 (0.18)	1.98 (0.24)	3.44 (0.42)	38.32 (0.63)
IR 73328 A	28	44.60 (1.04)	10.60 (0.66)	1.66 (0.05)	0.95 (0.07)	1.61 (0.12)	37.72 (0.35)
IR 73328 B	28	49.70 (0.66)	13.00 (0.81)	0.90 (0.06)	1.40 (0.11)	2.30 (0.16)	41.22 (0.40)
IR73013-95-1-3-2 R	28	44.70 (0.34)	11.00 (0.75)	0.64 (0.02)	0.98 (0.06)	1.61 (0.08)	38.06 (0.19)
IR 80637 H	42	85.90 (1.78)	34.60 (1.82)	1.67 (0.60)	8.17 (0.73)	15.84 (1.32)	38.86 (0.38)
IR 73328 A	42	70.90 (0.95)	30.80 (0.86)	0.70 (0.31)	5.18 (0.41)	9.89 (0.72)	39.12 (0.49)
IR 73328 B	42	74.00 (2.06)	33.00 (1.92)	2.21 (0.33)	6.48 (0.49)	11.69 (0.81)	39.64 (0.25)
IR73013-95-1-3-2 R	42	66.70 (1.65)	32.00 (1.66)	1.05 (0.43)	4.77 (0.33)	9.82 (0.75)	38.36 (0.57)
IR 80637 H	56	98.14 (1.37)	22.60 (1.44)	1.11 (0.49)	13.92 (0.74)	25.03 (1.15)	35.50 (0.47)
IR 73328 A	56	91.36 (0.46)	32.60 (1.27)	0.03 (0.42)	15.33 (0.43)	26.37 (0.69)	38.02 (0.42)
IR 73328 B	56	90.55 (0.92)	26.60 (1.68)	0.55 (0.80)	15.78 (0.92)	26.33 (1.71)	38.18 (0.49)
IR73013-95-1-3-2 R	56	82.86 (0.55)	31.60 (0.86)	0.89 (0.18)	12.46 (0.58)	23.35 (0.73)	34.20 (0.38)
IR 80637 H	70	114.20 (0.65)	38.00 (0.97)	0.87 (1.34)	41.58 (1.71)	63.45 (3.05)	40.40 (0.33)
IR 73328 A	70	111.20 (0.48)	42.80 (2.50)	0.67 (0.99)	42.53 (2.37)	58.20 (3.28)	43.06 (0.57)
IR 73328 B	70	114.75 (1.07)	33.40 (0.77)	1.76 (0.15)	33.22 (0.54)	45.98 (0.50)	40.08 (0.10)
IR73013-95-1-3-2 R	70	93.60 (1.11)	49.00 (1.14)	1.48 (0.96)	31.87 (1.03)	56.35 (1.99)	39.10 (0.52)
IR 80637 H	84	121.96 (1.53)	25.40 (0.32)	1.79 (0.69)	24.32 (1.14)	39.11 (1.62)	41.70 (0.47)
IR 73328 A	84	111.76 (1.23)	22.20 (0.48)	1.20 (0.66)	24.70 (2.04)	34.90 (2.60)	42.80 (0.31)
IR 73328 B	84	114.40 (1.92)	22.20 (0.62)	1.83 (0.46)	19.44 (0.62)	29.27 (1.03)	40.44 (0.63)
IR73013-95-1-3-2 R	84	112.64 (1.43)	22.00 (1.44)	1.56 (0.44)	25.52 (0.72)	41.08 (1.13)	41.26 (0.43)
IR 80637 H	98	123.95 (1.92)	22.80 (0.94)	1.62 (1.38)	32.62 (2.32)	51.23 (3.64)	37.46 (0.13)AB
IR 73328 A	98	117.56 (0.76)	48.50 (2.90)	0.21 (0.68)	36.05 (1.36)	49.26 (1.95)	39.42 (0.47)B
IR 73328 B	93	113.14 (0.91)	22.00 (0.80)	0.82 (0.28)	24.29 (1.03)	34.11 (1.27)	38.48 (0.55)B
IR73013-95-1-3-2 R	112	117.29 (0.51)	30.80 (0.66)	0.20 (0.75)	41.26 (2.77)	62.46 (3.52)	39.82 (0.55)A
Time			43.695***			185.443***	11.739*** [5,75]
Time x Accession			4.920***			3.270***	1.305ns [15,75]

Within contrasts			97.294***Q			496.102***L	17.096***C [1,15]
Accession			1.175ns			2.754ns	5.389** [3,15]
IR 82385 H	28	52.60 (0.98)	18.20 (1.23)	0.38 (0.16)	2.05 (0.18)	3.43 (0.33)	38.68 (0.36)
IR 79125 A	28	41.40 (1.06)	8.80 (1.16)	1.58 (0.10)	0.79 (0.13)	1.37 (0.23)	38.48 (0.49)
IR 79125 B	28	48.60 (1.13)	24.60 (2.80)	1.47 (0.14)	2.41 (0.24)	3.87 (0.38)	38.46 (0.37)
IR73717-46-1-3-3 R	28	46.80 (0.82)	12.40 (1.20)	0.89 (0.09)	1.33 (0.15)	2.23 (0.24)	37.92 (0.38)
IR 82385 H	42	71.00 (1.47)	38.60 (2.29)	1.31 (0.57)	6.66 (0.71)	12.97 (1.29)	38.56 (0.38)
IR 79125 A	42	61.90 (1.17)	44.00 (1.37)	1.06 (0.20)	5.63 (0.33)	10.69 (0.51)	38.16 (0.52)
IR 79125 B	42	61.80 (0.67)	45.20 (1.13)	0.39 (0.18)	5.48 (0.26)	10.87 (0.43)	38.40 (0.69)
IR73717-46-1-3-3 R	42	67.80 (1.35)	34.80 (1.96)	1.62 (0.36)	5.69 (0.44)	10.31 (0.79)	38.26 (0.39)
IR 82385 H	56	87.52 (0.35)	29.80 (1.06)	0.69 (0.38)	14.56 (0.48)	26.26 (0.78)	34.52 (0.23)
IR 79125 A	56	71.90 (2.04)	28.00 (1.42)	2.77 (0.27)	10.15 (0.16)	18.92 (0.39)	36.58 (0.40)
IR 79125 B	56	79.44 (1.80)	29.60 (1.46)	1.14 (0.33)	16.09 (0.37)	28.22 (0.59)	36.24 (0.48)
IR73717-46-1-3-3 R	56	83.70 (1.61)	25.20 (2.10)	1.20 (0.48)	14.23 (0.91)	24.43 (1.32)	36.84 (0.72)
IR 82385 H	70	102.20 (2.16)	42.00 (1.49)	2.76 (1.37)	35.53 (1.94)	57.29 (3.21)	41.26 (0.45)
IR 79125 A	70	99.75 (1.92)	39.20 (0.48)	1.09 (0.44)	31.95 (0.47)	50.04 (0.47)	40.50 (0.73)
IR 79125 B	70	97.20 (0.73)	37.60 (1.94)	0.30 (0.80)	29.73 (1.64)	45.03 (2.42)	42.60 (0.44)
IR73717-46-1-3-3 R	70	106.00 (1.04)	35.20 (1.30)	1.69 (1.14)	32.47 (1.48)	49.16 (2.27)	40.38 (0.13)
IR 82385 H	84	115.98 (1.58)	19.60 (1.15)	1.28 (1.18)	27.93 (1.48)	46.21 (2.52)	43.06 (0.55)
IR 79125 A	84	104.72 (0.85)	20.00 (1.56)	0.59 (0.45)	24.59 (0.94)	37.18 (1.24)	43.60 (1.09)
IR 79125 B	84	110.70 (1.85)	21.20 (1.16)	1.27 (0.48)	18.92 (0.69)	31.18 (0.97)	43.48 (0.36)
IR73717-46-1-3-3 R	84	104.96 (1.46)	23.00 (0.51)	1.49 (0.39)	18.44 (0.59)	28.92 (0.93)	41.14 (0.59)
IR 82385 H	112	120.86 (0.87)	30.90 (1.79)	0.73 (2.01)	63.55 (3.42)	94.28 (5.39)B	39.00 (0.62)
IR 79125 A	98	108.06 (0.57)	47.20 (3.38)	0.43 (1.56)	45.54 (3.18)	66.96 (4.65)A	39.92 (0.34)
IR 79125 B	93	111.82 (0.69)	24.60 (1.01)	0.05 (0.81)	24.13 (0.55)	38.18 (1.25)A	39.20 (0.33)
IR73717-46-1-3-3 R	93	104.76 (1.28)	27.90 (1.04)	1.76 (0.55)	22.50 (0.95)	33.26 (1.45)A	40.04 (0.39)
Time			35.649***			235.446***	17.262*** [5,75]
Time x Accession)			3.021***			6.087***	0.572ns [15,75]
Within contrasts			61.967***L			642.252***L	26.901***C [1,15]
Accession			0.993ns			7.567***	0.541ns [3,15]
IR 82363 H	28	51.00 (0.72)	23.00 (1.30)	0.62 (0.14)	2.70 (0.23)	4.31 (0.37)	37.46 (0.39)
IR 68897 A	28	45.80 (1.12)	20.00 (2.02)	1.30 (0.13)	2.28 (0.28)	3.58 (0.40)	37.58 (0.37)
IR 68897 B	28	45.67 (2.08)	15.33 (3.66)	2.07 (0.24)	1.77 (0.48)	2.84 (0.71)	37.07 (1.05)
SRT 3 R	28	42.30 (0.97)	11.80 (0.65)	0.65 (0.06)	1.02 (0.10)	1.67 (0.16)	38.12 (0.34)

IR 82363 H	42	74.80 (2.10)	43.40 (2.59)	2.46 (0.31)	9.51 (0.87)	16.97 (1.15)	38.30 (0.26)
IR 68897 A	42	61.50 (0.24)	39.60 (1.81)	0.78 (0.26)	6.43 (0.31)	12.21 (0.56)	38.00 (0.28)
IR 68897 B	42	62.00 (1.00)	54.33 (3.53)	1.31 (0.45)	8.09 (0.70)	15.40 (1.13)	38.30 (0.67)
SRT 3 R	42	64.10 (0.59)	38.40 (1.17)	0.48 (0.20)	5.68 (0.32)	11.16 (0.50)	40.50 (0.30)
IR 82363 H	56	89.13 (1.42)	33.20 (1.71)	1.57 (0.84)	20.43 (0.52)	35.01 (1.31)	36.20 (0.29)
IR 68897 A	56	77.58 (1.78)	27.60 (1.79)	1.35 (0.69)	13.98 (1.05)	24.34 (1.69)	37.14 (0.15)
IR 68897 B	56	79.15 (2.13)	53.33 (0.77)	2.92 (1.57)	21.84 (3.49)	36.76 (5.00)	32.40 (0.49)
SRT 3 R	56	77.18 (0.40)	23.20 (1.23)	0.94 (0.41)	11.04 (0.40)	20.98 (0.79)	38.28 (0.43)
IR 82363 H	70	111.20 (1.90)	47.80 (1.12)	1.93 (0.77)	44.87 (1.61)	65.80 (2.23)	38.02 (0.15)
IR 68897 A	70	94.13 (1.82)	39.60 (1.55)	1.67 (0.82)	30.81 (2.14)	44.48 (2.94)	40.06 (0.30)
IR 68897 B	70	97.00 (0.88)	34.00 (3.18)	0.25 (0.66)	17.44 (0.82)	27.69 (0.37)	39.50 (0.39)
SRT 3 R	70	92.20 (0.65)	36.60 (1.07)	0.88 (0.99)	28.56 (1.96)	46.44 (2.93)	41.64 (0.49)
IR 82363 H	84	111.50 (1.36)	17.80 (0.92)	1.87 (0.45)	17.25 (0.67)	28.12 (1.07)	37.02 (0.40)
IR 68897 A	84	92.16 (1.95)	37.40 (3.47)	1.50 (0.56)	25.71 (0.95)	39.21 (1.35)	38.86 (0.37)
IR 68897 B	84	95.83 (1.73)	29.33 (3.86)	1.36 (2.02)	17.22 (3.45)	26.57 (5.34)	40.73 (0.13)
SRT 3 R	84	100.56 (1.43)	24.40 (1.90)	1.37 (0.92)	26.07 (1.75)	42.44 (2.67)	42.38 (0.36)
IR 82363 H	93	114.68 (1.89)	29.90 (1.32)AB	1.78 (0.77)	28.39 (1.16)	44.17 (1.70)B	37.80 (0.43)
IR 68897 A	112	92.03 (0.27)	65.60 (3.17)B	0.53 (0.50)	43.19 (0.78)	59.72 (1.08)A	34.96 (0.34)
IR 68897 B	93	102.97 (2.61)	29.00 (0.67)B	2.65 (0.28)	18.92 (0.58)	28.58 (0.85)A	39.07 (1.43)
SRT 3 R	112	108.25 (0.69)	26.20 (0.67)A	0.51 (0.79)	34.80 (0.83)	53.30 (1.35)A	40.54 (0.38)
Time			18.462*** [5,70]			133.577*** [5,70]	5.075*** [5,70]
Time x Accession			5.616*** [15,70]			2.973*** [15,70]	1.122ns [15,70]
Within contrasts			43.425***Q [1,14]			794.312***L [1,14]	11.149***L [1,14]
Accession			11.537*** [3,14]			6.838*** [3,14]	3.024ns [3,14]

1: Numbers are means (SEM) (N = 5); Degrees of freedom for Time = 5,80 and Time x Accession = 15,80; for within subject contrasts = 1,16; and for Accession = 3,16, unless indicated by square brackets; ns = P > 0.05, * = P < 0.05, ** = P < 0.01, *** = P < 0.001; C = cubic, L = linear and Q = quadratic relation associated with within subject contrasts; uppercase numbers indicate homogenous line groups incorporating repeated measures (Tukey P > 0.05); 2: Note that some of the results for shoot biomass and tiller numbers have been summarized by plant-type and presented in a related paper by Horgan et al (2016); the data and information by accession and other data from this table have not been previously published.

Table S10: Hybrid growth parameters in field plots at IRRI. Results are summarized from Table S9 and visualized in Figure 2A-C.

Accession	Sampling Period (DAT)	Total Number of Tiller[1,2]	Total Plant Dry Weight (g) [1,2,3]	SPAD-value[1,3]
IR82396H	28	16.00 (0.73)	3.25 (0.22)abc	39.04 (0.47)ab
	42	46.00 (2.85)	14.02 (0.80)	38.94 (0.42)
	56	28.40 (1.54)	30.89 (1.58)	36.44 (0.62)
	70	44.40 (1.74)	52.84 (1.00)	42.12 (0.31)
	84	26.20 (1.37)	36.78 (0.91)	42.15 (0.55)
	98	31.80 (1.80)	42.85 (3.90)	38.22 (0.56)
IR 82391 H	28	15.60 (1.35)	2.80 (0.31)ab	38.24 (0.38)ab
	42	44.60 (1.80)	14.20 (0.68)	39.62 (0.48)
	56	31.00 (1.76)	27.29 (1.05)	36.92 (0.85)
	70	41.60 (1.53)	50.63 (2.21)	42.88 (0.27)
	84	24.80 (0.91)	30.60 (1.03)	40.92 (0.36)
	93	25.20 (1.80)	30.36 (1.98)	39.98 (0.59)
IR 84714 H	28	17.80 (1.19)	3.00 (0.21)a	40.56 (0.28)b
	42	42.00 (2.55)	14.06 (0.56)	41.78 (0.51)
	56	28.20 (2.13)	26.97 (1.20)	38.12 (0.51)
	70	33.60 (1.45)	42.17 (1.41)	44.84 (0.26)
	84	20.00 (1.03)	25.62 (0.90)	43.80 (0.38)
	93	26.60 (0.76)	32.31 (0.75)	42.20 (0.30)
IR 85471 H	28	17.60 (0.98)	3.70 (0.30)abc	38.32 (0.63)ab
	42	34.60 (1.90)	13.79 (0.85)	38.78 (0.37)
	56	28.20 (1.05)	30.80 (0.67)	37.42 (0.39)
	70	41.40 (1.20)	57.37 (2.18)	41.36 (0.30)
	84	20.60 (0.82)	28.60 (0.45)	42.04 (0.51)
	93	24.20 (1.02)	31.43 (1.16)	40.00 (0.31)
IR 81954 H	28	18.80 (0.86)	5.05 (0.34)abc	37.64 (0.48)a
	42	36.00 (0.68)	18.12 (0.18)	38.80 (0.14)
	56	28.20 (2.18)	39.28 (0.91)	33.62 (0.27)
	70	40.80 (2.70)	68.47 (1.52)	39.62 (0.61)
	84	17.00 (0.47)	37.15 (3.72)	42.68 (0.62)
	98	19.00 (0.76)AB	42.28 (1.43)	38.50 (0.77)
IR 80637 H	28	14.60 (1.43)	3.44 (0.42)abc	38.32 (0.63)ab

	42	34.60 (1.82)	15.84 (1.32)	38.86 (0.38)
	56	22.60 (1.44)	25.03 (1.15)	35.50 (0.47)
	70	38.00 (0.97)	63.45 (3.05)	40.40 (0.33)
	84	25.40 (0.32)	39.11 (1.62)	41.70 (0.47)
	98	22.80 (0.94)	51.23 (3.64)	37.46 (0.13)
IR 82385 H	28	18.20 (1.23)	3.43 (0.33)c	38.68 (0.36)ab
	42	38.60 (2.29)	12.97 (1.29)	38.56 (0.38)
	56	29.80 (1.06)	26.26 (0.78)	34.52 (0.23)
	70	42.00 (1.49)	57.29 (3.21)	41.26 (0.45)
	84	19.60 (1.15)	46.21 (2.52)	43.06 (0.55)
	112	30.90 (1.79)	94.28 (5.39)	39.00 (0.62)
IR 82363 H	28	23.00 (1.30)	4.31 (0.37)bc	37.46 (0.39)a
	42	43.40 (2.59)	16.97 (1.15)	38.30 (0.26)
	56	33.20 (1.71)	35.01 (1.31)	36.20 (0.29)
	70	47.80 (1.12)	65.80 (2.23)	38.02 (0.15)
	84	17.80 (0.92)	28.12 (1.07)	37.02 (0.40)
	93	29.90 (1.32)	44.17 (1.70)	37.80 (0.43)
F-Time		76.569***	380.614***	6.614***
F-Time x Accession		52.621ns	3.678ns	0.621ns
F-Accession		1.683ns	3.699***	3.030*

1: Numbers are means (SEM) (N = 5); Degrees of freedom for Time = 5,160, Time x Accession = 35,160; and for Accession = 7,32; ns = P > 0.05, * = P < 0.05, *** = P < 0.001; lowercase letters indicate homogenous hybrid groups incorporating repeated measures (Tukey P > 0.05); 2: Note that some of the results for shoot biomass and tiller numbers have been summarized by plant-type and presented in a related paper by Horgan et al (2016); the data and information by accession and other data from this table have not been previously published. 3: Blue = heterosis, green = heterobeltiosis for biomass

Table S11: Plant reproductive parameters in field plots at IRRI. Results are summarized in Table S12 and Figure 2.

Accession	Proportion of Tillers with Panicles	Rachis Dry Weight (g) [1]	Dry Weight of Reproductive Structures (g) [1]	Number of Panicles[1]	Total Number of Filled Grain per Plant[1]	Total Dry Weight of Filled Grain per Plant (g) [1]	1000-Grain Fresh Weight (g) [1]	Total Number of Unfilled Grains[1]	Proportion of Grain that was Unfilled[1]
IR82396H	29.60 (1.56)A	3.18 (0.32)AB	50.13 (4.87)	28.40 (2.69)A	2079.40 (190.76)B	42.91 (4.15)B	21.71 (0.21)AB	914.40 (112.64)A	30.54 (3.71)A
IR80156A	53.90 (0.91)B	4.49 (0.13)B	20.86 (0.73)	61.80 (0.97)B	146.00 (28.70)A	2.69 (0.57)A	18.92 (0.31)A	5249.20 (126.17)B	97.29 (8.15)C
IR80156B	27.40 (1.72)A	2.34 (0.15)A	35.10 (2.33)	25.20 (1.60)A	1347.40 (100.60)AB	26.51 (1.76)AB	21.05 (0.22)AB	1225.40 (128.30)A	47.63 (5.61)B
IR46R	28.50 (1.14)A	3.16 (0.23)AB	50.86 (4.74)	32.80 (1.63)A	1891.20 (173.16)B	43.47 (4.20)B	24.12 (0.70)B	943.40 (50.55)A	33.28 (2.26)AB
F-Accession	17.311***	3.258*	3.112ns	16.788***	7.841***	7.674***	5.377**	75.173***	89.284***
IR 82391 H	24.00 (1.62)	2.19 (0.16)A	34.30 (2.36)AB	21.80 (1.57)	1306.80 (102.61)AB	28.50 (2.16)B	23.12 (0.28)B	840.40 (60.02)A	39.14 (3.73)A
IR 79156 A	27.60 (1.48)	2.87 (0.15)AB	16.94 (1.22)A	28.40 (0.33)	474.60 (47.11)A	7.87 (0.78)A	17.51 (0.12)A	2539.20 (.317)B	84.25 (6.22)B
IR 79156 B	32.10 (2.16)	4.01 (0.20)B	47.76 (2.39)B	33.60 (1.74)	2076.40 (130.54)B	38.42 (1.88)B	20.20 (0.66)AB	1393.20 (80.53)A	40.15 (5.59)A
IR60819-34-2	21.80 (0.38)	2.78 (0.10)AB	40.49 (1.47)B	24.20 (1.56)	1567.80 (75.39)B	33.45 (1.47)B	22.66 (0.26)B	949.60 (75.33)A	37.72 (4.34)A
F-Accession	1.684ns	4.740*	9.255***	2.667ns	10.097***	13.140***	8.841***	11.963***	21.230***
IR 84714 H	24.50 (0.69)A	2.14 (0.18)Aa	39.70 (1.22)B	18.50 (1.70)A	1216.35 (103.52)B	29.65 (2.55)B	25.88 (0.29)B	637.50 (61.47)Aa	34.39 (3.73)A
IR 80559 A	53.70 (2.36)B	4.34 (0.18)B	19.97 (1.07)A	56.80 (2.78)B	222.20 (22.69)A	4.52 (0.44)A	21.79 (0.22)A	4341.20 (267.31)B	95.13 (9.22)C
IR 80559 B	29.80 (1.18)A	2.34 (0.09)Aab	38.91 (2.07)B	28.40 (1.15)A	1495.80 (86.88)B	30.65 (2.01)B	21.43 (0.23)A	1389.80 (55.15)Ab	48.16 (3.88)B
IR60819-34-2 R	21.70 (0.67)A	3.29 (0.11)Abb	45.97 (1.04)B	25.20 (0.59)A	1851.80 (51.54)B	37.91 (0.75)B	21.73 (0.27)A	1221.60 (122.18)Aab	39.75 (7.03)A
F-Accession	21.599***	9.550***	12.628***	18.522***	18.270***	15.035***	14.004***	23.673***	150.792***
IR 85471 H	23.20 (1.05)A	2.44 (0.10)A	44.29 (2.44)	22.80 (0.86)A	1618.80 (87.44)B	38.53 (2.32)B	25.08 (0.37)	752.20 (63.82)A	31.73 (4.22)A
IR 80564 A	53.70 (3.19)B	6.39 (0.31)B	31.22 (2.83)	62.60 (2.99)B	283.30 (66.99)A	6.28 (1.68)A	20.88 (0.69)	4772.39 (574.31)B	94.40 (8.96)B
IR 80564 B	22.60 (1.04)A	2.23 (0.13)A	39.96 (1.23)	22.00 (1.36)A	1315.05 (93.66)B	29.24 (1.96)B	21.24 (1.07)	793.90 (67.56)A	37.64 (4.19)AB
IR60819-34-2 R	21.70 (0.67)A	3.29 (0.11)A	45.97 (1.04)	25.20 (0.59)A	1851.80 (51.54)B	37.91 (0.75)B	21.73 (0.27)	1221.60 (122.18)A	39.75 (7.03)AB
F-Accession	15.227***	22.275***	2.102ns	26.108***	16.250***	14.477***	1.623ns	8.490***	4.555*
IR 81954 H	17.50 (0.76)A	3.09 (0.14)	49.34 (1.56)AB	18.20 (0.52)	1701.60 (66.31)B	42.15 (1.48)B	26.24 (0.19)B	790.80 (59.50)A	31.76 (4.73)A
IR 70369 A	35.30 (0.76)B	4.04 (0.14)	22.60 (1.56)A	30.20 (0.52)	378.00 (66.31)A	8.02 (1.48)A	22.06 (0.19)A	2922.00 (59.50)B	88.55 (4.73)B
IR 70369 B	21.00 (2.21)A	2.21 (0.42)	44.32 (2.27)AB	18.00 (2.58)	1569.60 (47.50)AB	39.57 (1.03)B	26.47 (0.22)B	409.60 (393.67)A	20.70 (8.93)A
IR72889-46-3-2-1 R	27.60 (1.79)AB	4.48 (0.44)	57.74 (6.29)B	35.20 (3.08)	2268.40 (255.65)B	48.35 (5.58)B	22.34 (0.36)A	1109.80 (89.66)AB	32.85 (2.60)A
F-Accession	4.953*	2.052ns	3.244*	2.893ns	6.132**	6.239***	11.509***	5.929**	62.372***
IR 80637 H	21.50 (0.88)Aa	3.94 (0.35)ab	63.65 (5.70)B	23.60 (1.60)A	2506.20 (238.19)C	54.74 (4.86)B	23.29 (0.30)B	1082.80 (121.08)A	30.17 (3.37)Aa
IR 73328 A	45.50 (2.87)B	3.77 (0.25)	21.34 (1.10)A	49.00 (3.48)B	287.40 (33.89)A	5.50 (0.61)A	20.57 (0.23)A	3362.00 (407.50)B	92.12 (9.23)B
IR 73328 B	20.40 (0.97)Aa	1.96 (0.13)a	32.69 (2.23)AB	16.60 (1.26)A	1179.00 (82.03)AB	27.51 (1.92)AB	24.63 (0.16)B	647.60 (39.36)A	35.45 (3.24)Ab
IR73013-95-1-3-2 R	29.80 (0.57)Ab	4.19 (0.26)b	52.99 (3.86)AB	29.00 (1.12)A	1781.80 (117.45)BC	41.52 (3.02)B	24.41 (0.30)B	1409.20 (93.32)AB	44.16 (4.43)Ac

F-Accession	10.483***	3.038ns	5.477**	8.872***	9.014***	9.627***	10.730***	6.023**	22.896***
IR 82385 H	28.70 (1.55)	3.43 (0.27)AB	40.95 (1.58)**B**	21.10 (0.95)A	1451.00 (89.16)B	35.04 (1.34)B	25.56 (0.10)	871.40 (79.28)Ab	37.51 (5.69)A
IR 79125 A	38.20 (3.13)	5.21 (0.50)B	20.20 (1.50)A	38.20 (3.32)B	93.60 (13.63)A	2.05 (0.32)A	22.54 (0.53)	4267.80 (388.31)B	97.85 (9.66)B
IR 79125 B	23.00 (0.84)	2.59 (0.24)A	42.28 (1.05)B	21.05 (1.78)A	1254.05 (124.83)B	29.13 (2.76)B	21.21 (1.86)	759.85 (58.96)Ab	37.73 (3.21)A
IR73717-46-1-3-3 R	24.00 (0.55)	1.86 (0.17)A	34.73 (1.71)AB	18.85 (1.61)A	956.95 (65.16)B	25.79 (2.17)B	24.37 (2.29)	344.95 (32.85)Aa	26.50 (3.35)A
F-Accession	2.920ns	8.849***	31.481***	5.490**	32.259***	32.662***	0.750ns	13.751***	79.213***
IR 82363 H	27.80 (1.14)A	2.86 (0.13)AB	49.88 (2.62)B	23.40 (0.59)	1723.60 (86.61)B	43.27 (2.22)B	26.48 (0.09)Cb	641.80 (53.88)A	27.13 (3.84)A
IR 68897 A	54.30 (2.71)B	4.18 (0.14)C	17.53 (0.54)A	37.80 (4.48)	127.60 (7.26)A	2.08 (0.16)A	17.23 (0.75)A	4070.20 (146.72)B	96.96 (9.53)B
IR 68897 B	26.67 (0.84)A	2.16 (0.07)A	43.52 (1.81)B	25.67 (1.39)	1791.33 (75.74)B	37.61 (1.27)B	22.22 (0.28)Ba	752.00 (59.07)A	29.57 (4.38)A
SRT 3 R	24.40 (0.33)A	3.52 (0.11)BC	50.74 (2.52)B	32.00 (1.33)	2036.20 (122.70)B	42.64 (2.88)B	21.85 (0.35)Ba	845.80 (70.99)A	29.35 (3.67)A
F-Accession	15.134***	12.021***	8.503***	1.240ns	21.896***	19.738***	14.159***	62.044***	80.850***

1: Numbers are means (SEM) (N = 5); Degrees of freedom for Accession = 3,20; ns = P > 0.05, * = P < 0.05, *** = P < 0.001; uppercase letters indicate homogenous accession groups (Tukey P > 0.05)

Table S12: Hybrid reproductive parameters in field plots at IRRI. Results are summarized from Table S11 and visualized in Figure 2D-F.

Accession	Proportion of Tillers with Panicles	Rachis Dry Weight (g) [1,2]	Dry Weight of Reproductive Structures (g) [1]	Number of Panicles[1]	Total Number of Filled Grain per Plant[1,2]	Total Dry Weight of Filled Grain per Plant (g) [1]	1000-Grain Fresh Weight (g) [1,2]	Total Number of Unfilled Grains[1,2]	Proportion of Grain that was Unfilled[1,2]
IR82396H	29.60 (1.56)	3.18 (0.32)	50.13 (4.87)AB	28.40 (2.69)	2079.40 (190.76)B	42.91 (4.15)B	21.71 (0.21)A	914.40 (112.64)	30.54 (3.71)
IR 82391 H	24.00 (1.62)	2.19 (0.16)	34.30 (2.36)A	21.80 (1.57)	1306.80 (102.61)A	28.50 (2.16)A	23.12 (0.28AB	840.40 (60.02)	39.14 (3.73)
IR 84714 H	24.50 (0.69)	2.14 (0.18)	39.70 (1.22)A	18.50 (1.70)	1216.35 (103.52)A	29.65 (2.55)A	25.88 (0.29)C	637.50 (61.47)	34.39 (3.73)
IR 85471 H	23.20 (1.05)	2.44 (0.10)	44.29 (2.44)A	22.80 (0.86)	1618.80 (87.44)AB	38.53 (2.32)A	25.08 (0.37)BC	752.20 (63.82)	31.73 (4.22)
IR 81954 H	17.50 (0.76)	3.09 (0.14)	49.34 (1.56)AB	18.20 (0.52)	1701.60 (66.31)AB	42.15 (1.48)B	26.24 (0.19)C	790.80 (59.50)	31.76 (4.73)
IR 80637 H	21.50 (0.88)	3.94 (0.35)	63.65 (5.70)B	23.60 (1.60)	2506.20 (238.19)B	54.74 (4.86)B	23.29 (0.30)AB	1082.80 (121.08)	30.17 (3.37)
IR 82385 H	28.70 (1.55)	3.43 (0.27)	40.95 (1.58)A	21.10 (0.95)	1451.00 (89.16)A	35.04 (1.34)A	25.56 (0.10)BC	871.40 (79.28)	37.51 (5.69)
IR 82363 H	27.80 (1.14)	2.86 (0.13)	49.88 (2.62)AB	23.40 (0.59)	1723.60 (86.61)AB	43.27 (2.22)B	26.48 (0.09)C	641.80 (53.88)	27.13 (3.84)
F-Accession	2.236ns	1.058ns	4.229***	1.707ns	3.657***	4.071***	9.939***	0.963ns	1.096ns

1: Numbers are means (SEM) (N = 5); Degrees of freedom for Accession = 7,34; ns = P > 0.05, *** = P < 0.001; uppercase letters indicate homogenous accession groups (Tukey P > 0.05); 2: Blue indicates heterosis, green indicates heterobeltiosis for fitness parameter

Table S13: Yields of filled and unfilled grain from crop cuts in field plots at IRRI. Results are summarized in Table 1

Accession	Harvest Time (DAT)	Filled Grain (Tonnes $Hectare^{-1}$)[1,2]	Unfilled Grain (Tonnes $Hectare^{-1}$)[1,2]	1000 Grain Fresh Weight (g)[1,2]
IR82396H	98	6.43 (0.12)Cb	0.67 (0.05)	21.12 (0.19)
IR80156A	112	0.68 (0.09)A	1.31 (0.11)	20.72 (0.24)
IR80156B	93	4.62 (0.19)BCab	1.04 (0.16)	20.01 (0.16)
IR46R	112	3.10 (0.49)ABa	1.40 (0.43)	20.17 (0.12)
F-Accession		16.122***	0.679ns	1.536ns
IR 82391 H	93	5.73 (0.30)Cb	0.85 (0.03)	20.90 (0.23)Bb
IR 79156 A	98	1.57 (0.07)A	1.09 (0.77)	19.10 (0.50)AB
IR 79156 B	98	3.33 (0.26)ABa	0.63 (0.22)	17.52 (0.33)Aa
IR60819-34-2	93	4.15 (0.26)BCab	0.65 (0.44)	19.85 (0.20)ABb
F-Accession		10.251***	2.954ns	3.617*
IR 84714 H	93	7.02 (0.10)Dc	0.55 (0.36)	22.67 (0.30)
IR 80559 A	112	0.38 (0.04)A	1.10 (0.47)	20.95 (0.07)
IR 80559 B	93	3.81 (0.08)Ba	0.81 (0.74)	20.27 (0.40)
IR60819-34-2 R	98	4.84 (0.08)Cb	1.60 (0.43)	20.97 (0.20)
F-Accession		252.981***	0.858ns	2.832ns
IR 85471 H	93	6.24 (0.18)Cb	0.70 (0.10)	22.25 (0.49)
IR 80564 A	112	0.20 (0.02)A	1.86 (0.52)	21.66 (0.11)
IR 80564 B	93	5.02 (0.14)Ba	0.65 (0.77)	21.30 (0.15)
IR60819-34-2 R	98	4.84 (0.08)Ba	1.60 (0.43)	20.97 (0.20)
F-Accession		92.806***	0.949ns	0.761ns
IR 81954 H	98	5.68 (0.20)C	0.83 (0.34)	25.25 (0.14)Bb
IR 70369 A	98	0.37 (0.03)A	1.16 (0.76)	22.34 (0.47)A
IR 70369 B	91	3.93 (0.27)B	0.68 (0.56)	22.23 (0.29)Aa
IR72889-46-3-2-1 R	112	4.42 (0.14)B	0.92 (0.11)	22.25 (0.16)Aa
F-Accession		29.735***	2.518ns	5.001*
IR 80637 H	98	5.20 (0.33)B	0.71 (0.34)A	22.47 (0.29)B
IR 73328 A	98	0.63 (0.09)A	1.73 (0.20)B	18.86 (0.46)A
IR 73328 B	91	5.01 (0.06)B	0.62 (0.05)A	20.86 (0.22)AB

IR73013-95-1-3-2 R	112	5.17 (0.26)B	1.02 (0.79)A	21.52 (0.35)AB
F-Accession		21.941***	24.795***	4.045*
IR 82385 H	112	6.38 (0.10)Cb	0.73 (0.43)ABab	22.88 (0.78)
IR 79125 A	98	0.31 (0.04)A	1.46 (0.51)C	21.59 (0.30)
IR 79125 B	93	4.62 (0.22)Ba	1.05 (0.58)BCb	23.41 (0.17)
IR73717-46-1-3-3 R	93	5.30 (0.22)BCab	0.41 (0.68)Aa	25.07 (0.55)
F-Accession		52.307***	15.739***	1.618ns
IR 82363 H	93	6.30 (0.22)B	0.70 (0.54)A	23.50 (0.25)Bb
IR 68897 A	112	0.27 (0.02)A	1.51 (0.42)B	21.16 (0.15)AB
IR 68897 B	93	4.28 (0.99)B	1.01 (0.41)AB	17.54 (1.68)Aa
SRT 3 R	112	4.82 (0.12)B	0.76 (0.32)A	19.57 (0.48)Ba
F-Accession		19.665*** [7,14]	4.994* [7,14]	4.322* [7,14]

1: Numbers are means (SEM) (N = 5); Degrees of freedom for Accession = 7,16, except where indicated by square brackets; * = $P < 0.05$, *** = $P < 0.001$; uppercase letters indicate homogenous accession groups based on comparisons of all four plant types, lowercase letters indicate homogenous groups excluding the A-lines from analyses (Tukey $P > 0.05$); 2: Note that these results have been summarized by plant-type and presented in a related paper by Horgan et al (2016); the data and information by accession have not been previously published.

Table S14: Results from BlowVac sampling of rice field plots at IRRI. Results are summarized in Table 2

Accession	Sampling Time (DAT)	BPH (number per sample)	WBPH (Number per Sample)	Other Hemiptera (Number per Sample)	Virus Vectors (Number per Sample)
IR82396H	21	0.20 (0.09)	2.80 (0.63)	0.00 (0.00)	3.40 (0.91)
IR80156A	21	0.20 (0.09)	2.60 (0.63)	0.00 (0.00)	2.40 (0.60)
IR80156B	21	0.00 (0.00)	2.60 (0.56)	0.20 (0.09)	3.40 (1.00)
IR46R	21	0.40 (0.11)	3.20 (0.94)	0.80 (0.36)	5.20 (1.35)
IR82396H	56	15.20 (2.38)	16.20 (2.99)	1.00 (0.45)	43.00 (8.57)
IR80156A	56	19.80 (2.68)	23.20 (2.92)	0.80 (1.29)	28.80 (4.12)
IR80156B	56	24.60 (4.32)	32.00 (3.47)	2.20 (0.76)	26.00 (4.72)
IR46R	56	21.40 (5.26)	33.00 (11.46)	0.60 (0.27)	30.80 (9.52)
IR82396H	84	0.80 (0.26)	3.20 (0.49)	0.40 (0.18)	9.60 (1.08)A
IR80156A	84	1.20 (0.22)	3.00 (0.48)	0.40 (0.18)	11.80 (2.28)A
IR80156B	84	0.40 (0.11)	2.40 (0.47)	1.80 (0.74)	9.80 (1.77)B
IR46R	84	1.60 (0.23)	4.80 (0.61)	0.60 (0.20)	11.80 (2.35)AB
F-time		20.558***	10.730***	11.763***	3.197ns
F time x Accession		0.214ns	0.307ns	0.277ns	0.938ns
F-accession		0.204ns	0.374ns	0.227ns	4.352*
IR 82391 H	21	0.40 (0.18)	3.60 (0.87)	0.00 (0.00)	2.80 (0.79)
IR 79156 A	21	0.20 (0.09)	7.80 (1.45)	0.40 (0.18)	5.00 (1.16)
IR 79156 B	21	0.20 (0.09)	0.80 (0.22)	0.00 (0.00)	0.60 (0.27)
IR60819-34-2	21	0.40 (0.11)	4.20 (0.79)	0.00 (0.00)	4.20 (1.05)
IR 82391 H	56	11.60 (2.19)	27.40 (4.53)	1.20 (0.38)	26.40 (4.57)
IR 79156 A	56	30.20 (3.86)	33.20 (2.28)	0.80 (0.29)	37.00 (6.21)
IR 79156 B	56	36.80 (7.42)	43.40 (5.97)	0.20 (0.09)	46.00 (7.87)
IR60819-34-2	56	33.40 (5.74)	30.40 (6.47)	0.40 (0.18)	23.20 (4.60)
IR 82391 H	84	2.40 (0.30)	4.00 (0.87)	0.60 (0.27)	13.80 (1.87)
IR 79156 A	84	1.60 (0.46)	4.00 (0.60)	0.60 (0.27)	16.40 (1.93)
IR 79156 B	84	3.00 (0.40)	2.00 (0.57)	0.20 (0.09)	9.80 (1.85)
IR60819-34-2	84	0.60 (0.11)	2.40 (0.76)	0.40 (0.18)	7.20 (0.69)
F-time		29.262***	24.500***	31.148***	63.717ns
F time x Accession		2.173ns	1.494ns	1.406ns	0.856ns
F-accession		0.245ns	1.469ns	0.963ns	1.218ns
IR 84714 H	21	1.00 (0.20)	5.60 (1.87)	0.00 (0.00)	2.80 (0.60)

IR 80559 A	21	0.80 (0.26)	8.00 (1.22)	0.00 (0.00)	2.60 (0.56)
IR 80559 B	21	0.20 (0.09)	3.40 (0.64)	0.00 (0.00)	2.00 (0.63)
IR60819-34-2 R	21	0.00 (0.00)	1.20 (0.36)	0.00 (0.00)	0.80 (0.36)
IR 84714 H	56	14.40 (2.55)	12.80 (2.51)	0.80 (0.27)	14.40 (2.55)
IR 80559 A	56	51.80 (4.71)	63.60 (4.82)	2.80 (1.11)	41.60 (4.79)
IR 80559 B	56	56.80 (6.52)	59.80 (6.34)	1.20 (0.47)	33.40 (5.53)
IR60819-34-2 R	56	33.20 (4.01)	20.20 (4.07)	0.80 (0.22)	21.60 (4.56)
IR 84714 H	84	0.80 (0.22)AB	8.40 (2.54)AB	0.40 (0.18)	7.00 (1.46)
IR 80559 A	84	2.80 (0.22)B	9.20 (1.40)B	0.00 (0.00)	18.60 (2.34)
IR 80559 B	84	2.20 (0.30)AB	3.80 (0.76)AB	0.40 (0.18)	14.60 (3.09)
IR60819-34-2 R	84	0.80 (0.17)A	2.20 (0.50)A	0.20 (0.09)	10.60 (1.33)
F-time		34.620***	18.869***	38.958***	10.220***
F time x Accession		1.324ns	1.443ns	2.029ns	0.389ns
F-accession		3.642*	3.575*	3.159ns	0.217ns
IR 85471 H	21	1.00 (0.24)	4.20 (1.00)	0.20 (0.09)	2.40 (0.50)
IR 80564 A	21	0.20 (0.09)	3.60 (0.92)	0.00 (0.00)	2.60 (0.77)
IR 80564 B	21	0.00 (0.00)	1.20 (0.54)	0.00 (0.00)	1.80 (0.61)
IR60819-34-2 R	21	0.00 (0.00)	1.20 (0.36)	0.00 (0.00)	0.80 (0.36)
IR 85471 H	56	27.20 (7.65)	26.20 (6.37)	0.20 (6.09)	34.00 (9.92)
IR 80564 A	56	29.80 (5.70)	20.60 (3.96)	1.60 (0.65)	51.00 (12.76)
IR 80564 B	56	32.40 (5.37)	27.40 (5.47)	0.20 (0.09)	28.20 (5.57)
IR60819-34-2 R	56	33.20 (4.01)	20.20 (4.07)	0.80 (0.22)	21.60 (4.56)
IR 85471 H	84	0.80 (0.17)	7.00 (0.81)	0.40 (0.18)	8.20 (1.57)
IR 80564 A	84	1.60 (0.33)	4.00 (0.82)	0.40 (0.08)	10.20 (1.28)
IR 80564 B	84	0.80 (0.26)	1.60 (0.65)	0.00 (0.00)	9.00 (2.35)
IR60819-34-2 R	84	0.80 (0.17)	2.20 (0.50)	0.20 (0.09)	10.60 (1.33)
F-time		33.285***	59.451***	9.997***	3.517*
F time x Accession		1.395ns	1.262ns	0.454ns	0.726ns
F-accession		0.246ns	1.163ns	0.468ns	2.135ns
IR 81954 H	21	2.40 (0.87)	7.80 (0.64)	0.00 (0.00)	2.40 (0.59)
IR 70369 A	21	0.60 (0.18)	6.80 (0.85)	0.00 (0.00)	2.60 (0.68)
IR 70369 B	21	0.40 (0.11)	5.60 (0.56)	0.00 (0.00)	6.00 (1.86)
IR72889-46-3-2-1 R	21	0.00 (0.00)	6.20 (0.93)	0.00 (0.00)	1.80 (0.52)
IR 81954 H	56	25.00 (3.97)	43.60 (7.79)	0.20 (0.09)	33.20 (6.35)

IR 70369 A	56	47.60 (7.92)	62.00 (6.90)	1.60 (0.65)	55.80 (9.43)
IR 70369 B	56	48.80 (2.81)	59.20 (5.66)	3.00 (0.73)	51.20 (9.13)
IR72889-46-3-2-1 R	56	35.60 (5.07)	35.40 (5.05)	1.40 (0.53)	31.80 (7.07)
IR 81954 H	84	1.20 (0.26)	7.00 (2.50)	0.00 (0.00)	3.80 (0.87)A
IR 70369 A	84	1.00 (0.14)	3.60 (1.21)	0.00 (0.00)	7.80 (1.27)AB
IR 70369 B	84	1.00 (0.14)	5.60 (0.83)	0.20 (0.09)	10.00 (1.64)B
IR72889-46-3-2-1 R	84	0.80 (0.17)	1.00 (0.36)	0.40 (0.18)	7.80 (1.86)AB
F-time		48.206***	16.436***	23.089***	19.636***
F time x Accession		0.492ns	1.061ns	0.506ns	2.022ns
F-accession		0.519ns	0.709ns	0.747ns	3.904*
IR 80637 H	21	0.40 (0.18)	5.40 (1.14)	0.00 (0.00)	2.60 (0.57)
IR 73328 A	21	0.00 (0.00)	3.00 (0.93)	0.20 (0.09)	2.20 (0.65)
IR 73328 B	21	1.80 (0.43)	3.80 (1.16)	0.00 (0.00)	3.20 (0.76)
IR73013-95-1-3-2 R	21	0.40 (0.11)	6.40 (1.84)	0.00 (0.40)	2.20 (0.77)
IR 80637 H	56	27.20 (3.70)	37.40 (5.80)	1.00 (0.38)	16.60 (3.97)
IR 73328 A	56	27.00 (6.31)	32.60 (6.72)	1.00 (0.45)	29.00 (6.59)
IR 73328 B	56	57.80 (6.28)	50.00 (8.68)	0.80 (0.29)	46.40 (9.23)
IR73013-95-1-3-2 R	56	31.60 (3.16)	15.60 (3.10)	1.20 (0.45)	11.80 (2.65)
IR 80637 H	84	0.60 (0.11)	1.60 (0.61)	0.40 (0.18)	2.80 (0.68)
IR 73328 A	84	0.80 (0.26)	1.60 (0.42)	1.20 (0.47)	10.00 (1.61)
IR 73328 B	84	0.60 (0.11)	2.20 (0.56)	0.40 (0.18)	4.40 (1.04)
IR73013-95-1-3-2 R	84	2.40 (0.86)	6.80 (2.14)	0.60 (0.20)	3.80 (1.12)
F-time		33.499***	24.607***	18.927***	6.737***
F time x Accession		0.386ns	1.330ns	2.219ns	0.348ns
F-accession		2.362ns	0.067ns	2.257ns	0.368ns
IR 82385 H	21	0.20 (0.09)	6.20 (1.41)	0.20 (0.09)	3.20 (1.02)
IR 79125 A	21	0.80 (0.36)	3.00 (0.63)	0.00 (0.00)	2.80 (0.66)
IR 79125 B	21	1.40 (0.52)	2.20 (0.62)	0.00 (0.00)	2.20 (0.63)
IR73717-46-1-3-3 R	21	1.40 (0.52)	1.80 (0.50)	0.00 (0.80)	3.80 (0.80)
IR 82385 H	56	40.40 (5.70)	29.20 (3.73)	0.60 (0.27)	38.80 (8.01)
IR 79125 A	56	68.60 (21.67)	73.40 (23.26)	3.40 (1.22)	261.00 (102.32)
IR 79125 B	56	30.20 (3.02)	22.00 (4.16)	1.00 (0.29)	40.60 (6.49)
IR73717-46-1-3-3 R	56	44.20 (1.45)	30.00 (4.06)	0.40 (0.18)	44.20 (7.24)
IR 82385 H	84	1.40 (0.23)	6.60 (1.00)	1.00 (0.31)	7.80 (1.29)

IR 79125 A	84	0.60 (0.27)	1.60 (0.32)	0.00 (0.00)	10.80 (2.62)
IR 79125 B	84	1.00 (0.35)	3.40 (0.68)	0.60 (0.00)	8.20 (1.52)
IR73717-46-1-3-3 R	84	0.60 (0.18)	3.00 (0.72)	0.20 (0.09)	7.00 (1.18)
F-time		68.898***	44.754***	3.152ns	9.207***
F time x Accession		0.495ns	0.559ns	0.837ns	0.497ns
F-accession		0.785ns	0.851ns	0.853ns	1.181ns
IR 82363 H	21	1.00 (0.24)	6.00 (0.87)	0.00 (0.00)	6.60 (1.40)
IR 68897 A	21	0.20 (0.09)	4.20 (0.94)	0.20 (0.09)	5.80 (1.30)
IR 68897 B	21	0.00 (0.00)	5.67 (2.19)	0.00 (0.00)	6.00 (2.52)
SRT 3 R	21	0.00 (0.00)	3.00 (0.44)	0.00 (0.00)	0.60 (0.27)
IR 82363 H	56	23.20 (3.37)	34.80 (5.01)	0.00 (0.00)	24.40 (4.47)
IR 68897 A	56	16.00 (3.56)	33.80 (4.83)	0.80 (0.36)	28.80 (6.38)
IR 68897 B	56	36.00 (18.08)	45.33 (30.07)	0.33 (0.33)	40.00 (10.02)
SRT 3 R	56	39.80 (9.16)	34.20 (3.36)	0.80 (0.27)	31.80 (6.91)
IR 82363 H	84	1.40 (0.39)	3.20 (0.89)	0.20 (0.09)	9.20 (1.34)
IR 68897 A	84	1.40 (0.33)	3.80 (0.57)	0.20 (0.09)	23.00 (3.12)
IR 68897 B	84	1.67 (0.88)	2.67 (1.20)	0.67 (0.67)	11.33 (1.20)
SRT 3 R	84	0.60 (0.27)	2.60 (0.58)	0.20 (0.09)	5.60 (1.05)
F-time		119.528***	14.733***	17.508***	2.288ns
F time x Accession		1.339ns	0.157ns	0.853ns	0.679ns
F-accession		1.433ns	0.234ns	0.725ns	1.091ns

1: Numbers are means (SEM) (N = 5); Degrees of freedom for Time = 2,32, Time x Accession = 6,32 and Accession = 3,16; ns = P > 0.05, * = P < 0.05, *** = P < 0.001; uppercase letters indicate homogenous accession groups based on repeated measures (Tukey P > 0.05); 2: Note that the results for BPH and WBPH have been summarized by plant-type and presented in a related paper by Horgan et al (2016); the data and information by accession, and other data from this table have not been previously published.

Table S15: Results from egg mass sampling and dissections of rice plants sampled from rice field plots at IRRI indicating the occurrence of Lepidopteran defoliators and stemborers. Results are summarized in Table 3

**Accession**	Period	**Number of Leaf folders per Plant[1,2]**	**Number of Green Hairy Caterpillars per Plant[1,2]**	**Number of PSB per Plant[1,2]**	**Number of SSB per Plant[1,2]**	**Number of YSB per Plant[1,2]**	**Proportion of Deadheart Tillers per Plant[1,2]**	**Proportion of Whitehead Tillers per Plant[1,2]**	**Number of YSB Egg Masses per Plant[1]**
IR82396H	56	0.33 (0.03)	0.03 (0.01)	0.00 (0.00)	0.00 (0.00)	0.40 (0.13)	0.03 (0.01)		0.00 (0.00)
IR80156A	56	0.19 (0.02)	0.04 (0.00)	0.00 (0.00)	0.00 (0.00)	0.30 (0.05)	0.04 (0.01)		0.40 (0.18)
IR80156B	56	0.38 (0.08)	0.05 (0.01)	0.00 (0.00)	0.00 (0.00)	0.10 (0.04)	0.02 (0.00)		0.00 (0.00)
IR46R	56	0.27 (0.03)	0.04 (0.01)	0.00 (0.00)	0.00 (0.00)	0.10 (0.04)	0.04 (0.01)		0.00 (0.00)
IR82396H	84	0.89 (0.08)	0.67 (0.14)	0.00 (0.00)	0.00 (0.00)	0.00 (0.00)	0.01 (0.00)	0.00 (0.00)	
IR80156A	84	0.54 (0.04)	0.23 (0.05)	0.00 (0.00)	0.20 (0.09)	0.00 (0.00)	0.00 (0.00)	0.02 (0.01)	
IR80156B	84	0.26 (0.03)	0.53 (0.10)	0.00 (0.00)	0.00 (0.00)	0.00 (0.00)	0.00 (0.00)	0.01 (0.00)	
IR46R	84	0.79 (0.08)	0.38 (0.07)	0.00 (0.00)	0.00 (0.00)	0.00 (0.00)	0.01 (0.01)	0.07 (0.03)	
IR82396H	98	0.55 (0.04)C	0.22 (0.03)	0.60 (0.18)	0.40 (0.18)	1.60 (0.39)AB	0.07 (0.01)	0.15 (0.02)AB	
IR80156A	112	0.22 (0.03)A	0.23 (0.05)	0.60 (0.18)	0.40 (0.18)	2.80 (0.26)A	0.11 (0.03)	0.21 (0.03)B	
IR80156B	93	0.52 (0.02)AB	0.66 (0.11)	0.00 (0.00)	0.00 (0.00)	0.20 (0.09)AB	0.00 (0.00)	0.01 (0.00)A	
IR46R	112	0.64 (0.07)BC	0.26 (0.03)	0.40 (0.11)	0.60 (0.27)	4.20 (0.55)B	0.06 (0.01)	0.26 (0.02)B	
F-Period		2.507ns	0.792ns						
F-Period x Accession		2.562ns	1.003ns	3.00	16.00				
F-Accession		5.022**	1.548ns	0.842ns	0.373ns	4.338*	0.380ns	4.959*	1.000ns
IR 82391 H	56	0.19 (0.02)	0.11 (0.04)	0.00 (0.00)	0.00 (0.00)	0.40 (0.08)	0.06 (0.01)B		0.80 (0.17)
IR 79156 A	56	0.25 (0.02)	0.05 (0.01)	0.00 (0.00)	0.00 (0.00)	0.20 (0.09)	0.02 (0.00)A		0.00 (0.00)
IR 79156 B	56	0.35 (0.02)	0.06 (0.01)	0.00 (0.00)	0.00 (0.00)	0.00 (0.00)	0.02 (0.01)AB		0.80 (0.17)
IR60819-34-2	56	0.30 (0.05)	0.02 (0.00)	0.00 (0.00)	0.00 (0.00)	0.10 (0.04)	0.01 (0.00)A		0.20 (0.09)
IR 82391 H	84	0.50 (0.07)	0.52 (0.12)	0.10 (0.00)	0.40 (0.18)	0.00 (0.00)	0.01 (0.00)	0.00 (0.00)	
IR 79156 A	84	0.50 (0.05)	0.20 (0.03)	0.20 (0.00)	0.20 (0.00)	0.20 (0.09)	0.01 (0.00)	0.01 (0.00)	
IR 79156 B	84	0.21 (0.03)	0.14 (0.02)	0.00 (0.00)	0.00 (0.00)	0.00 (0.00)	0.01 (0.00)	0.01 (0.00)	
IR60819-34-2	84	0.95 (0.08)	0.23 (0.06)	0.00 (0.00)	0.20 (0.09)	0.40 (0.11)	0.00 (0.00)	0.06 (0.01)	
IR 82391 H	93	0.30 (0.05)A	0.83 (0.12)	0.00 (0.00)	0.00 (0.00)	0.00 (0.00)A	0.03 (0.02)	0.01 (0.00)A	
IR 79156 A	98	0.33 (0.05)A	0.53 (0.09)	0.40 (0.18)	0.20 (0.09)	1.20 (0.54)AB	0.02 (0.01)	0.12 (0.01)BC	
IR 79156 B	98	0.26 (0.04)A	0.50 (0.07)	0.00 (0.00)	0.00 (0.00)	3.00 (0.32)B	0.02 (0.00)	0.23 (0.01)C	
IR60819-34-2	93	0.81 (0.11)B	0.56 (0.09)	0.40 (0.11)	0.20 (0.09)	0.40 (0.18)AB	0.00 (0.00)	0.05 (0.01)AB	
F-Period		1.307ns	7.027*						

F-Period x Accession		0.305ns	0.010ns						
F-Accession		7.795***	1.552ns	0.970ns	0.667ns	3.371*	4.578*	14.887***	2.125ns
F-Leafiness			12.355***						
IR 84714 H	56	0.24 (0.04)	0.05 (0.01)	0.00 (0.00)	0.00 (0.00)	0.00 (0.00)	0.03 (0.01)		0.40 (0.11)
IR 80559 A	56	0.32 (0.05)	0.04 (0.01)	0.00 (0.00)	0.00 (0.00)	0.00 (0.00)	0.01 (0.00)		0.00 (0.00)
IR 80559 B	56	0.39 (0.04)	0.06 (0.00)	0.00 (0.00)	0.00 (0.00)	0.10 (0.04)	0.01 (0.00)		0.00 (0.00)
IR60819-34-2 R	56	0.13 (0.02)	0.04 (0.01)	0.00 (0.00)	0.00 (0.00)	0.10 (0.04)	0.02 (0.01)		0.20 (0.09)
IR 84714 H	84	0.52 (0.04)	0.38 (0.06)	0.00 (0.00)	0.00 (0.00)	0.00 (0.00)	0.01 (0.00)	0.00 (0.00)	
IR 80559 A	84	0.65 (0.09)	0.32 (0.03)	0.20 (0.09)	0.20 (0.09)	0.00 (0.00)	0.01 (0.00)	0.00 (0.00)	
IR 80559 B	84	0.39 (0.03)	0.24 (0.02)	0.20 (0.09)	0.20 (0.09)	0.00 (0.00)	0.01 (0.00)	0.01 (0.00)	
IR60819-34-2 R	84	0.63 (0.06)	0.26 (0.06)	0.00 (0.00)	0.00 (0.00)	0.20 (0.09)	0.00 (0.00)	0.02 (0.01)	
IR 84714 H	93	0.76 (0.10)AB	0.84 (1.00)B	0.00 (1.00)	0.00 (1.00)	0.00 (0.00)Aa	0.00 (0.00)	0.00 (0.00)Aa	
IR 80559 A	112	0.23 (0.02)A	0.21 (0.05)A	0.00 (0.00)	0.40 (0.18)	6.60 (0.99)B	0.07 (0.02)	0.22 (0.02)B	
IR 80559 B	93	0.59 (0.05)AB	0.45 (0.08)AB	0.00 (0.00)	0.00 (0.00)	0.00 (0.00)Aa	0.00 (0.00)	0.04 (0.01)Aa	
IR60819-34-2 R	98	1.05 (0.07)B	0.51 (0.05)AB	0.20 (0.09)	0.20 (0.09)	2.60 (0.30)ABb	0.06 (0.01)	0.22 (0.02)Bb	
F-Period		1.194ns	1.144ns						
F-Period x Accession		3.194*	3.556*						
F-Accession		3.112*	7.842***	1.000ns	0.444ns	7.308***	0.327ns	15.185***	1.467ns
F-Height		0.581ns	9.905***						
F-Leafiness		2.530ns	15.841***						
IR 85471 H	56	0.24 (0.02)	0.02 (0.00)	0.00 (0.00)	0.00 (0.00)	0.10 (0.04)	0.01 (0.00)		0.40 (0.11)
IR 80564 A	56	0.23 (0.04)	0.04 (0.01)	0.00 (0.00)	0.00 (0.00)	0.20 (0.09)	0.04 (0.01)		0.00 (0.00)
IR 80564 B	56	0.38 (0.02)	0.11 (0.02)	0.00 (0.00)	0.00 (0.00)	0.30 (0.09)	0.03 (0.01)		0.00 (0.00)
IR60819-34-2 R	56	0.13 (0.02)	0.04 (0.01)	0.00 (0.00)	0.00 (0.00)	0.10 (0.04)	0.02 (0.01)		0.20 (0.09)
IR 85471 H	84	0.88 (0.08)	0.32 (0.06)	0.00 (0.00)	0.00 (0.00)	0.40 (0.18)	0.03 (0.01)	0.04 (0.01)	
IR 80564 A	84	0.31 (0.05)	0.43 (0.08)	0.20 (0.09)	0.20 (0.09)	0.00 (0.00)	0.01 (0.01)	0.02 (0.01)	
IR 80564 B	84	0.58 (0.07)	0.55 (0.08)	0.00 (0.00)	0.00 (0.00)	0.00 (0.00)	0.00 (0.00)	0.00 (0.00)	
IR60819-34-2 R	84	0.63 (0.06)	0.26 (0.06)	0.00 (0.00)	0.00 (0.00)	0.20 (0.09)	0.00 (0.00)	0.02 (0.01)	
IR 85471 H	93	0.89 (0.04)Cb	0.69 (0.05)	0.20 (0.09)	0.00 (0.00)	0.40 (0.11)a	0.00 (0.00)	0.04 (0.01)Aa	
IR 80564 A	112	0.15 (0.01)A	0.21 (0.02)	1.20 (0.26)	0.00 (0.00)	4.80 (0.42)	0.05 (0.01)	0.22 (0.01)B	
IR 80564 B	93	0.45 (0.05)ABa	0.56 (0.08)	0.00 (0.00)	0.00 (0.00)	0.20 (0.09)a	0.01 (0.00)	0.05 (0.01)Aa	
IR60819-34-2 R	98	1.05 (0.07)BCab	0.51 (0.05)	0.20 (0.09)	0.20 (0.09)	2.60 (0.30)b	0.06 (0.01)	0.22 (0.02)Bb	
F-Period		0.153ns	1.034ns						
F-Period x Accession		2.060ns	1.744ns						

F-Accession		10.994***	1.153ns	2.794ns	1.000ns	1.761ns (9.500***)	0.617ns	14.441***	1.467ns
IR 81954 H	56	0.26 (0.03)	0.02 (0.00)	0.00 (0.00)	0.00 (0.00)	0.10 (0.04)	0.03 (0.01)		0.20 (0.09)
IR 70369 A	56	0.38 (0.05)	0.04 (0.01)	0.00 (0.00)	0.00 (0.00)	0.20 (0.05)	0.02 (0.00)		0.00 (0.00)
IR 70369 B	56	0.36 (0.06)	0.05 (0.02)	0.10 (0.04)	0.00 (0.00)	0.10 (0.04)	0.06 (0.02)		0.00 (0.00)
IR72889-46-3-2-1 R	56	0.20 (0.02)	0.07 (0.01)	0.00 (0.00)	0.00 (0.00)	0.00 (0.00)	0.01 (0.00)		0.20 (0.09)
IR 81954 H	84	0.66 (0.11)	0.22 (1.06)	0.00 (1.00)	0.00 (0.00)	0.20 (0.09)	0.02 (0.01)	0.05 (0.02)	
IR 70369 A	84	0.64 (0.06)	0.13 (0.04)	0.00 (0.00)	0.00 (0.00)	0.00 (0.00)	0.01 (0.00)	0.01 (0.00)	
IR 70369 B	84	0.55 (0.05)	0.52 (0.03)	0.20 (0.09)	0.00 (0.00)	0.60 (0.27)	0.02 (0.01)	0.03 (0.01)	
IR72889-46-3-2-1 R	84	0.35 (0.03)	0.33 (0.08)	0.00 (0.00)	0.00 (0.00)	0.00 (0.00)	0.08 (0.03)	0.00 (0.00)	
IR 81954 H	98	0.49 (0.14)	0.45 (1.06)B	0.50 (0.14)	0.25(0.13)	3.50 (0.66)	0.01 (0.00)	0.14 (0.02)AB	
IR 70369 A	98	0.47 (0.08)	0.38 (0.08)AB	0.00 (0.00)	0.00 (0.00)	0.80 (0.17)	0.01 (0.00)	0.13 (0.01)AB	
IR 70369 B	91	0.57 (0.10)	0.34 (1.03)A	0.20 (0.09)	0.00 (0.00)	0.80 (0.36)	0.01 (0.00)	0.00 (0.00)A	
IR72889-46-3-2-1 R	112	0.17 (0.02)	0.35 (0.08)A	1.20 (0.43)	0.60 (0.18)	1.00 (0.20)	0.00 (0.00)	0.25 (0.02)B	
F-Period		1.519ns	5.824*						
F-Period x Accession		0.240ns	0.950ns						
F-Accession		1.298ns	3.974*	1.004ns	1.753ns	2.713ns (3,15)	2.842ns (3,15)	11.243***	0.667ns
F-Height								5.114*	
F-Leafiness		0.558ns	19.704***				6.829* (1,15)		
IR 80637 H	56	0.16 (0.03)	0.04 (0.01)	0.00 (0.00)	0.00 (0.00)	0.10 (0.04)	0.03 (0.01)		0.00 (0.00)
IR 73328 A	56	0.13 (0.03)	0.05 (0.01)	0.10 (0.04)	0.00 (0.00)	0.00 (0.00)	0.02 (0.01)		0.00 (0.00)
IR 73328 B	56	0.27 (0.04)	0.04 (0.01)	0.00 (0.00)	0.00 (0.00)	0.20 (0.09)	0.02 (0.01)		0.00 (0.00)
IR73013-95-1-3-2 R	56	0.15 (0.01)	0.02 (0.00)	0.00 (0.00)	0.00 (0.00)	0.00 (0.00)	0.02 (0.00)		0.00 (0.00)
IR 80637 H	84	0.61 (0.11)	0.23 (1.06)	0.00 (0.00)	0.00 (0.00)	0.00 (0.00)	0.03 (0.01)	0.10 (0.03)	
IR 73328 A	84	0.24 (0.03)	0.34 (0.05)	0.00 (0.00)	0.00 (0.00)	0.20 (0.09)	0.00 (0.00)	0.00 (0.00)	
IR 73328 B	84	0.31 (0.06)	0.36 (0.07)	0.00 (0.00)	0.00 (0.00)	0.00 (0.00)	0.01 (0.00)	0.01 (0.00)	
IR73013-95-1-3-2 R	84	0.70 (0.14)	0.20 (1.06)	0.00 (0.00)	0.00 (0.00)	0.00 (0.00)	0.01 (0.00)	0.00 (0.00)	
IR 80637 H	98	0.62 (0.05)AB	0.42 (0.08)	0.60 (0.18)	0.00 (0.00)A	2.80 (0.52)	0.01 (0.00)	0.26 (0.03)B	
IR 73328 A	98	0.14 (0.02)A	0.21 (0.04)	0.20 (0.09)	0.00 (0.00)A	0.40 (0.11)	0.01 (0.00)	0.05 (0.01)A	
IR 73328 B	91	0.12 (0.02)A	0.19 (0.02)	0.00 (0.00)	0.80 (0.26)B	2.00 (0.20)	0.00 (0.00)	0.09 (0.01)AB	
IR73013-95-1-3-2 R	112	0.71 (0.10)B	0.32 (0.06)	0.00 (0.00)	1.00 (0.24)B	1.80 (0.26)	0.02 (0.00)	0.17 (0.02)AB	
F-Period		0.249ns	3.806ns						
F-Period x Accession		0.130ns	0.865ns						
F-Accession		3.558*	1.264ns	0.924ns	3.730*	1.771ns	0.198ns (3,16)	3.931*	NA
F-Height				0.064ns	5.515*	2.949ns			

F-Leafiness		0.017ns	11.626***						
IR 82385 H	56	0.15 (0.02)	0.06 (0.02)	0.10 (0.04)	0.00 (0.00)	0.70 (0.21)	0.05 (0.01)		0.20 (0.09)
IR 79125 A	56	0.16 (0.02)	0.05 (0.01)	0.00 (0.00)	0.00 (0.00)	0.00 (0.00)	0.01 (0.00)		0.00 (0.00)
IR 79125 B	56	0.38 (0.04)	0.04 (0.00)	0.00 (0.00)	0.00 (0.00)	0.10 (0.04)	0.02 (0.01)		0.20 (0.09)
IR73717-46-1-3-3 R	56	0.26 (0.02)	0.07 (0.01)	0.00 (0.00)	0.00 (0.00)	0.50 (0.10)	0.05 (0.01)		0.00 (0.00)
IR 82385 H	84	0.63 (0.05)	0.55 (0.12)	0.20 (0.00)	0.20 (0.00)	0.00 (0.00)	0.00 (0.00)	0.02 (0.00)	
IR 79125 A	84	0.16 (0.02)	0.47 (0.06)	0.00 (0.00)	0.00 (0.00)	0.80 (0.36)	0.02 (0.01)	0.04 (0.02)	
IR 79125 B	84	0.61 (0.04)	0.57 (0.17)	0.00 (0.00)	0.00 (0.00)	0.20 (0.09)	0.00 (0.00)	0.03 (0.01)	
IR73717-46-1-3-3 R	84	0.67 (0.08)	0.47 (0.06)	0.00 (0.00)	0.00 (0.00)	0.20 (0.09)	0.01 (0.01)	0.05 (0.02)	
IR 82385 H	112	0.39 (0.02)	0.25 (0.05)	0.60 (0.11)	0.80 (0.17)	5.00 (0.73)B	0.03 (0.01)	0.25 (0.02)B	
IR 79125 A	98	0.29 (0.03)	0.43 (0.05)	0.40 (0.18)	0.20 (0.09)	2.40 (0.46)AB	0.01 (0.01)	0.10 (0.01)A	
IR 79125 B	93	0.39 (0.03)	0.64 (0.07)	0.00 (0.00)	0.20 (0.09)	0.00 (0.00)A	0.01 (0.00)	0.00 (0.00)A	
IR73717-46-1-3-3 R	93	0.61 (0.07)	0.73 (0.06)	0.40 (0.18)	1.20 (0.26)	1.20 (0.22)A	0.01 (0.00)	0.06 (0.01)A	
F-Period		0.001ns	2.666ns						
F-Period x Accession		2.597ns	1.760ns						
F-Accession		2.071ns	1.141ns	0.667ns	1.714ns	4.570*	1.158ns	13.624***	0.667ns
F-Tillers		4.121*	8.205**						
IR 82363 H	56	0.36 (0.03)	0.05 (0.01)	0.00 (0.00)	0.00 (0.00)	0.20 (0.05)	0.02 (0.00)		0.20 (0.09)
IR 68897 A	56	0.25 (0.02)	0.03 (0.01)	0.00 (0.00)	0.00 (0.00)	0.00 (0.00)	0.01 (0.00)		0.40 (0.18)
IR 68897 B	56	0.34 (0.09)	0.10 (0.04)	0.00 (0.00)	0.00 (0.00)	0.50 (0.17)	0.04 (0.01)		0.67 (0.38)
SRT 3 R	56	0.27 (0.04)	0.10 (0.03)	0.00 (0.00)	0.00 (0.00)	0.10 (0.04)	0.03 (0.01)		0.20 (0.09)
IR 82363 H	84	0.77 (0.06)	0.47 (0.00)	0.00 (0.00)	0.00 (0.00)	0.00 (0.00)	0.01 (0.01)	0.01 (0.01)	
IR 68897 A	84	0.31 (0.05)	0.17 (0.04)	0.00 (0.00)	0.00 (0.00)	0.20 (0.09)	0.00 (0.00)	0.02 (0.01)	
IR 68897 B	84	0.57 (0.11)	0.51 (1.09)	0.00 (1.00)	0.00 (0.00)	0.00 (0.00)	0.00 (0.00)	0.00 (0.00)	
SRT 3 R	84	0.70 (0.10)	0.47 (1.00)	0.00 (1.00)	0.00 (0.00)	0.40 (0.18)	0.00 (0.00)	0.12 (0.05)	
IR 82363 H	93	0.55 (0.05)B	0.70 (0.00)B	0.00 (0.00)	0.40 (0.18)	0.20 (0.09)A	0.01 (0.00)	0.03 (0.01)A	
IR 68897 A	112	0.12 (0.01)A	0.16 (0.04)A	0.00 (0.00)	0.00 (0.00)	6.40 (0.01)B	0.05 (0.01)	0.15 (0.01)AB	
IR 68897 B	93	0.42 (0.04)AB	0.49 (0.11)A	0.00 (0.00)	0.00 (0.00)	1.33 (0.51)AB	0.01 (0.01)	0.05 (0.01)A	
SRT 3 R	112	0.48 (0.04)AB	0.28 (0.06)A	0.60 (0.27)	0.20 (0.09)	4.40 (0.64)B	0.07 (0.02)	0.28 (0.03)B	
F-Period		6.052*	2.134ns						
F-Period x Accession		0.110ns	1.334ns						
F-Accession		5.390***	4.464*	0.843ns	0.583ns	3.590*	0.999ns	9.260*** (3,14)	0.333ns
F-Leafiness		2.106ns	6.820*				7.655* (3,13)		

1: Numbers are means (SEM) (N = 5); Degrees of freedom for Time = 2,32, Time x Accession = 6,32 and Accession = 3,16 except where indicated by square brackets; ns = P > 0.05, * = P < 0.05, ** = P < 0.01, *** = P < 0.001; numbers in grey font were not included in the analyses because of low numbers of observations and frequent zeros; uppercase letters indicate homogenous accession groups based on all plant types, lowercase letters indicate groups based on analyses with A-lines excluded (Tukey P > 0.05); 2: Note that the results for Leaffolders, Green hairy caterpillars, PSB, SSB, YSB, deadhearts and whiteheads have been summarized by plant-type and presented in a related paper by Horgan et al (2016); the data and information by accession and other data from this table have not been previously published.
